# The asymmetric Henry reaction as synthetic tool for the preparation of the drugs linezolid and rivaroxaban

**DOI:** 10.3762/bjoc.18.46

**Published:** 2022-04-14

**Authors:** Martin Vrbický, Karel Macek, Jaroslav Pochobradský, Jan Svoboda, Miloš Sedlák, Pavel Drabina

**Affiliations:** 1 Institute of Organic Chemistry and Technology, Faculty of Chemical Technology, University of Pardubice, Studentská 573, 532 10 Pardubice, Czech Republic

**Keywords:** asymmetric Henry reaction, enantioselective catalysis, linezolid, oxazolidine-2-one derivatives, rivaroxaban

## Abstract

The human drugs – the antibiotic linezolid (**1**) and the anticoagulant rivaroxaban (**2**) – belong among modern pharmaceutics, which contain an oxazolidine-2-one moiety bearing a stereogenic center. The chirality of these drugs is a fundamental attribute for their biological activity. Herein, one of the efficient asymmetric syntheses of these drugs was studied in detail. Highly enantioselective catalysts were tested in the key step of the synthetic procedure, i.e., the asymmetric Henry reaction, under different reaction conditions, using several starting aldehydes. The corresponding nitroaldols as chiral intermediates in the syntheses of these drugs were obtained in high yields and enantiomeric excesses of up to 91% ee.

## Introduction

**Figure 1:**
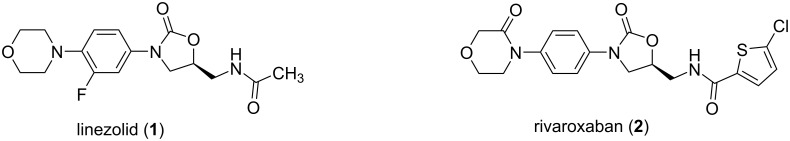
The structure of the oxazolidine-2-one-containing drugs linezolid (**1**) and rivaroxaban (**2**).

Oxazolidine-2-one derivatives represent an important branch of pharmaceutical substances [1–3]. This class includes for instance oxazolidine-type antibiotics [3], e.g., linezolid (**1**) [4] (sold under the trade name Zyvox^®^ (Figure 1) or tedizolid [5] (sold under the trade name Sivextro^®^), and the anticoagulant rivaroxaban (**2**) [6–7] (Figure 1), a member of DOACs (direct oral anticoagulants). All these human drugs can be considered as modern medicaments, which were developed and approved during the past three decades [8]. The chirality of the mentioned oxazolidine-2-ones is a crucial factor for their therapeutic effect, because only a single enantiomer affords the desired biological activity. Hence, only the *S*-enantiomer of rivaroxaban (**2**) (sold under trade name Xarelto^®^) exhibits a strong inhibitory activity against coagulant factor Xa, whereas the *R*-enantiomer is almost inactive (IC_50_ = 0.7 nM for *S* vs 2300 nM for *R*) [7]. Similarly, in the case of the oxazolidine-2-one antibiotics only the *S*-enantiomers are able to block bacterial ribosomes, which leads to the prevention of translation processes in bacteria [9–10]. With regards to these facts, a high enantiomeric purity is one of the fundamental requirements in the production of such pharmaceutical substances, because the reduction of the amount of the undesirable stereoisomer to a minimum can suppress possible side effects.

The oxazolidine-2-one-type drugs are usually prepared following synthetic methods that utilize available chiral building blocks (e.g., epichlorohydrine, glycidol, 3-chloropropane-1,2-diol, etc.) [11]. Beside this, approaches in which asymmetric synthesis is included are also applicable. Recently, the utilization of an asymmetric Henry reaction for the preparation of two oxazolidine-type drugs, namely linezolid (**1**) and rivaroxaban (**2**), has been described [12–13]. These published papers confirmed that the application of the asymmetric Henry reaction represents a promising alternative route for the feasible production of these compounds. Nevertheless, the studies provided only preliminary results, because they included only one enantioselective catalyst in the preparation of rivaroxaban (**1**) [12] and the study of the linezolid (**2**) synthesis used only commercially unavailable and poorly enantioselective catalysts (max 72% ee) [13].

**Figure 2:**
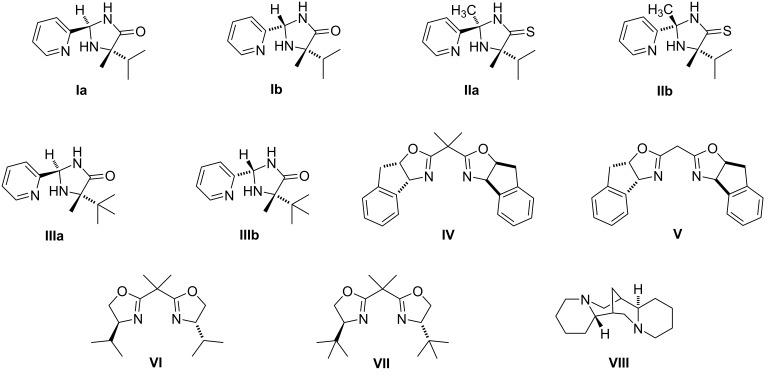
Overview of the chiral ligands that were used for the study of the asymmetric Henry reaction.

In this paper, we focused on the application of the asymmetric Henry reaction for the preparation of the oxazolidine-2-one-type drugs linezolid (**1**) and rivaroxaban (**2**). The main aim of this study was the evaluation of the catalytic activity and enantioselectivity of several established enantioselective catalysts applicable to the asymmetric Henry reaction, which were used for the preparation of chiral intermediates of these drugs. Various highly efficient catalysts based on copper complexes of different types of chiral ligands, 2-(pyridin-2-yl)imidazolidine-4-ones (**I**–**III**), bis-oxazolines (**IV**–**VII**), or diamine (**VIII**) were chosen for the study (Figure 2). Furthermore, the modification of the structure of the prochiral aldehyde intermediates **15** and **19** was also performed with the aim to increase the enantiomeric purity of the corresponding nitroaldol products **21–26**. The structural modification consisted in the introduction of different alkyl moieties to the carbamate functional group of the aldehyde intermediates **15**–**20**. As bulky and/or chiral alkyl groups we considered *tert*-butyl, ʟ-menthyl, and (−)-bornyl in this study.

## Results and Discussion

**Scheme 1:**
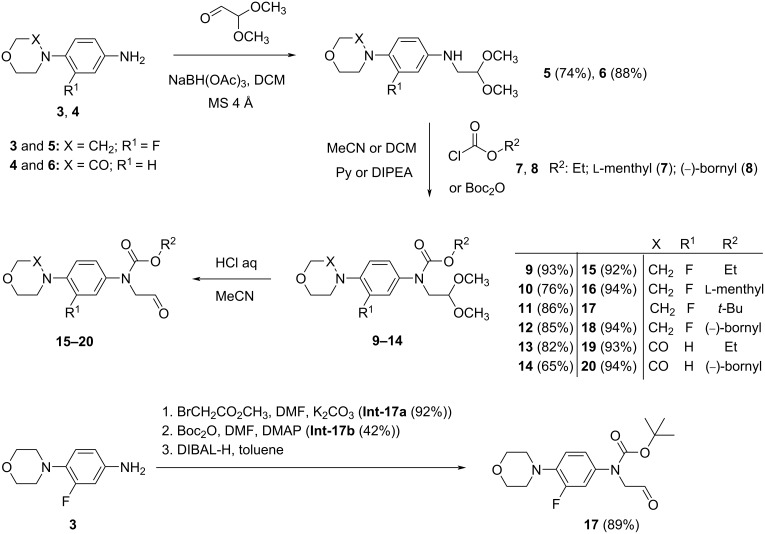
Syntheses of aldehydes **15–20**.

The aldehydes **15**–**20** were prepared by analogous methods, which were described previously [12–13]. The starting ʟ-menthyl (**7**) and (−)-bornyl chloroformate (**8**) were obtained according to the modified synthetic procedure [14]. Here, it was included the chromatographic purification of the final chloroformates, which led to removing of corresponding alkyl chlorides formed as byproducts. The aldehyde **17** was prepared by a different way, because the acid-catalyzed hydrolysis of its acetal intermediate **11** was accompanied with simultaneous cleavage of the Boc group. However, attempts of achieving a selective deacetalation of **11** by the treatment with several reagents (e.g., I_2_/acetone [15], FeCl_3_·6H_2_O/acetaldehyde [16], Ce(OTf)_3_ [17]) were unsuccessful. Therefore, an alternative synthesis [18] was utilized, which consisted of alkylation of aniline **3** by methyl bromoacetate, followed by introduction of the Boc group into intermediate **Int-17a**, and final reduction of **Int-17b** with DIBAL-H (Scheme 1).

For the study of the asymmetric Henry reaction aldehydes **15**–**20**, nitromethane, and highly enantioselective catalysts based on copper(II) complexes with chiral nitrogen ligands were chosen. Generally, chiral copper complexes possess many advantages valuable for the pharmaceutical industry. They exhibit low toxicity (compared to other metal-based complexes) and many of them exist in forms suitable for recycling [19]. Therefore, they represent a very useful tool for diverse asymmetric transformations, including the Henry reaction. The pilot study of the synthesis of rivaroxaban through an asymmetric Henry reaction [12] described the application of only one copper complex with a 2-(pyridin-2-yl)imidazolidin-4-one derivative. Here, we extended the series of catalysts to include copper complexes with another six 2-(pyridin-2-yl)imidazolidin-4-ones **Ia,b**–**IIIa,b** [20–22], four bisoxazolines **IV**–**VII** [23–24], and as chiral diamine, the alkaloid (+)-sparteine **VIII** [25] (Figure 2). All Henry reactions were performed on sub-millimolar scales in isopropyl alcohol (IPA). The obtained products **21**–**26** were separated from the starting aldehydes **15**–**20** by column chromatography. The reaction conditions (i.e., temperature, reaction time, amount of catalyst, and solvent) were adopted from the pilot study [12] for relevant comparison of the catalysts’ characteristics. Subsequently, the reaction temperature and loading of the catalyst were tuned using aldehyde **15** to achieve a satisfactory chemical yield and ee for the nitroaldol product **21** (Table 1).

**Table 1:** Asymmetric Henry reactions of aldehyde **15** with nitromethane under various conditions.

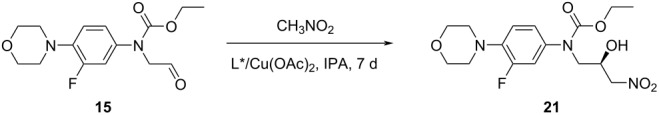				

Ligand	Cat. loading [mol %]	Temp. [°C]	Conversion^a^ [%] (yield^b^ [%])	ee^c^ [%]

**Ia**	5	20	94 (83)	85 (*R*)
**Ia**	5	6	55 (51)	88 (*R*)
**Ia**	10	6	78 (72)	88 (*R*)

**Ib**	5	20	65 (49)	68 (*S*)

**IIa**	5	20	69 (65)	68 (*R*)
**IIa**	5	6	37 (33)	80 (*R*)
**IIa**	10	6	47 (40)	86 (*R*)

**IIb**	5	20	67 (65)	66 (*S*)
**IIb**	5	6	24 (20)	71 (*S*)
**IIb**	10	6	26 (19)	69 (*S*)

**IIIa**	5	20	87 (85)	81 (*R*)
**IIIa**	5	6	52 (44)	80 (*R*)
**IIIa**	10	6	70 (63)	86 (*R*)

**IIIb**	5	20	31 (26)	38 (*S*)

**IV**	5	20	86 (78)	88 (*R*)
**IV**	5	6	59 (54)	85 (*R*)
**IV**	10	6	71 (62)	89 (*R*)

**V**	5	20	29 (23)	45 (*R*)
**V**	5	6	10 (–)	–

**VI**	5	20	82 (78)	78 (*S*)
**VI**	5	6	40 (36)	80 (*S*)

**VII**	5	20	46 (36)	46 (*S*)
**VII**	5	6	43 (35)	54 (*S*)

**VIII**	5	20	40 (38)	9 (*S*)

^a^The yield was determined by ^1^H NMR analysis of the crude product. ^b^Isolated yield after column chromatography. ^c^The enantiomeric excess was determined by chiral HPLC.

As can be seen from the results summarized in Table 1, the highest enantioselectivity was achieved with the copper(II) complexes of ligands **Ia**, **IIa**, **IIIa**, and **IV**. Fortunately, these catalysts provided the *R*-enantiomer of nitroaldol **21** as the major product, which can be subsequently transformed to *S*-linezolid (**1**) (the active stereoisomer). On the other hand, the catalysts derived from 2-(pyridin-2-yl)imidazolidine-4-ones **Ib**–**IIIb**, bisoxazoline ligands **V**–**VII**, and (+)-sparteine (**VIII**) showed only insufficient enantioselectivity and therefore, they were excluded from further studies. A higher catalyst loading (10 mol %) slightly increased the enantioselectivity in some cases (i.e., with ligands **IIa**, **IIIa**, and **IV**) and, expectedly, it enabled the achievement of a higher chemical yield. Performing of the reaction at room temperature also increased the chemical yield, however, a certain drop of the ee was observed, especially in the case of catalysts derived from ligands **IIa** and **VII**. From these results, 10 mol % of the catalyst derived from ligands **Ia**, **IIa**, **IIIa**, and **IV** and a reaction temperature of 6 °C were identified as the optimal reaction conditions for the studied asymmetric Henry reaction.

**Scheme 2:**
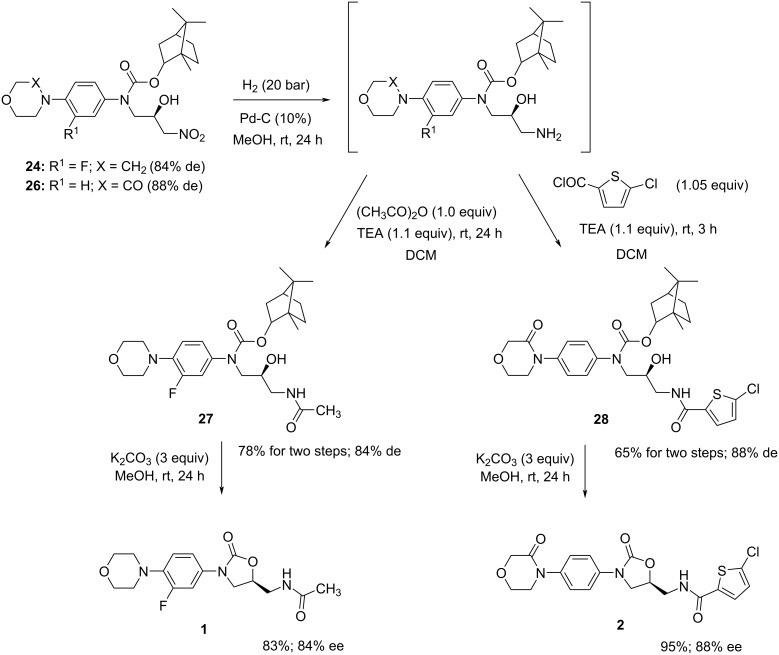
Synthesis of linezolid (**1**) and rivaroxaban (**2**) from nitroaldols **24** or **26**.

Further, the asymmetric Henry reaction of the other aldehydes **16**–**20** was studied (Table 2). The bulky (R^2^ = *t*-Bu) or chiral (R^2^ = ʟ-menthyl or (−)-bornyl) alkoxy groups (derived from relatively inexpensive and readily available alcohols) were introduced into the carbamate moiety instead of an ethyl group as in aldehydes **15** and **19**. Here, the influence of this structural modification of the starting aldehydes on the enantioselectivity of the Henry reaction was examined. Moreover, the resulting nitroaldols **22**, **24**, and **26** were formed as a pair of epimers, and therefore, a possible separation of the individual stereoisomers of these compounds was assumed. Hence, it should be noted that the R^2^O- part of the carbamate group does not modify the structure of linezolid (**1**), because this molecular moiety is cleaved during the intramolecular nucleophilic substitution in the final reaction step (Scheme 2). The catalysts derived from ligands **Ia**, **IIa**, **IIIa**, and **IV** were also tested in the asymmetric Henry reaction using the substrates **19** and **20**, which afforded the chiral intermediates **25** and **26** that are applicable for the rivaroxaban (**2**) synthesis.

**Table 2:** Asymmetric Henry reactions of aldehydes **16–20** with nitromethane catalyzed by copper(II) complexes of **Ia**, **IIa**, **IIIa**, and **IV**.

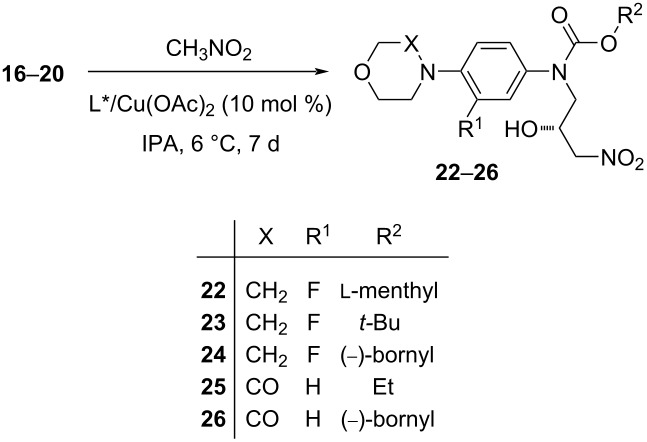				

Nitroaldol R^2^		Ligand	Conversion^a^ [%] (yield^b^ [%])	ee or de^c,d^ [%]

**22**	ʟ-menthyl	**Ia**	63 (60)	89 (*R*)
**22**		**IIa**	49 (41)	87 (*R*)
**22**		**IIIa**	38 (27)	83 (*R*)
**22**		**IV**	72 (65)	89 (*R*)

**23**	*t*-Bu	**Ia**	80 (77)	85 (*R*)
**23**		**IIa**	62 (54)	85 (*R*)
**23**		**IIIa**	72 (59)	86 (*R*)
**23**		**IV**	73 (62)	87 (*R*)

**24**	(−)-bornyl	**Ia**	70 (66)	87 (*R*)
**24**		**IIa**	63 (58)	88 (*R*)
**24**		**IIIa**	62 (55)	88 (*R*)
**24**		**IV**	79 (74)	90 (*R*)

**25**	Et	**Ia**	91 (88)	86 (*R*)
**25**		**IIa**	72 (60)	83 (*R*)
**25**		**IIIa**	55 (47)	86 (*R*)
**25**		**IV**	80 (69)	90 (*R*)

**26**	(−)-bornyl	**Ia**	65 (47)	86 (*R*)
**26**		**IIa**	44 (38)	83 (*R*)
**26**		**IIIa**	44 (34)	88 (*R*)
**26**		**IV**	67 (63)	91 (*R*)

^a^The yield was determined by ^1^H NMR analysis of the crude product. ^b^Isolated yield after column chromatography. ^c^The ee or de values were determined by chiral HPLC. ^d^The configuration at the newly formed stereocenter of the prevailing stereoisomer.

From the results summarized in Table 2 it follows that the carbamate substituents in the aldehydes **16**–**20** do not affect the enantioselectivity or diastereoselectivity. All nitroaldol products **22**–**26** were obtained with ee (or de) values in the range of 83–91%. This observation was confirmed in a separate experiment, where the aldehyde **16** bearing the substituent R^2^ = ᴅ-menthyl (opposite sense of chirality) was applied. This substrate was transformed to the corresponding nitroaldol **22** by the action of the complex Cu(OAc)_2_/**Ia** with practically identical de of 88%. On the other hand, the interpretation of chemical yields is rather indistinct. Generally, the copper(II) complexes of ligands **Ia** and **IV** exhibited a higher catalytic activity than the catalysts derived from **IIa** and **IIIa**. Thus, the yields achieved by catalysts derived from **Ia** and **IV** can be considered as satisfactory for almost all nitroaldols **21**–**26**. The influence of the substituent R^2^ differed in the type of the substrate. In the cases of nitroaldols **21**–**24** leading to linezolid (**1**) we found higher chemical yields for derivatives **23** (R^2^ = *t*-Bu) and **24** (R^2^ = (−)-bornyl) (62–80%) than for derivatives **21** (R^2^ = Et) and **22** (R^2^ = ʟ-menthyl) (38–78%). However, in the cases of nitroaldols **25** and **26**, affording subsequently rivaroxaban (**2**), we obtained higher chemical yields for derivative **25** (R^2^ = Et, 55–91%) than for **26** (R^2^ = (−)-bornyl, 44–67%). For these findings we do not have any reliable explanation.

Next, the synthetic method [12–13] for the preparation of the target oxazolidine-2-one drugs **1** and **2** from the new chiral intermediates was verified (Scheme 2). For this study, the bornyl derivatives **24** and **26** were chosen. The reduction of the nitro groups in **24** and **26** via the hydrogenation procedure proceeded smoothly with almost quantitative yields; the amine intermediates were immediately used in the next step. Hence, the *N*-acylation reactions were performed by the action of the corresponding acylating reagent (1.0 equiv) in DCM and in the presence of TEA (1.1 equiv). The amides **27** and **28** were obtained with moderate yields (78% for **27** and 65% for **28**) – values that are comparable to those previously described for the analogous ethyl derivatives [12–13]. Finally, the base-catalyzed intramolecular transesterification (cyclization) led to the desired products **1** and **2**. In the case of amides **27** and **28**, the reaction conditions for the cyclization were slightly modified, i.e., the reaction time was prolonged to 24 h and the precipitated product was washed with hexane to remove traces of borneol. No changes in the de were observed and the presence of the major *S*-enantiomer in the products **1** and **2** was confirmed by chiral HPLC analysis.

Moreover, an enhancement of the abundance of the major epimer in the nitroaldols **22**, **24**, and **26** as well as the amides **27** and **28** was examined. Generally, epimers represent pairs of stereoisomers that are easier to separate by standard techniques than enantiomers. In particular, an exploration of convenient chromatographic conditions was performed. Unfortunately, none of the conditions was successful even though all derivatives were tested. Subsequently, the separation of the epimers by recrystallization was tested. For this purpose, the nitroaldols **24** and **26** were prepared at a 10 mmol scale (ca. 4 g) using 10 mol % of the catalyst Cu(OAc)_2_/**IV** at 20 °C to achieve high yields of the products (**24**: 92% and **26**: 84%). The nitroaldols **24** and **26** were obtained in these scale-up experiments with de values of 84% and 88%, respectively. Hence, the higher temperature led to a slight drop of the de, nevertheless, the higher amount of the undesired diastereomer was considered as convenient here, in terms of the study of its separation. The nitroaldols **24** and **26** were isolated as an oil/waxy solid material, what made the attempts of recrystallization impossible. On the other hand, the corresponding amides **27** and **28** were obtained as fine crystalline solids. Their crystallization was successfully performed from several solvents. However, the de values of the isolated recrystallized material were practically the same as the one of the starting material in all cases. Further, the separation via kinetic resolution in the final reaction step was also examined. The course of the re-esterification was stopped at a conversion of ca 50% and the de values were determined. Unfortunately, no difference between the de values of the starting amides **27** and **28** and the final drugs **1** and **2** was found.

## Conclusion

In conclusion, the synthetic approach to the preparation of the antibiotic linezolid (**1**) and the anticoagulant rivaroxaban (**2**) based on the asymmetric Henry reaction was studied in detail. A series of 11 efficient enantioselective catalysts was tested to obtain the corresponding nitroaldol **21** in an enantiomeric excess as high as possible. Four of them based on the chiral ligands **Ia**, **IIa**, **IIIa**, and **IV** were identified as the most effective catalysts. They exhibited mutually comparable enantioselectivities in the range of 83–91% ee. It was found, that the enantioselectivity does not vary with the substitution in the carbamate group of the used aldehydes **15**–**20**. However, all nitroaldols **21**–**24** prepared as chiral intermediates suitable for the linezolid (**1**) synthesis were obtained with higher ee (or de) values (83–90%) than in the previously published study (up to 72% ee) [13]. The introduction of a chiral moiety into the structure of aldehydes **16**, **18** and **20** led to the formation of nitroaldols **22**, **24**, and **26** as pairs of epimers. Unfortunately, the attempts of separating the minor epimer were unsuccessful. From this point of view, further efforts on the separation of the remaining minor (5–8%) inactive (*R*)-stereomer in the final linezolid (**1**) or rivaroxaban (**2**) should be continued to make this synthetic approach commercially viable.

## Supporting Information

File 1 General information and experimental data of all isolated products, copies of ^1^H and ^13^C NMR spectra for products and HPLC chromatograms.
