# Supplementary material for: The asymmetric Henry reaction as synthetic tool for the preparation of the drugs linezolid and rivaroxaban
Source: Beilstein J Org Chem. 2022 Apr 14;18:438–45. doi: 10.3762/bjoc.18.46 (PMC9039528; doi:10.3762/bjoc.18.46)
Supplement: File 1 — General information and experimental data of all isolated products, copies of 1H and 13C NMR spectra for products and HPLC chromatograms. [file Beilstein_J_Org_Chem-18-438-s001.pdf]

BEILSTEIN JOURNAL OF ORGANIC CHEMISTRY

Supporting Information

for

The asymmetric Henry reaction as synthetic tool for the preparation of the drugs linezolid and rivaroxaban

Martin Vrbický, Karel Macek, Jaroslav Pochobradský, Jan Svoboda, Miloš Sedlák and Pavel Drabina

Beilstein J. Org. Chem. **2022,** *18,* 438–445. doi:10.3762/bjoc.18.46

General information and experimental data of all isolated products, copies of ^{1}H and ^{13}C NMR spectra for products and HPLC chromatograms

License and Terms: This is a supporting information file under the terms of the Creative Commons Attribution License (https://creativecommons.org/licenses/by/4.0). Please note that the reuse, redistribution and reproduction in particular requires that the author(s) and source are credited and that individual graphics may be subject to special legal provisions.
The license is subject to the *Beilstein Journal of Organic Chemistry* terms and conditions: (https://www.beilstein-journals.org/bjoc/terms)

Table of contents

1. Experimental S2
1.1. General procedures S2
1.2. Synthesis S2
2. NMR spectra S13
3. HPLC data S72
4. References S80

1. Experimental procedures

1.1. General procedures

The starting chemicals and solvents were obtained from Acros Organics or Fluorochem and were used without further purification. Column chromatography was performed using 60 Å (60–200 µm) silica gel. TLC was performed on aluminum-backed silica gel plates (Merck DC, Alufolien Kieselgel 60 F_{254}) with spots visualized by UV light. The melting point temperatures are uncorrected. The IR spectra were measured at room temperature using a Thermo Scientific Nicolet iS50 FTIR Spectrometer with ATR technique, the resolution was 4 cm^{-1} and FTIR data are presented in cm^{-1}. ^{1}H NMR spectra were recorded on a Bruker Avance 400 instrument (400.13 MHz for ^{1}H) or a Bruker Ascend 500 instrument (500.13 MHz for ^{1}H). Chemical shifts δ were referenced to the residual peak of $CDCl_3$ at 7.26 ppm or DMSO-d_6 at 2.50 ppm. The ^{13}C NMR spectra were calibrated with respect to the middle signal in the triplet of $CDCl_3$ (δ = 77.23 ppm). High-resolution mass spectra were measured on a Thermo Fisher Scientific MALDI LTQ Orbitrap instrument. The used matrix was a 0.2 M solution of 2,5-dihydroxybenzoic acid (DHB) in MeCN/H_2O 95:5. The Spectra were calibrated with respect to the used matrix. The optical rotation was measured on a Perkin–Elmer 341 instrument and the concentration *c* is given in g/100 mL. HPLC analyses were performed on a Watrex HPLC instrument with UV–vis DAD (200–800 nm) SYKAM 3240 and with chiral Daicel columns Chilacel OD-H, Chiralpak AD-H, Chiralpak IA, and Chiralpak AS-H (250 mm × 4.6 mm). Hydrogenations were performed in a pressure vessel Berghof BR-100.

1.2. Synthesis

Linezolid (1)

To a solution of amide **27** (1.48 g, 3 mmol) in MeOH (15 mL) was added anhydrous K_2CO_3 (1.23 g, 9 mmol) and the mixture was stirred at room temperature for 24 h. The course of reaction was monitored by TLC (SiO_2; R_f 0.34; *n*-hexane/acetone 3:1 (v/v)). The solvent was evaporated under reduced pressure and the residue was diluted with water (ca. 20 mL). The mixture was extracted with DCM (3 × 10 mL) and the combined organic layers were dried with Na_2SO_4. The solvent was evaporated under reduced pressure and the residue was purified by column chromatography. Yield: 840 mg (83%); white crystalline solid; mp 181.3–182.2 °C; R_f 0.34 (SiO_2; *n*-hexane/acetone (v/v; 3:1); $[\alpha_D^{20}]$ = –12.7 (*c* 0.88; DCM, 84 % *ee*) (Ref. [6]: $[\alpha_D^{20}]$ = –8.8 (*c* 1.0; $CHCl_3$, 99.5 % *ee*)). ^{1}H NMR (500 MHz, $CDCl_3$): δ 7.36 (m, 1H), 7.03 (m, 1H), 6.93 (bs, 1H), 6.85 (m, 1H), 4.73 (bs, 1H), 3.97 (t, 1H, *J* = 8.9 Hz), 3.81 (m, 4H), 3.72 (t, 1H, *J* = 7.8 Hz), 3.59 (m, 2H), 2.98 (m, 4H), 1.96 (s, 3H). ^{13}C NMR (125 MHz, $CDCl_3$): δ 171.6, 155.4 (d, *J* = 246.4 Hz), 154.7, 136.5 (d, *J* = 8.7 Hz), 133.0 (d, *J* = 10.5 Hz), 118.9 (d, *J* = 3.7 Hz), 114.0 (d, *J* = 2.8 Hz), 107.5 (d, *J* = 26.3 Hz), 72.2, 67.0, 51.0 (d, *J* = 1.6 Hz), 47.7, 41.9, 23.0. HR-MALDI-MS (DHB): Calcd for $C_{16}H_{20}FN_3O_4+H^+$ *m/z* 338.15161 ($[M+H]^+$), found 338.15159. Enantiomeric excess was determined by HPLC – Chiralpak IA, *n*-hexane/IPA (v/v; 70/30); flow rate 0.8 mL/min; λ = 254 nm: *S*-enantiomer t_r = 15.51 min; *R*-enantiomer t_r = 17.48 min.

Rivaroxaban (2)

Compound **2** was prepared according to the modified method described in ref. [5]. To a solution of amide **28** (2.35 g, 4 mmol) in MeOH (25 mL) was added anhydrous K_2CO_3 (1.66 g, 12 mmol) and the mixture was stirred at room temperature for 24 h. The course of reaction was monitored

by TLC (SiO_2; R_f 0.45; acetone/DCM 1:1 (v/v)). The suspension formed was filtered off, the white crystalline solid was washed with *n*-hexane (3 × 20 mL), subsequently with water (2 × 20 mL) and dried *in vacuo*. Yield: 1.67 g (96%); white crystalline solid, mp 205.8–208.2 °C; $[\alpha_D^{20}]$ = –38.0 (*c* 0.31; DMSO, 88 % *ee*). (Ref. [7]: $[\alpha_D^{20}]$ = –41 (*c* 0.3; DMSO, 99.5 % *ee*)). ^{1}H NMR (DMSO-d_6; 500 MHz): δ 8.97 (bt, *J* = 5.5 Hz, 1H), 7.68 (d, *J* = 4.0 Hz, 1H), 7.55 (m, 2H), 7.40 (m, 2H), 7.19 (d, *J* = 4.0 Hz, 1H), 4.84 (m, 1H), 4.21–4.16 (m, 3H), 3.97 (t, *J* = 5.0 Hz, 2H), 3.85 (m, 1H), 3.71 (m, 2H), 3.60 (m, 2H); ^{13}C NMR (DMSO-d_6; 125 MHz): δ 166.0, 160.8, 154.1, 138.5, 137.1, 136.5, 133.3, 128.4, 128.2, 125.9, 118.3, 71.3, 67.7, 63.5, 49.0, 47.4, 42.2. HR-MALDI-MS (DHB): Calcd. for $C_{19}H_{18}ClN_3O_5S+H^+$ ($[M+H]^+$) 436.07340, found 436.07368. Enantiomeric excess was determined by HPLC – Chiralcel OD-H, *n*-hexane/IPA (v/v; 50/50); flow rate 1.0 mL/min; λ = 254 nm: *R*-enantiomer t_r = 22.3 min; *S*-enantiomer t_r = 30.5 min.

3-Fluoro-4-(morpholine-4-yl)aniline (3)

This compound was prepared according to the procedure described in ref. [2]. To a solution of morpholine (2.6 mL, 30 mmol) and DIPEA (5.1 mL, 30 mmol) in EtOAc (15 mL) was added dropwise 3,4-difluoronitrobenzene (3.0 mL, 27 mmol). The mixture was stirred at room temperature for 24 h. The suspension formed was diluted with water (20 mL) and extracted with a mixture of DCM and EtOAc 1:4 (v/v, 3 × 50 mL). The combined extracts were dried over $MgSO_4$ and the solvents were evaporated *in vacuo*. The residue was recrystallized from EtOH to give 5.81 g (95%) of 3-fluoro-4-(morpholine-4-yl)nitrobenzene as yellow crystals. Next, a mixture of 3-fluoro-4-(morpholine-4-yl)nitrobenzene (5.3 g, 24 mmol) and Pd-C (100 mg; 10 wt %) in MeOH (40 mL) and THF (10 mL) was stirred in a hydrogen atmosphere (1 bar) at room temperature for 24 h. The catalyst was removed by filtration and the solvents were evaporated *in vacuo*. The aniline **3** was obtained as white crystalline solid, yield 4.52 g (96%); mp 122.3–122.9 °C; (SiO_2; R_f 0.35; *n*-hexane /EtOAc 2:1 (v/v)); ^{1}H NMR (400 MHz, $CDCl_3$): δ 6.78 (t, 1H, *J* = 9.0 Hz), 6.43–6.37 (m, 2H), 3.83 (t, 4H, *J* = 4.6 Hz), 3.57 (bs, 2H), 2.95 (t, 4H, *J* = 4.5 Hz). ^{13}C NMR (100 MHz, $CDCl_3$): δ 157.0 (d, *J* = 245.6 Hz), 143.0 (d, *J* = 10.6 Hz), 131.8, 120.5 (d, *J* = 4.4 Hz), 110.8 (d, *J* = 2.9 Hz), 104.1 (d, *J* = 23.8 Hz), 67.4, 52.0 (d, *J* = 2.0 Hz). HR-MALDI-MS (DHB): Calcd for $C_{10}H_{13}FN_2O+H^+$ m/z 197.10902 ($[M+H]^+$), found 197.10849.

3-Fluoro-*N*-(2,2-dimethoxyethyl)-4-(morpholine-4-yl)aniline (5)

This compound was prepared according to the procedure described in ref. [3]. To a suspension of 3-fluoro-4-(morpholine-4-yl)aniline (**3**, 1.6 g; 8.2 mmol) in dry DCM (50 mL) and 4 Å molecular sieves (0.5 g) was added a solution of dimethoxyacetaldehyde (60%) in water (1.84 mL, 12.2 mmol). The mixture was stirred at room temperature for 1 h. Then, $NaBH(OAc)_3$ (2.35 g; 10.6 mmol) was added stepwise within 20 min and the slurry was stirred at room temperature for 2 h. The mixture was filtered through a plug of Celite and washed with DCM (70 mL). The filtrate was evaporated under reduced pressure and the crude product **5** was purified by column chromatography (R_f 0.46; SiO_2, DCM/acetone 8:1 (v/v)). Yield: 1.72 g (74 %); white crystalline solid, mp 74.4–74.9 °C; ^{1}H NMR (400 MHz, $CDCl_3$): δ 6.80 (t, 1H, *J* = 9.0 Hz), 6.35 (m, 2H), 4.51 (t, 1H, *J* = 5.5 Hz), 3.82 (t, 4H, *J* = 4.60 Hz), 3.38 (s, 6H), 3.16 (d, 2H, *J* = 4.80 Hz), 2.94 (s, 4H). ^{13}C NMR (100 MHz, $CDCl_3$): δ 157.2 (d, *J* = 244.90 Hz), 144.9 (d, *J* = 9.6 Hz), 131.1 (d, *J* = 9.5 Hz), 120.5 (d, *J* = 2.9 Hz), 108.7, 102.6, 101.9 (d, *J* = 24.4 Hz), 67.3, 54.1 (d, *J* = 2.1 Hz), 52.0, 45.9. HR-MALDI-MS (DHB): Calcd for $C_{14}H_{21}FN_2O_3+H^+$ m/z 285.16145 ($[M+H]^+$), found 285.16147.

N-4-[(2,2-Dimethoxyethylamino)phenyl]morpholine-3-one (6)

Compound **6** was prepared according to the procedure described in ref. [5]. to a suspension of *N*-4-aminophenylmorpholine-3-one (**4**, 1.92 g; 10 mmol) in dry DCM (40 mL) and 4 Å molecular sieves (1.5 g) was added a solution of dimethoxyacetaldehyde (60%) in water (2.3 mL, 15 mmol). The mixture was stirred at room temperature for 2 h. Then, solid $NaBH(OAc)_3$ (3.2 g; 15 mmol) was added stepwise within 20 min and the slurry was stirred for 1 hour at room temperature. The mixture was filtered through a plug of Celite and washed with DCM (70 mL). The filtrate was evaporated under reduced pressure and the crude product **6** was purified by column chromatography (SiO_2; R_f 0.44; acetone/hexanes/TEA 2:1:0.001 (v/v)). Yield: 2.47 g (88%); white crystalline solid, mp 104.8–106.2 °C; ^{1}H NMR (500 MHz, $CDCl_3$): δ 7.10 (d, 2H, J = 8.8 Hz), 6.68 (d, 2H, J = 8.8 Hz), 4.58 (t, 1H, J = 5.5 Hz), 4.32 (s, 2H), 4.00 (t, 2H, J = 5.0 Hz), 3.69 (t, 2H, J = 5.2 Hz), 3.41 (s, 6H), 3.24 (d, 2H, J = 5.5 Hz); ^{13}C NMR ($CDCl_3$; 125 MHz): δ 167.2, 147.3, 131.6, 127.0, 113.6, 102.6, 68.9, 64.4, 54.2, 50.4, 45.6. HR-MALDI-MS (DHB): Calcd. for $C_{14}H_{20}N_2O_4+H^+$ ($[M+H]^+$) m/z 281.15014, found 281.15097.

L-Menthyl chloroformate (7)

This compound was prepared according to the procedure described in ref. [1]. To a solution of pyridine (0.44 mL) in dry toluene (5 mL) cooled in ice-bath was added dropwise a solution of triphosgene (440 mg, 1.48 mmol) in dry toluene (3 mL). Then, L-menthol (560 mg, 3.6 mmol) was added and the mixture was stirred at room temperature for 15 h. The reaction mixture was diluted with water (15 mL) and extracted with toluene (3× 15 mL). The combined extracts were washed with water (50 mL) and brine (50 mL). The organic layer was dried over $MgSO_4$ and evaporated *in vacuo*. The crude chloroformate **7** was purified by column chromatography (SiO_2; R_f 0.36; *n*-hexane/EtOAc 4:1 (v/v)). Yield: 580 mg (74%); $[\alpha_D^{20}]$ = –81.5 (*c* 1.09; THF). ^{1}H NMR (400 MHz, $CDCl_3$): δ 4.74 (td, 1H, J = 10.9; 4.5 Hz), 2.14 (m, 1H), 1.95 (spd, 1H, J = 7.0; 2.6 Hz), 1.71 (m, 2H), 1.48 (m, 2H), 1.15 (q, 1H, J = 11.7), 1.05 (qd, 1H, J = 13.2; 3.6 Hz), 0.94 (m, 6H), 0.89 (m, 1H), 0.81 (d, 3H, J = 7.0 Hz). ^{13}C NMR (100 MHz, $CDCl_3$): δ 150.2, 84.2, 47.0, 40.3, 34.0, 31.7, 26.5, 23.6, 20.7, 16.5.

(−)-Bornyl chloroformate (8)

A mixture of (−)-borneol (12 g, 77.8 mmol) and triphosgene (34 g, 0.115 mol) in dry DCM (250 mL) was cooled to 0 °C and pyridine (6.2 mL) in dry DCM (50 mL) was slowly added. The solution was warmed to room temperature and stirred for 1 h. The solvent was removed under reduced pressure and the residue was treated with Et_2O (250 mL). The suspension formed was filtered off, the solid washed with Et_2O (200 mL) and the filtrate was evaporated. The crude (−)-bornyl chloroformate can be used directly in the next reaction step. If necessary, it can be purified by column chromatography (SiO_2; R_f 0.66; *n*-hexane/EtOAc 1:2 (v/v)). Yield: 11.0 g (65%), yellow oil; $[\alpha_D^{20}]$ = –36.5 (*c* 1.02; THF). ^{1}H NMR (400 MHz, $CDCl_3$): δ 4.95–4.89 (m, 1H), 2.39–2.28 (m, 1H), 1.89–1.79 (m, 1H), 1.76–1.69 (m, 1H), 1.69–1.63 (m, 1H), 1.34–1.16 (m, 3H), 1.14–1.07 (dd, 1H, J = 14.1; 3.3 Hz), 0.83 (s, 3H), 0.82 (s, 6H). ^{13}C NMR (100 MHz, $CDCl_3$): δ 151.1, 89.7, 49.8, 48.3, 45.4, 39.3, 28.6, 26.2, 20.5, 19.0, 13.6.

O-Ethyl-*N*-[3-fluoro-4-(morpholine-4-yl)phenyl]-*N*-(2,2-dimethoxyethyl) carbamate (9)

The carbamate **9** was prepared according to the procedure described in ref. [3]. To a solution of amine **6** (285 mg; 1 mmol) and DIPEA (174 µL, 1 mmol) in dry MeCN (6 mL) was added ethyl chloroformate (121 µL, 1.25 mmol). The mixture was stirred at room temperature for 24 h. Then, the reaction mixture was diluted with water (20 mL) and extracted with EtOAc (3 ×

30 mL). The organic layers were dried over $MgSO_4$ and evaporated *in vacuo*. The crude carbamate was purified by column chromatography (SiO_2; R_f 0.47; *n*-hexane/acetone 2:1 (v/v)). Yield: 330 mg (93%); yellow oil; ^{1}H NMR (400 MHz, $CDCl_3$): δ 6.93 (m, 2H), 6.83 (t, 1H, *J* = 9.1 Hz), 4.52 (t, 1H, *J* = 5.5 Hz), 4.07 (q, 2H, *J* = 7.2 Hz), 3.82 (t, 4H, *J* = 4.7 Hz), 3.65 (d, 2H, *J* = 5.6 Hz), 3.28 (s, 6H), 3.04 (t, 4H, *J* = 4.6 Hz), 1.21 (t, 3H, *J* = 7.1 Hz). ^{13}C NMR (100 MHz, $CDCl_3$): δ 155.0 (d, *J* = 247.1 Hz), 155.7, 138.5 (d, *J* = 8.6 Hz), 137.0, 123.3, 118.4 (d, *J* = 3.1 Hz), 115.8 (d, *J* = 22.0 Hz), 101.9, 67.1, 62.0, 53.8, 51.8, 51.0 (d, *J* = 3.1 Hz), 14.7. HR-MALDI-MS (DHB): Calcd for $C_{17}H_{25}FN_2O_5+H^+$ m/z 357.18258 ($[M+H]^+$), found 357.18350.

_O_-L-Menthyl-_N_-[3-fluoro-4-(morpholine-4-yl)phenyl]-_N_-(2,2-dimethoxyethyl) carbamate (10)

The carbamate **10** was prepared according to the procedure analogical to the synthesis of **9** at a 1 mmol scale. Yield: 354 mg (76%); yellow oil, R_f 0.38 (SiO_2; *n*-hexane/EtOAc 2:1 (v/v)); $[\alpha_D^{20}]$ = –9.0 (*c* 0.60; THF). ^{1}H NMR (400 MHz, $CDCl_3$): δ 7.01–6.80 (m, 3H), 4.54 (t, 2H, *J* = 5.1 Hz), 3.84 (t, 4H, *J* = 4.6 Hz), 3.71–3.60 (m, 2H), 3.29 (s, 6H), 3.06 (t, 4H, *J* = 4.6 Hz), 2.04 (bs, 1H), 1.76 (bs, 1H), 1.60 (bs, 2H), 1.44 (bs, 1H), 1.22 (t, 1H, *J* = 7.1 Hz), 1.00 (q, 1H, *J* = 12.6 Hz), 0.92–0.67 (m, 10H). ^{13}C NMR (100 MHz, $CDCl_3$): δ 155.5, 155.1 (d, *J* = 246.9 Hz), 138.4 (d, *J* = 7.8 Hz), 137.4, 123.4, 118.4, 116.0 (d, *J* = 19.5 Hz), 102.1, 76.3, 67.1, 54.2, 53.8, 51.8, 51.1 (d, *J* = 3.0 Hz), 47.3, 41.4, 34.4, 31.6, 26.5, 23.6, 22.2, 21.2, 16.6. HR-MALDI-MS (DHB): Calcd for $C_{25}H_{39}FN_2O_5+H^+$ m/z 467.29213 ($[M+H]^+$), found 467.29236.

O-tert-Butyl-_N_-[3-fluoro-4-(morpholine-4-yl)phenyl]-_N_-(2,2-dimethoxyethyl) carbamate (11)

The carbamate **11** was prepared according to the procedure analogical to the synthesis of **9** at a 1 mmol scale. Yield: 330 mg (86%); yellow oil, R_f 0.57 (SiO_2; $CHCl_3$/acetone 10:1 (v/v)). ^{1}H NMR (400 MHz, $CDCl_3$): δ 6.96 (m, 2H), 6.84 (t, 2H, *J* = 9.2 Hz), 4.55 (t, 1H, *J* = 5.5 Hz), 3.84 (t, 4H, *J* = 4.6 Hz), 3.63 (d, 2H, *J* = 5.6 Hz), 3.30 (s, 6H), 3.05 (t, 4H, *J* = 4.6 Hz), 1.42 (s, 9H). ^{13}C NMR (100 MHz, $CDCl_3$): δ 156.2, 155.2 (d, *J* = 246.3 Hz), 138.9 (d, *J* = 8.3 Hz), 136.9 (d, *J* = 3.8 Hz), 123.0, 118.4 (d, *J* = 3.0 Hz), 115.5 (d, *J* = 22.5 Hz), 97.9, 82.1, 67.1, 53.7, 50.9, 45.8, 28.9. HR-MALDI-MS (DHB): Calcd for $C_{19}H_{29}FN_2O_5+H^+$ m/z 385.21388 ($[M+H]^+$), found 385.21380.

_O_-(−)-Bornyl-_N_-[3-fluoro-4-(morpholine-4-yl)phenyl]-_N_-(2,2-dimethoxyethyl) carbamate (12)

The carbamate **12** was prepared according to the procedure analogical to the synthesis of **9** in 20 mmol scale. Yield: 7.89 g (85%); colourless oil; R_f 0.38 (SiO_2; *n*-hexane/EtOAc 2:1 (v/v)); $[\alpha_D^{20}]$ = –14.2 (*c* 1.04; THF). ^{1}H NMR (400 MHz, $CDCl_3$): δ 7.09–6.86 (m, 3H), 4.79 (bs, 1H), 4.58 (t, 1H, *J* = 5.6 Hz), 3.86 (t, 4H, *J* = 4.5 Hz), 3.73–3.64 (m, 2H), 3.32 (s, 6H), 3.06 (t, 4H, *J* = 4.3 Hz), 2.33 (bs, 1H), 1.61 (bs, 2H), 1.28 (bs, 1H), 1.08–0.66 (m, 12H). ^{13}C NMR (100 MHz, $CDCl_3$): δ 155.1 (d, *J* = 252.3 Hz), 138.6, 137.2, 123.5, 118.3, 116.0 (d, *J* = 20.9 Hz), 102.6, 101.7, 81.8, 67.1, 53.9, 51.7, 51.1 (d, *J* = 2.9 Hz), 48.9, 47.9, 44.9, 37.2, 28.2, 27.1, 19.9, 19.0, 13.7. HR-MALDI-MS (DHB): Calcd for $C_{25}H_{37}FN_2O_5+H^+$ m/z 465.27648 ($[M+H]^+$), found 465.27647.

O-Ethyl-N-[4-(3-oxomorpholine-4-yl)phenyl]-N-(2,2-dimethoxyethyl) carbamate (13)

The carbamate **13** was prepared according to the procedure described in ref. [5]. To a solution of amine **6** (1.68 g; 6 mmol) in dry DCM (6 mL) was added ethyl chloroformate (1.72 g; 18 mmol). The mixture was stirred at room temperature for 30 min and then, dry pyridine (0.48 mL; 6 mmol) was added. The suspension formed was stirred for 2 h. The excess of chloroformate was distilled off, the residue was treated with water (20 mL) and extracted with DCM (2 × 20 mL). The organic layers were dried over $MgSO_4$ and evaporated *in vacuo*. Yield: 1.73 g (82%); yellow oil; ^{1}H NMR ($CDCl_3$; 500 MHz): δ 7.31 (m, 4H), 4.60 (t, 1H, J = 5.5 Hz), 4.34 (s, 2H), 4.17 (q, 2H, J = 6.8 Hz) 4.03 (t, 2H, J = 4.8 Hz), 3.77 (t, 2H, J = 5.0 Hz), 3.74 (d, 2H, J = 5.5 Hz), 3.33 (s, 6H), 1.24 (t, 3H, J = 6.8 Hz); ^{13}C NMR ($CDCl_3$; 125 MHz): δ 166.9, 155.9, 141.2, 139.5, 128.2, 125.8, 101.9, 68.8, 64.4, 62.1, 54.1, 51.8, 49.8, 14.7. HR-MALDI-MS (DHB): Calcd. for $C_{17}H_{24}N_2O_6$+Na^+ ([M+Na]$^+$) m/z 375.15321, found 375.15338.

O-(−)-Bornyl-N-[4-(3-oxomorpholine-4-yl)phenyl]-N-(2,2-dimethoxyethyl) carbamate (14)

The carbamate **14** was prepared according to the procedure analogical to the synthesis of **13** in 20 mmol scale. Yield: 5.97 g (65%); colourless oil; R_f 0.27 (SiO_2; n-hexane/acetone 2:1 (v/v)); [α_D^{20}] = –13.0 (c 0.67; THF). ^{1}H NMR (500 MHz, $CDCl_3$): δ 7.31 (m, 4H), 4.80 (m, 1H), 4.60 (t, 1H, J = 5.5 Hz), 4.31 (s, 2H), 4.00 (t, 3H, J = 4.0 Hz), 3.77–3.68 (m, 4H), 3.31 (s, 6H), 2.32 (m, 1H), 1.72–1.56 (m, 3H), 1.18–0.87 (m, 3H), 0.85 (s, 6H), 0.80 (s, 3H); ^{13}C NMR (125 MHz, $CDCl_3$): δ 166.7, 155.9, 140.0, 139.4, 128.1, 125.5, 101.8, 81.8, 68.7, 64.2, 53.8, 51.5, 49.6, 48.9, 47.8, 44.8, 37.1, 28.1, 27.1, 19.8, 19.0, 13.7. HR-MALDI-MS (DHB): Calcd for $C_{25}H_{36}N_2O_6$+H^+ ([M+H]$^+$) m/z 460.25734, found 460.25723.

O-Ethyl-N-[3-fluoro-4-(morpholine-4-yl)phenyl]-N-(2-oxoethyl) carbamate (15)

The aldehyde **15** was prepared according to the procedure described in ref. [3]. A solution of acetal **9** (300 mg; 0.84 mmol) in a mixture of MeCN (25 mL), HCl (36% aq.; 1.0 mL), and water (1.0 mL) was stirred at room temperature for 24 h. The mixture was neutralized with a saturated aqueous solution of $NaHCO_3$. The volume of the mixture was reduced by evaporation of solvents, the residue was diluted with water (ca 10 mL), and extracted with EtOAc (3 × 30 mL). The organic layers were dried over $MgSO_4$ and evaporated *in vacuo*. The crude product **15** was purified by column chromatography using (SiO_2; R_f 0.36; n-hexane/acetone 2:1 (v/v)). Yield: 240 mg (92%); white crystalline solid, mp 83.5–85.2 °C; ^{1}H NMR (400 MHz, $CDCl_3$): δ 9.66 (s, 1H), 6.98–6.83 (m, 3H), 4.34 (s, 2H), 4.16 (q, 2H, J = 7.1 Hz), 3.84 (t, 4H, J = 4.7 Hz), 3.05 (t, 4H, J = 4.7 Hz), 1.20 (t, 3H, J = 6.9 Hz). ^{13}C NMR (100 MHz, $CDCl_3$): δ 197.4, 155.6, 155.0 (d, J = 247.8 Hz), 138.9 (d, J = 8.6 Hz), 122.7, 118.6, 115.4, 67.0, 62.6, 60.4, 50.9 (d, J = 3.7 Hz), 14.5. HR-MALDI-MS (DHB): Calcd for $C_{15}H_{19}FN_2O_4$+H^+ m/z 311.14072 ([M+H]$^+$), found 311.14066.

O-L-Menthyl-N-[3-fluoro-4-(morpholine-4-yl)phenyl]-N-(2-oxoethyl) carbamate (16)

The aldehyde **16** was prepared according to the procedure analogical to the synthesis of **15** at a 0.67 mmol scale. Yield: 264 mg (94%); colourless oil; R_f 0.22 (SiO_2; n-hexane/EtOAc 2:1 (v/v)); [α_D^{20}] = –31.2 (c 0.44; THF). ^{1}H NMR (400 MHz, $CDCl_3$): δ 9.64 (s, 1H), 7.06–6.79 (m, 3H), 4.58 (t, 1H, J = 9.9 Hz), 4.31 (s, 2H), 3.83 (t, 4H, J = 4.9 Hz), 3.04 (t, 4H, J = 4.6 Hz), 2.02 (m, 1H), 1.78 (bs, 1H), 1.61 (d, 2H, J = 9.4 Hz), 1.43 (bs, 1H), 1.22 (t, 1H, J = 7.4 Hz), 1.00 (qd, 1H, J = 9.9, 2.76 Hz), 0.92–0.69 (m, 11H). ^{13}C NMR (100 MHz, $CDCl_3$): δ 197.5, 155.3, 155.1 (d, J = 247.7 Hz), 138.8 (J = 6.1 Hz), 136.8, 122.9, 118.6 (d, J = 2.0 Hz), 115.5,

76.9, 67.1, 60.4, 51.0 (d, J = 3.3 Hz), 47.1, 41.2, 34.3, 31.5, 26.5, 23.6, 22.2, 20.9, 16.5. HR-MALDI-MS (DHB): Calcd for $C_{23}H_{33}FN_2O_4$+H^+ m/z 421.25027 ($[M+H]^+$), found 421.25027.

Methyl *N*-[3-fluoro-4-(morpholine-4-yl)phenyl]aminoacetate (Int-17a)

The compound **Int-17a** was prepared according to the modified method described in ref. [4]. A mixture of amine **3** (392 mg, 2 mmol), dry K_2CO_3 (553 mg, 4 mmol), methyl bromoacetate (200 µL, 2.44 mmol), and TBAI (10 mg) was stirred in extra dry DMF (10 mL) at 120 °C for 8 h. After cooling, the solvent was evaporated under reduced pressure and the residue was chromatographed (SiO_2; EtOAc/*n*-hexane 10:1 to 1:1 (v/v)). The product was recrystallized form a mixture of EtOAc/*n*-hexane 1:5 (v/v). Yield: 490 mg (92%), pale yellow solid, mp 110.5–110.9 °C. R_f 0.32 (SiO_2; *n*-hexane/EtOAc 3:1 (v/v)). ^{1}H NMR (400 MHz, $CDCl_3$): δ 6.85 (m, 1H), 6.34 (m, 2H), 4.18 (bs, 1H), 3.84 (m, 6H), 3.76 (s, 3H), 2.96 (m, 4H). ^{13}C NMR (100 MHz, $CDCl_3$): δ 171.6, 157.1 (d, J = 245.2 Hz), 143.9 (d, J = 10.0 Hz), 131.6 (d, J = 8.5 Hz), 120.5 (d, J = 3.6 Hz), 108.6, 102.0 (d, J = 24.7 Hz), 67.3, 52.5, 51.9, 46,1. HR-MALDI-MS (DHB): Calcd for $C_{13}H_{17}FN_2O_3$+H^+ m/z 269.13015 ($[M+H]^+$), found 269.13007.

Methyl *N*-(*tert*-butoxycarbonyl)-*N*-[3-fluoro-4-(morpholine-4-yl)phenyl]aminoacetate (Int-17b)

The compound **Int-17b** was prepared according to the modified method described in ref. [4]. A solution of ester **Int-17a** (800 mg, 3 mmol), Boc_2O (786 mg, 3.6 mmol), and DMAP (15 mg) in extra dry DMF (28 mL) was heated at 130 °C for 1 h. After cooling, Et_2O (50 mL) was added and the solution was washed with brine (50 mL). The organic layer was dried with Na_2SO_4 and the solvents were evaporated under reduced pressure. The residue was chromatographed (SiO_2; EtOAc/*n*-hexane 1:10 to 1:1 (v/v)). Yield: 460 mg (42%), yellow oil; R_f 0.20 (SiO_2; *n*-hexane/EtOAc 3:1 (v/v)). ^{1}H NMR (400 MHz, $CDCl_3$): δ 7.14–6.78 (m, 3H), 4.21 (s, 2H), 3.83 (t, 4H, J = 4.6 Hz), 3.73 (s, 3H), 3.04 (t, 4H, J = 4.6 Hz), 1.40 (s, 9H). ^{13}C NMR (100 MHz, $CDCl_3$): δ 170.4, 155.0 (d, J = 247.9 Hz), 154.4, 138.5 (d, J = 8.6 Hz), 137.4 (d, J = 9.5 Hz), 122.5, 118.3, 115.2, 81.4, 67.0, 52.3 (d, J = 2.8 Hz), 51.9, 51.0 (d, J = 2.9 Hz), 28.3. HR-MALDI-MS (DHB): Calcd for $C_{18}H_{25}FN_2O_5$+H^+ m/z 369.18258 ($[M+H]^+$), found 369.18256.

***O*-*tert*-Butyl-*N*-[3-fluoro-4-(morpholine-4-yl)phenyl]-*N*-(2-oxoethyl) carbamate (17)**

The aldehyde **17** was prepared according to the modified method described in ref. [4]. A solution of acetate **Int-17b** (410 mg, 1.1 mmol) in dry toluene (6 mL) under argon atmosphere was cooled to −78 °C and a 1 M solution of DIBAL-H in toluene (1.7 mL, 1.7 mmol) was slowly added. The mixture was stirred at −78 °C for 1 h and then, the reaction was quenched with the addition of methanol (0.9 mL). The mixture was warmed to room temperature and the solution was washed with saturated aqueous solution of NH_4Cl (20 mL). The organic layer was separated and the aqueous layer was extracted with EtOAc (3 × 20 mL). The combined organic layers were dried with Na_2SO_4 and the solvents were evaporated under reduced pressure. The residue was chromatographed (SiO_2; EtOAc/*n*-hexane 1:10 to 1:1 (v/v)). Yield: 332 mg (89%), yellowish oil; R_f 0.32 (SiO_2; *n*-hexane/EtOAc 1:3 (v/v)). ^{1}H NMR (400 MHz, $CDCl_3$): δ 9.66 (s, 1H), 7.02–6.81 (m, 3H), 4.28 (s, 2H), 3.84 (t, 4H, J = 4.6 Hz), 3.04 (t, 4H, J = 4.6 Hz), 1.41 (s, 9H). ^{13}C NMR (100 MHz, $CDCl_3$): δ 197.7, 155.0 (d, J = 245.5 Hz), 154.3, 138.4 (d, J = 8.5 Hz), 137.1 (d, J = 9.5 Hz), 122.2, 118.4, 115.1 (d, J = 20.6 Hz), 81.8, 66.9, 60.2, 50.9 (d, J = 3.1 Hz), 28.2. HR-MALDI-MS (DHB): Calcd for $C_{17}H_{23}FN_2O_4$+H^+ m/z 339.17202 ($[M+H]^+$), found 339.17233.

O-(−)-Bornyl-N-[3-fluoro-4-(morpholine-4-yl)phenyl]-N-(2-oxoethyl) carbamate (18)

The aldehyde **18** was prepared according to the procedure analogical to the synthesis of **15** at a 12.5 mmol scale. Yield: 4.91 g (94%), colourless oil; R_f 0.23 (SiO_2; *n*-hexane/EtOAc 2:1 (v/v); $[\alpha_D^{20}]$ = –27.5 (*c* 0.98; THF). ^1H NMR (400 MHz, $CDCl_3$): δ 9.64 (s, 1H), 7.02–6.83 (m, 3H), 4.79 (bs, 1H), 4.34 (s, 2H), 3.81 (t, 4H, *J* = 4.6 Hz), 3.03 (m, 4H), 2.30 (m, 1H), 1.61 (bs, 2H), 1.34 (bs, 1H), 1.18–0.93 (m, 3H), 0.87–0.67 (m, 9H). ^{13}C NMR (100 MHz, $CDCl_3$): δ 197.4, 155.4, 155.0 (d, *J* = 248.3 Hz), 138.8, 136.6 (d, *J* = 52.6 Hz), 122.5 (d, *J* = 122.7 Hz), 118.4, 115.1 (d, *J* = 74.9 Hz), 82.4, 67.0, 60.3, 50.9 (d, *J* = 3.1 Hz), 48.9, 47.8, 44.8, 36.9, 28.0, 27.0, 19.7, 18.9, 13.6. HR-MALDI-MS (DHB): Calcd for $C_{23}H_{31}FN_2O_4$+H^+ m/z 419.23462 ($[M+H]^+$), found 419.23454.

O-Ethyl-N-[4-(3-oxomorpholine-4-yl)phenyl]-N-(2-oxoethyl) carbamate (19)

The aldehyde **19** was prepared according to the procedure described in ref. [5]. A solution of acetal **13** (1.42 g; 4.2 mmol) in a mixture of MeCN (30 mL), HCl (36% aq.; 1.0 mL), and water (1.0 mL) was stirred at room temperature for 24 h. The mixture was neutralized with saturated aqueous solution of $NaHCO_3$. The volume of mixture was reduced by evaporation of solvents, the residue was diluted with water (ca 20 mL) and extracted with DCM (3 × 40 mL). The organic layers were dried over $MgSO_4$ and evaporated *in vacuo*. The crude product **19** was purified by column chromatography (R_f 0.55; SiO_2; acetone/hexanes 1:1 (v/v)). Yield: 1.29 g (93%); white crystalline solid, mp 141.2–143.7 °C; ^1H NMR (500 MHz, $CDCl_3$): δ 9.69 (s, 1H), 7.32 (m, 4H), 4.41 (s, 2H), 4.34 (s, 2H), 4.19 (q, 2H, *J* = 7.0 Hz), 4.03 (t, 2H, *J* = 4.9 Hz), 3.77 (t, 2H, *J* = 5.0 Hz), 1.23 (t, 3H, *J* = 7.1 Hz); ^{13}C NMR ($CDCl_3$; 125 MHz): δ 197.3, 167.0, 155.5, 140.9, 139.8, 127.5, 126.3, 68.7, 64.4, 62.7, 60.5, 49.7, 14.8. HR-MALDI-MS (DHB): Calcd. for $C_{15}H_{18}N_2O_5$+H^+ ($[M+H]^+$) m/z 307.12940, found 307.12977.

O-(−)-Bornyl-N-[4-(3-oxomorpholine-4-yl)phenyl]-N-(2-oxoethyl) carbamate (20)

The aldehyde **20** was prepared according to the procedure analogical to the synthesis of **19** at a 12.5 mmol scale. Yield: 4.85 (94%); white crystalline solid, mp 68.3–71.1 °C; R_f 0.16 (SiO_2; *n*-hexane/acetone 2:1 (v/v)); $[\alpha_D^{20}]$ = –11.2 (*c* 0.12; THF). ^1H NMR (500 MHz, $CDCl_3$): δ 9.71 (s, 1H), 7.32 (m, 4H), 4.85 (m, 1H), 4.41 (s, 2H), 4.33 (s, 2H), 4.03 (t, 3H, *J* = 5.0 Hz), 3.76 (t, 3H, *J* = 5.0 Hz), 2.35 (m, 1H), 1.76–1.57 (m, 3H), 1.21–0.93 (m, 3H), 0.88 (s, 3H), 0.83 (s, 3H), 0.80 (s, 3H); ^{13}C NMR (125 MHz, $CDCl_3$): δ 197.4, 166.9, 155.8, 140.7, 139.8, 127.6, 126.0, 82.6, 68.7, 64.3, 60.3, 49.7, 49.0, 47.9, 44.9, 37.0, 28.1, 27.3, 19.8, 19.0, 13.7. HR-MALDI-MS (DHB): Calcd. for $C_{23}H_{30}N_2O_5$+H^+ ($[M+H]^+$) m/z 414.21547, found 414.21600.

General procedure for asymmetric Henry reaction of aldehydes 15–20 with nitromethane

A mixture of chiral ligand **I–VIII** (27.5 μmol; 11 mol %) or (13.8 μmol; 5.5 mol %) and $Cu(OAc)_2$ (4.52 mg, 25 μmol; 10 mol %) or (2.27 mg, 12.5 μmol; 5 mol %) in dry IPA (1 mL) and nitromethane (0.27 mL; 5 mmol) was stirred for 1 h. The clear blue or green solution was cooled to 6 °C or 20 °C and the aldehyde **15–21** (0.25 mmol) was added. The mixture was stirred at the corresponding temperature for 7 d. Afterwards, the reaction mixture was flash-chromatographed (SiO_2; EtOAc; ca. 50 mL). The solvents were evaporated under reduced pressure and the residue was analyzed by ^{1}H NMR (for determination of conversion). The nitroaldol was separated by column chromatography.

***O*-Ethyl-*N*-[3-fluoro-4-(morpholine-4-yl)phenyl]-*N*-(2-hydroxy-3-nitroprop-1-yl) carbamate (21)**

White crystalline solid, mp 113.1–114.6 °C; R_f 0.28 (SiO_2; *n*-hexane/acetone 2:1 (v/v); $[\alpha_D^{20}]$ = 15.3 (*c* 0.20; THF, for 89% ee of using the catalyst **IV**). ^{1}H NMR (500 MHz, $CDCl_3$): δ 6.95–6.87 (m, 3H), 4.51 (m, 1H), 4.47–4.40 (m, 2H), 4.14 (m, 2H), 3.86–3.71 (m, 6H), 3.08 (d, 4H, *J* = 4.5 Hz), 1.19 (m, 3H). ^{13}C NMR (125 MHz, $CDCl_3$): δ 157.1, 155.1 (d, *J* = 247.5 Hz), 139.2 (d, *J* = 8.8 Hz), 136.1 (d, *J* = 8.8 Hz), 123.2, 118.7 (d, *J* = 3.7 Hz), 115.6 (d, *J* = 21.3 Hz), 78.8, 68.0, 67.1, 62.8, 53.9, 50.8 (d, *J* = 3.7 Hz), 14.6. HR-MALDI-MS (DHB): Calcd for $C_{16}H_{22}FN_3O_6+H^+$ m/z 372.15709 ($[M+H]^+$), found 372.15717. Enantiomeric excess was determined by HPLC – Chiralpak AS-H; *n*-hexane/IPA (v/v; 70/30); flow rate 1.0 mL/min; λ = 254 nm: *S*-enantiomer t_R = 12.42 min; *R*-enantiomer t_R = 15.21 min.

***O*-L-Menthyl-*N*-[3-fluoro-4-(morpholine-4-yl)phenyl]-*N*-(2-hydroxy-3-nitroprop-1-yl) carbamate (22)**

Colourless oil; R_f 0.34 (SiO_2; *n*-hexane/acetone 3:1 (v/v); $[\alpha_D^{20}]$ = –26.2 (*c* 0.206; DCM, 89% *de* of using the catalyst **IV**). ^{1}H NMR (500 MHz, $CDCl_3$): δ 6.90 (m, 3H), 4.49 (m, 4H), 3.79 (m, 6H), 3.10 (t, 4H, *J* = 4.4 Hz), 2.02 (d, 1H, *J* = 11.3 Hz), 1.66 (m, 4H), 1.46 (m, 1H), 1.21 (m, 1H), 1.01 (q, 1H, *J* = 12.7 Hz), 0.87 (m, 8H), 0.75 (m, 3H). ^{13}C NMR (125 MHz, $CDCl_3$): δ 156.9, 155.0 (d, *J* = 248.5 Hz), 139.1 (d, *J* = 8.4 Hz), 136.1 (d, *J* = 9.5 Hz), 123.2, 118.5 (d, *J* = 4.0 Hz), 115.6 (d, *J* = 22.1 Hz), 78.7, 68.2, 66.9, 53.9, 50.8 (d, *J* = 3.7 Hz), 47.0, 41.0, 34.1, 31.4, 26.9, 26.4, 23.4, 22.0, 20.7, 16.3. HR-MALDI-MS (DHB): Calcd for $C_{24}H_{36}FN_3O_6+H^+$ m/z 482.26665 ($[M+H]^+$), found 482.26718. Diastereomeric excess was determined by HPLC – Chiralpak AD-H; *n*-hexane/IPA (v/v; 85/15); flow rate 1.0 mL/min; λ = 280 nm: (2*R*)-stereoisomer t_R = 20.93 min; (2*S*)-stereoisomer t_R = 25.12 min.

***O*-*tert*-Butyl-*N*-[3-fluoro-4-(morpholine-4-yl)phenyl]-*N*-(2-hydroxy-3-nitroprop-1-yl) carbamate (23)**

Colourless oil; R_f 0.22 (SiO_2; *n*-hexane/EtOAc 3:1 (v/v); $[\alpha_D^{20}]$ = 83.3 (*c* 0.39; DCM, 87 % *ee* of using the catalyst **IV**). ^{1}H NMR (400 MHz, $CDCl_3$): δ 6.96 (m, 3H), 4.53 (m, 3H), 3.93 (t, 4H, *J* = 4.5 Hz), 3.82 (ddd, 2H, *J* = 21.9; 14.6; 4.5 Hz), 3.15 (t, 4H, *J* = 4.5 Hz), 1.47 (s, 9H). ^{13}C NMR (100 MHz, $CDCl_3$): δ 156.2, 155.2 (d, *J* = 247.8 Hz), 138.9 (d, *J* = 8.4 Hz), 136.8 (d, *J* = 9.6 Hz), 123.0 (d, *J* = 3.0 Hz), 118.6 (d, *J* = 3.8 Hz), 115.5 (d, *J* = 22.3 Hz), 82.1, 78.9, 68.4, 67.1, 53.7, 51.0 (d, *J* = 3.1 Hz), 28.4. HR-MALDI-MS (DHB): Calcd for $C_{18}H_{26}FN_3O_6+H^+$ m/z 400.18839 ($[M+H]^+$), found 400.18848. Enantiomeric excess was determined by HPLC – Chiralpak AS-H, *n*-hexane/IPA (v/v; 85/15); flow rate 1.0 mL/min; λ = 285 nm: *S*-enantiomer t_R = 14.73 min; *R*-enantiomer t_R = 20.64 min.

O-(−)-Bornyl-N-[3-fluoro-4-(morpholine-4-yl)phenyl]-N-(2-hydroxy-3-nitroprop-1-yl) carbamate (24)

Colourless oil; R_f 0.24 (SiO_2; $CHCl_3$/acetone 10:1 (v/v); $[\alpha_D^{20}]$ = –11.4 (*c* 0.66; DCM, 90 % *de* of using the catalyst **IV**). ^1H NMR (500 MHz, $CDCl_3$): δ 6.92 (m, 2H), 6.59 (t, 1H, *J* = 9.0 Hz), 5.14 (d, 1H, *J* = 9.4 Hz), 4.46 (m, 1H), 4.00 (m, 1H), 3.84 (dd, 1H, *J* = 13.0; 3.2 Hz), 3.66 (t, 4H, *J* = 4.6 Hz), 3.53 (dd, 1H, *J* = 14.3; 7.8 Hz), 3.40 (dd, 1H, *J* = 14.5; 4.1 Hz), 2.81 (t, 4H, *J* = 4.5 Hz), 2.50 (m, 1H), 1.66 (bs, 2H), 1.55 (s, 1H), 1.18 (m, 3H), 0.97–0.7 (m, 10H). ^{13}C NMR (125 MHz, $CDCl_3$): δ 157.5, 155.2 (d, *J* = 248.1 Hz), 139.2 (d, *J* = 7.9 Hz), 136.3 (d, *J* = 9.5 Hz), 123.4, 118.7 (d, *J* = 3.8 Hz), 115.7 (d, *J* = 22.1 Hz), 82.7, 78.9, 68.2, 67.0, 53.9, 50.9 (d, *J* = 3.2 Hz), 49.0 (d, *J* = 4.2 Hz), 47.9, 44.9, 37.1, 28.1, 27.1, 19.8, 19.0, 13.7. HR-MALDI-MS (DHB): Calcd for $C_{24}H_{34}FN_3O_6+H^+$ *m/z* 480.25099 ($[M+H]^+$), found 480.25091. Diastereomeric excess was determined by HPLC – Chiralcel OD-H, *n*-hexane/IPA (v/v; 85/15); flow rate 1.0 mL/min; λ = 254 nm: (2*S*)-stereoisomer t_r = 12.82 min; (2*R*)-stereoisomer t_r = 17.54 min.

O-Ethyl-N-[4-(3-oxomorpholine-4-yl)phenyl]-N-(2-hydroxy-3-nitroprop-1-yl) carbamate (25)

Yellow oil; R_f 0.60 (SiO_2; THF/Et_2O 2:1 (v/v)) $[\alpha]_D^{20}$ = –6.2 (*c* 0.80; DCM, 90 % *de* of using the catalyst **IV**). ^1H NMR (500 MHz, $CDCl_3$): δ 7.33 (m, 2H), 7.26 (m, 2H), 4.53–4.40 (m, 3H), 4.32 (s, 2H), 4.14 (q, 2H, *J* = 6.9 Hz) 4.04 (m, 2H), 3.92 (brs, 1H), 3.83–3.75 (m, 4H), 1,22 (t, 3H, *J* = 6,8 Hz); ^{13}C NMR ($CDCl_3$; 125 MHz): δ 167.2, 156.9, 140.6, 140.0, 128.2, 126.3, 79.0, 68.8, 68.1, 64.3, 62.9, 53.9, 49.7, 14.9. HR-MALDI-MS (DHB): Calcd. for $C_{16}H_{21}N_3O_7+H^+$ ($[M+H]^+$) m/z 368.14578, found 368.14656. Enantiomeric excess was determined by HPLC – Chiralpak AS-H, *n*-hexane/IPA (v/v; 50/50); flow rate 1.0 mL/min; λ = 240 nm: *S*-enantiomer t_r = 24.2 min; *R*-enantiomer t_r = 31.7 min.

O-(−)-Bornyl-N-[4-(3-oxomorpholine-4-yl)phenyl]-N-(2-hydroxy-3-nitroprop-1-yl) carbamate (26)

Pale yellow solid, mp 58.6–61.2 °C; R_f 0.13 (SiO_2; $CHCl_3$/acetone 5:1 (v/v)); $[\alpha_D^{20}]$ = –15.8 (*c* 0.72; DCM, 91 % *de* of using the catalyst **IV**). ^1H NMR (500 MHz, $CDCl_3$): δ 7.36 (d, 2H, *J* = 8.6 Hz), 7.28 (d, 2H, *J* = 6.9 Hz), 4.80 (m, 1H), 4.55–4.38 (m, 3H), 4.33 (s, 2H), 4.05 (t, 3H, *J* = 5.0 Hz), 3.87–3.73 (m, 4H), 2.34 (m, 1H), 1.74–1.58 (m, 3H), 1.17–0.92 (m, 3H), 0.88 (s, 3H), 0.82 (s, 3H), 0.77 (s, 3H); ^{13}C NMR (125 MHz, $CDCl_3$): δ 167.1, 157.2, 140.5, 139.9, 128.1, 126.0, 82.6, 79.0, 68.6, 68.1, 64.2, 53.6, 49.6, 49.0, 47.9, 44.8, 37.1, 28.1, 27.1, 19.8, 19.0, 13.7. HR-MALDI-MS (DHB): Calcd. for $C_{24}H_{33}N_3O_7+H^+$ ($[M+H]^+$) *m/z* 475.23185, found 475.23257. Diastereomeric excess was determined by HPLC – Chiralcel OD-H, *n*-hexane/IPA (v/v; 70/30); flow rate 1.0 mL/min; λ = 254 nm; (2*S*)-stereoisomer t_r = 11.2 min; (2*R*)-stereoisomer t_r = 14.0 min.

O-(−)-Bornyl-N-[3-fluoro-4-(morpholine-4-yl)phenyl]-N-(2-hydroxy-3-acetamidoprop-1-yl) carbamate (27)

The compound **27** was prepared according to the modified method described in ref. [3]. A mixture of nitroaldol **24** from scale up synthesis (4.0 g, 8.35 mmol) and Pd/C (10 wt %, 250 mg) in methanol (40 mL) was stirred under a hydrogen atmosphere (ca. 20 bar, pressure vessel) at room temperature for 24 h. The reaction mixture was filtered off and the catalyst was washed with MeOH (ca. 100 mL). The solvent was evaporated under reduced pressure and the residue (3.12 g, 7 mmol) was used in the next reaction step without further purification. The yellow oil

was dissolved in dry DCM (50 mL), then TEA (1.10 mL, 8 mmol) and subsequently acetic anhydride (650 µL, 7.1 mmol) were added. The reaction mixture was stirred at room temperature for 24 h, washed with saturated aqueous solution of $NaHCO_3$ and dried with Na_2SO_4. The solvent was evaporated under reduced pressure and the residue was purified by column chromatography. Yield: 2.66 g (78%); white crystalline solid, mp 54.8–56.2 °C; R_f 0.15 (SiO_2; $CHCl_3$/MeOH 20:1 (v/v)); $[\alpha_D^{20}]$ = –28.6 (*c* 1.04; DCM, 84 % *de*). ^{1}H NMR (500 MHz, $CDCl_3$): δ 6.99 (m, 2H), 6.54 (t, 1H, *J* = 9.0 Hz), 5.05 (d, 1H, *J* = 8.8 Hz), 3.99 (m, 1H), 3.78 (ddd, 2H, *J* = 44.2; 14.2; 5.6 Hz), 3.58 (m, 5H), 3.21 (m, 1H), 2.74 (m, 4H), 2.41 (m, 1H), 1.67 (s, 3H), 1.56 (bs, 1H), 1.47 (m, 1H), 1.10 (m, 3H), 0.93–0.62 (m, 10H). ^{13}C NMR (125 MHz, $CDCl_3$): δ 171.7, 157.3, 155.0 (d, *J* = 247.6 Hz), 138.8 (d, *J* = 7.3 Hz), 136.5, 123.4, 118.4 (d, *J* = 3.5 Hz), 115.7 (d, *J* = 21.8 Hz), 82.1, 69.5, 66.9, 53.6, 53.5, 50.8 (d, *J* = 2.6 Hz), 48.8, 47.7, 44.7, 43.1, 37.0, 27.9, 23.1, 19.6, 18.9, 13.6. HR-MALDI-MS (DHB): Calcd for $C_{26}H_{38}FN_3O_5+H^+$ m/z 492.28738 ($[M+H]^+$), found 492.28741. Diastereomeric excess was determined by HPLC – Chiralcel OD-H, *n*-hexane/IPA (v/v; 90/10); flow rate 1.0 mL/min; λ = 254 nm: (2*R*)-stereoisomer t_r = 14.49 min; (2*S*)-stereoisomer t_r = 19.86 min.

N-/3-{*N*-[4-(3-Oxomorpholine-4-yl)phenyl]-*N*-((−)-bornylcarbonyl)amino}-2-hydroxyprop-1-yl/-5-chlorothiophene-2-carboxamide (28)

The compound **28** was prepared according to the modified method described in ref. [5]. A mixture of nitroaldol **26** from scale up synthesis (3.79 g, 8.0 mmol) and Pd/C (10% wt, 200 mg) in MeOH (40 mL) was stirred under hydrogen atmosphere (ca. 20 bar, pressure vessel) at room temperature for 24 h. The reaction mixture was filtered off and the catalyst was washed with MeOH (ca. 100 mL). The solvent was evaporated under reduced pressure and the residue was used in the next reaction step without further purification. The yellow oil was dissolved in dry DCM (50 mL), then TEA (1.23 mL, 8.8 mmol) and subsequently 5-chlorothiophene-2-carbonylchloride (1.52 g, 8.4 mmol) was added. The reaction mixture was stirred at room temperature for 3 h. The solvent was evaporated under reduced pressure and the residue was purified by column chromatography. Yield: 4.71 g (65%); white crystalline solid, mp 82.4–84.0 °C; R_f 0.50 (SiO_2; acetone/DCM 1:1 (v/v)); $[\alpha_D^{20}]$ = –19.6 (*c* 0.5; DCM; 88% *de*). ^{1}H NMR (500 MHz, $CDCl_3$): δ 7.31 (m, 4H), 7.24 (m, 2H), 6.85 (d, 1H, *J* = 2.5 Hz), 4.76 (m, 1H), 4.31 (s, 2H), 4.10–3.98 (m, 3H), 3.88 (m, 1H), 3.80–3.57 (m, 5H), 3.26 (m, 1H), 2.30 (m, 1H), 1.68–1.53 (m, 3H), 1.13–0.88 (m, 3H), 0.84 (s, 3H), 0.78 (s, 3H), 0.72 (s, 3H); ^{13}C NMR (125 MHz, $CDCl_3$): δ 211.1, 207.5, 167.0, 162.2, 157.3, 140.6, 139.8, 137.5, 135.6, 128.2, 127.8, 127.2, 125.9, 82.3, 68.6, 64.2, 53.5, 49.6, 48.9, 47.8, 44.8, 43.3, 37.1, 31.1, 28.0, 27.0, 19.7, 19.0, 13.7. HR-MALDI-MS (DHB): Calcd. for $C_{29}H_{36}ClN_3O_6S+H^+$ ($[M+H]^+$) m/z 589.20133, found 589.20138. Diastereomeric excess was determined by HPLC – Chiralcel OD-H, *n*-hexane/IPA (v/v; 70/30); flow rate 1.0 mL/min; λ = 254 nm: (2*R*)-stereoisomer t_r = 7.86 min; (2*S*)-stereoisomer t_r = 9.91 min.

Scale up experiments of the preparation of nitroaldols 24 and 26

A mixture of chiral ligand **IV** (394 mg, 1.1 mmol; 11 mol %) and $Cu(OAc)_2$ (181 mg, 1.0 mmol; 10 mol %) in dry IPA (40 mL) and nitromethane (10 mL; 5 mmol) was stirred for 1 h at 20 °C until a clear blue solution was formed. The aldehyde **18** or **20** (10 mmol) was added and the mixture was stirred for 7 d at 20 °C. Afterwards, the reaction mixture was flash-chromatographed (SiO_2; EtOAc; ca. 500 mL). The solvents were evaporated under reduced pressure and the crude product was purified by column chromatography.

$\boldsymbol{O}$-(−)-Bornyl-$\boldsymbol{N}$-[3-fluoro-4-(morpholine-4-yl)phenyl]-$\boldsymbol{N}$-(2-hydroxy-3-nitroprop-1-yl) carbamate (24)

Yield: 4.41 g (92%); colourless oil; R_f 0.23 (SiO_2; $CHCl_3$/acetone 10:1 (v/v); $[\alpha_D^{20}]$ = –10.9 (*c* 0.62; DCM, 84 % *de*). ^{1}H NMR (500 MHz, $CDCl_3$): δ 6.93 (m, 2H), 6.59 (t, 1H, *J* = 9.0 Hz), 5.15 (d, 1H, *J* = 9.2 Hz), 4.46 (m, 1H), 4.00 (m, 1H), 3.85 (dd, 1H, *J* = 13.0; 3.2 Hz), 3.66 (t, 4H, *J* = 4.6 Hz), 3.54 (dd, 1H, *J* = 14.5; 7.8 Hz), 3.40 (dd, 1H, *J* = 14.5; 4.1 Hz), 2.81 (t, 4H, *J* = 4.5 Hz), 2.50 (m, 1H), 1.66 (bs, 2H), 1.55 (s, 1H), 1.18 (m, 3H), 0.97–0.7 (m, 10H). ^{13}C NMR (125 MHz, $CDCl_3$): δ 157.5, 155.2 (d, *J* = 248.0 Hz), 139.2 (d, *J* = 7.9 Hz), 136.3 (d, *J* = 9.5 Hz), 123.4, 118.7 (d, *J* = 3.8 Hz), 115.7 (d, *J* = 22.0 Hz), 82.7, 78.9, 68.2, 67.0, 53.9, 50.9 (d, *J* = 3.2 Hz), 49.0 (d, *J* = 4.2 Hz), 47.9, 44.9, 37.1, 28.1, 27.1, 19.8, 19.0, 13.7. HR-MALDI-MS (DHB): Calcd for $C_{24}H_{34}FN_3O_6+H^+$ m/*z* 480.25099 ($[M+H]^+$), found 480.25104. Diastereomeric excess was determined by HPLC – Chiralcel OD-H, *n*-hexane/IPA (v/v; 85/15); flow rate 1.0 mL/min; λ = 254 nm: (2*S*)-stereoisomer t_r = 12.85 min; (2*R*)-stereoisomer t_r = 17.61 min.

$\boldsymbol{O}$-(−)-Bornyl-$\boldsymbol{N}$-[4-(3-oxomorpholine-4-yl)phenyl]-$\boldsymbol{N}$-(2-hydroxy-3-nitroprop-1-yl) carbamate (26)

Yield: 3.98 g (84%); yellowish oil; R_f 0.12 (SiO_2; $CHCl_3$/acetone 5:1 (v/v)); $[\alpha_D^{20}]$ = –15.6 (*c* 0.60; DCM, 88 % *de*). ^{1}H NMR (500 MHz, $CDCl_3$): δ 7.36 (d, 2H, *J* = 8.6 Hz), 7.28 (d, 2H, *J* = 7.2 Hz), 4.80 (m, 1H), 4.52–4.36 (m, 3H), 4.33 (s, 2H), 4.06 (t, 3H, *J* = 5.0 Hz), 3.87–3.73 (m, 4H), 2.34 (m, 1H), 1.74–1.58 (m, 3H), 1.17–0.92 (m, 3H), 0.88 (s, 3H), 0.82 (s, 3H), 0.77 (s, 3H); ^{13}C NMR (125 MHz, $CDCl_3$): δ 167.1, 157.2, 140.5, 139.9, 128.1, 126.0, 82.6, 79.0, 68.6, 68.1, 64.2, 53.6, 49.6, 49.0, 47.9, 44.8, 37.1, 28.1, 27.1, 19.8, 19.0, 13.7. HR-MALDI-MS (DHB): Calcd. for $C_{24}H_{33}N_3O_7+H^+$ ($[M+H]^+$) m/*z* 475.23185, found 475.23196. Diastereomeric excess was determined by HPLC – Chiralcel OD-H, *n*-hexane/IPA (v/v; 70/30); flow rate 1.0 mL/min; λ = 254 nm; (2*S*)-stereoisomer t_r = 11.3 min; (2*R*)-stereoisomer t_r = 14.2 min.

2. NMR spectra

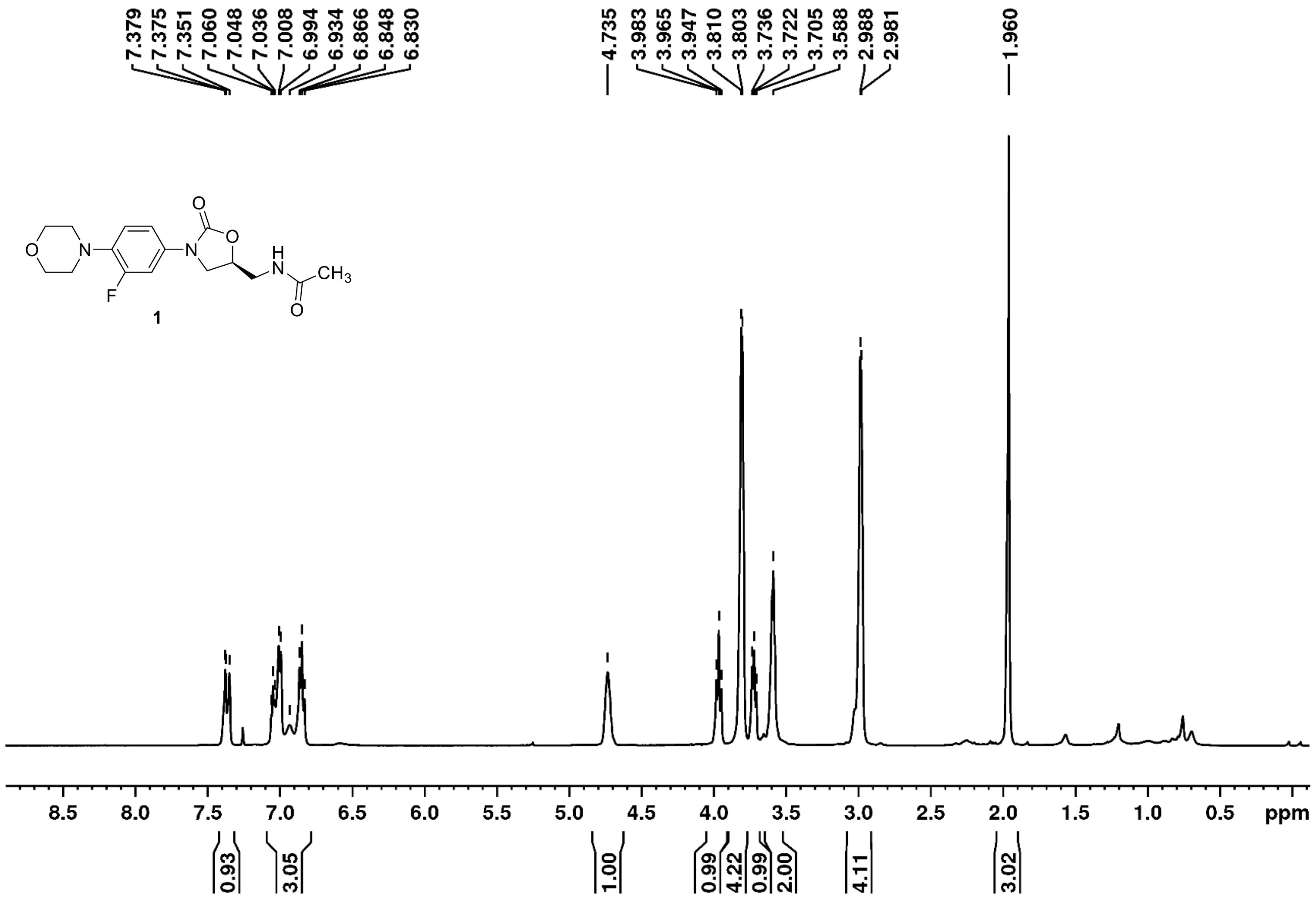
7.379
7.375
7.351
7.060
7.048
7.036
7.008
6.994
6.934
6.866
6.848
6.830
4.735
3.983
3.965
3.947
3.810
3.803
3.736
3.722
3.705
3.588
2.988
2.981
1.960
O
N
N
O
O
H
N
CH3
F
O
1
8.5
8.0
7.5
7.0
6.5
6.0
5.5
5.0
4.5
4.0
3.5
3.0
2.5
2.0
1.5
1.0
0.5
ppm
0.93
3.05
1.00
0.99
4.22
0.99
2.00
4.11
3.02

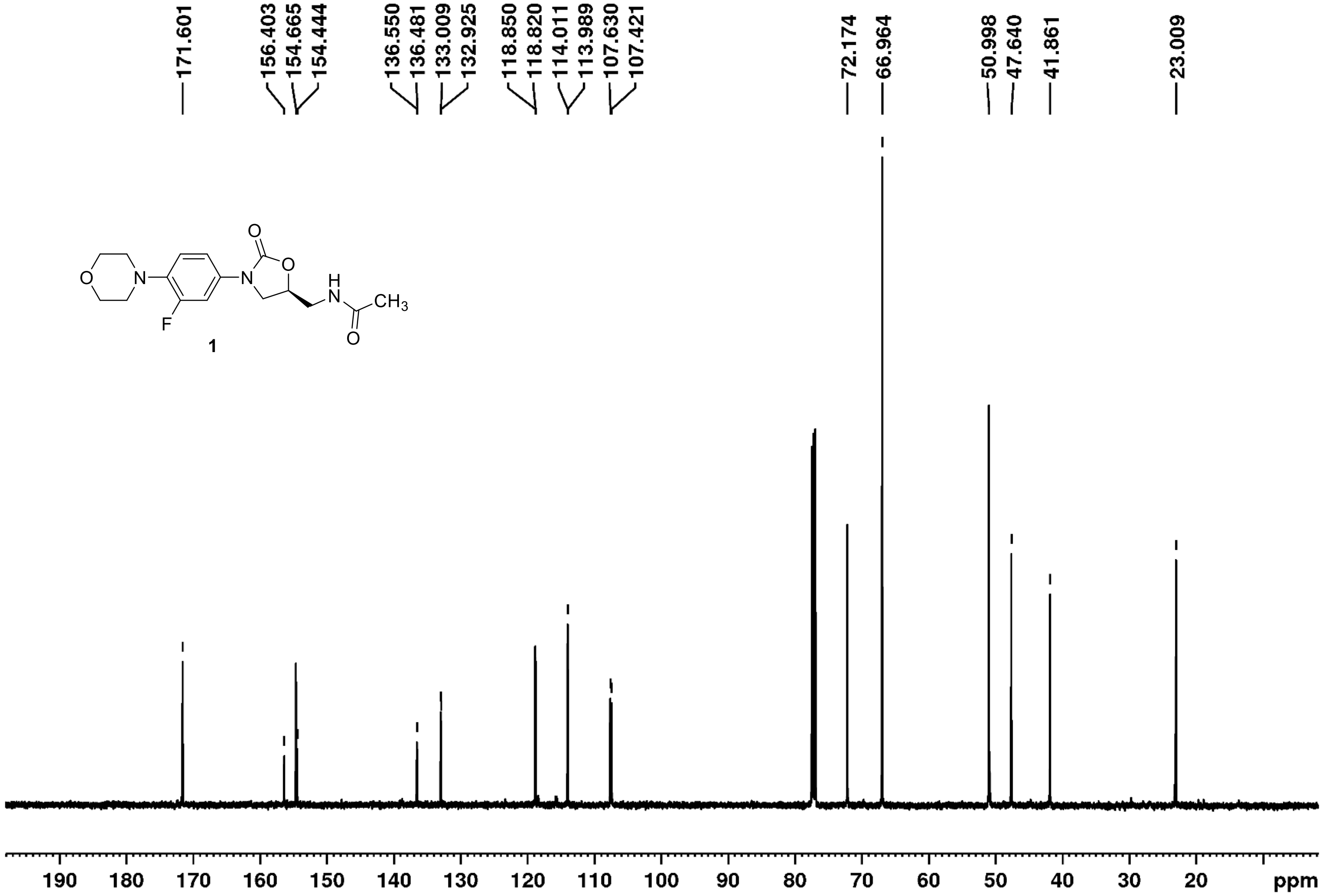
171.601
156.403
154.665
154.444
136.550
136.481
133.009
132.925
118.850
118.820
114.011
113.989
107.630
107.421
72.174
66.964
50.998
47.640
41.861
23.009
O
N
O
O
H
N
CH3
O
F
190 180 170 160 150 140 130 120 110 100 90 80 70 60 50 40 30 20 ppm

1

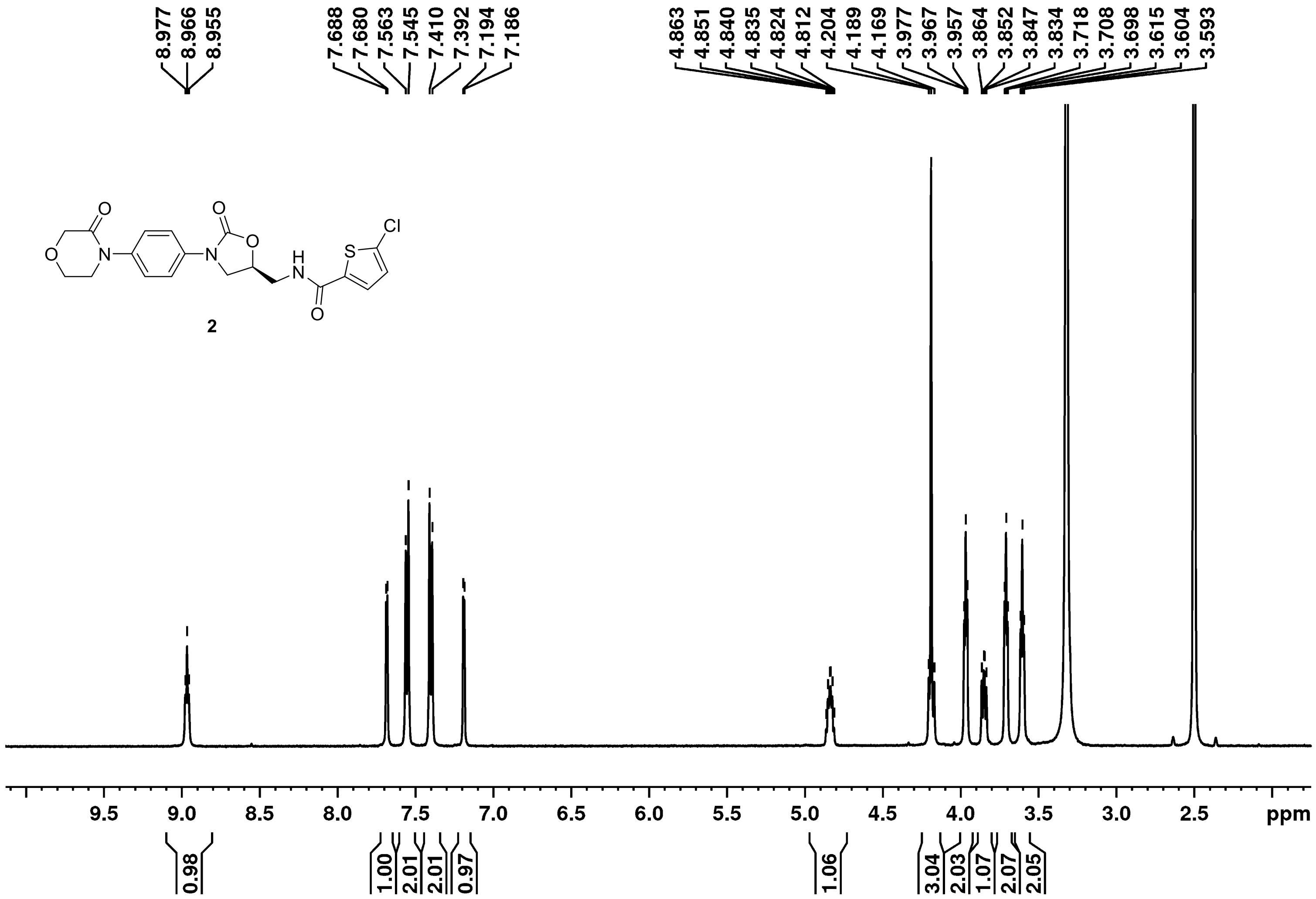
8.977
8.966
8.955
7.688
7.680
7.563
7.545
7.410
7.392
7.194
7.186
4.863
4.851
4.840
4.835
4.824
4.812
4.204
4.189
4.169
3.977
3.967
3.957
3.864
3.852
3.847
3.834
3.718
3.708
3.698
3.615
3.604
3.593
O
O
O
N
N
O
H
N
S
Cl
O
2
9.5
9.0
8.5
8.0
7.5
7.0
6.5
6.0
5.5
5.0
4.5
4.0
3.5
3.0
2.5
ppm
0.98
1.00
2.01
2.01
0.97
1.06
3.04
2.03
1.07
2.07
2.05

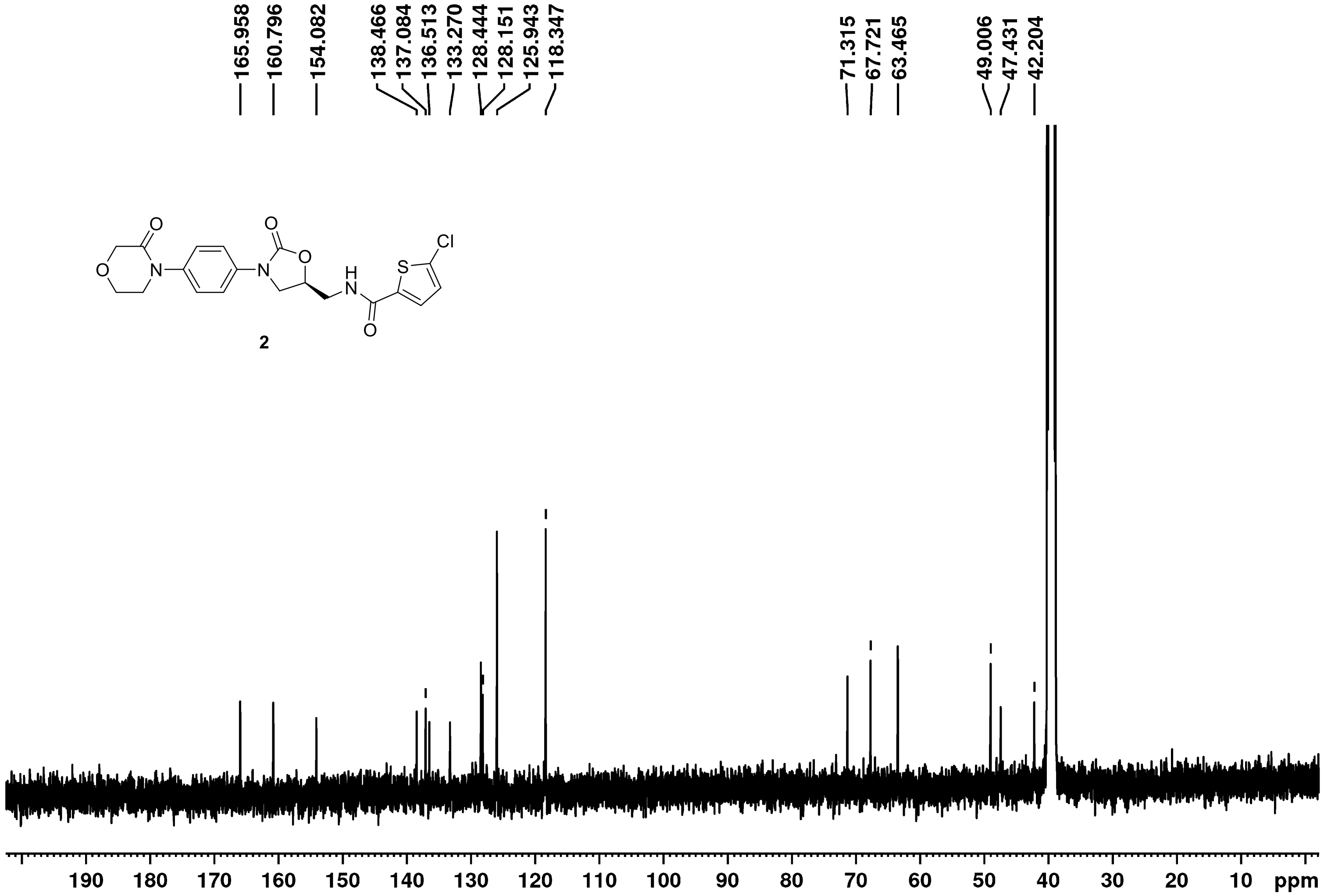
165.958
160.796
154.082
138.466
137.084
136.513
133.270
128.444
128.151
125.943
118.347
71.315
67.721
63.465
49.006
47.431
42.204
O
O
N
N
O
O
H
N
S
Cl
O
2
190 180 170 160 150 140 130 120 110 100 90 80 70 60 50 40 30 20 10 ppm

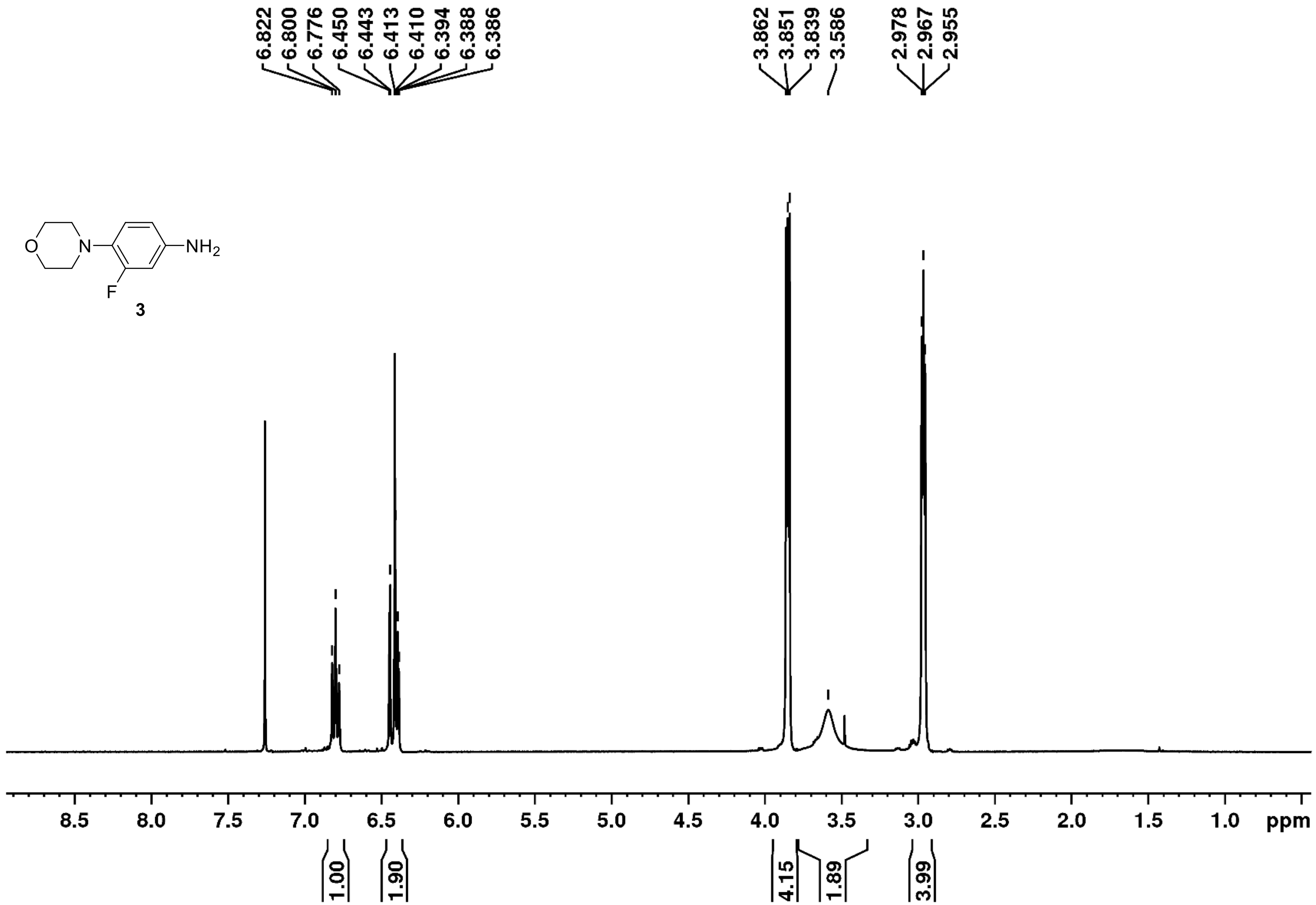
6.822
6.800
6.776
6.450
6.443
6.413
6.410
6.394
6.388
6.386
3.862
3.851
3.839
3.586
2.978
2.967
2.955
O
N
NH2
F
3
8.5 8.0 7.5 7.0 6.5 6.0 5.5 5.0 4.5 4.0 3.5 3.0 2.5 2.0 1.5 1.0 ppm
1.00
1.90
4.15
1.89
3.99

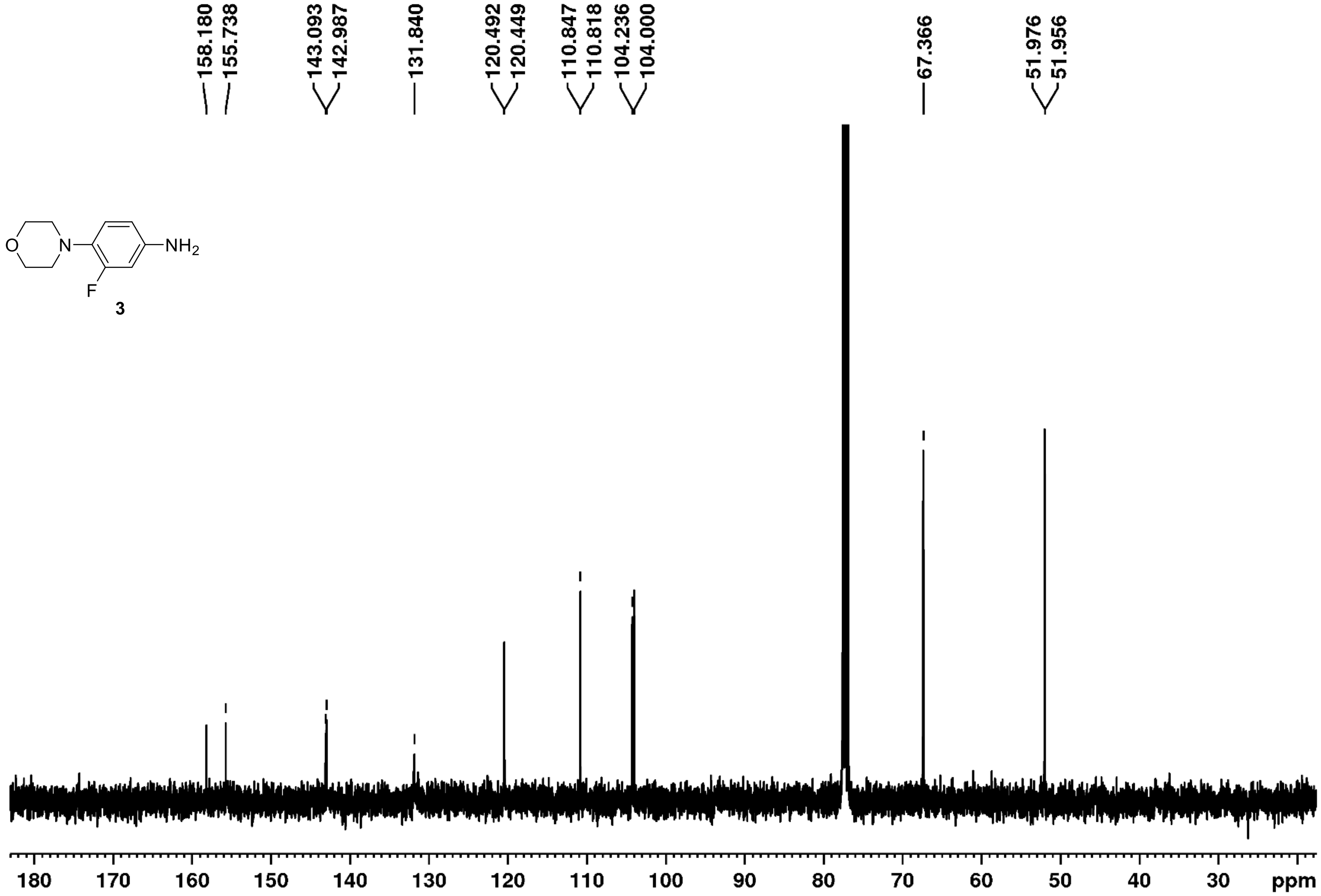
158.180
155.738
143.093
142.987
131.840
120.492
120.449
110.847
110.818
104.236
104.000
67.366
51.976
51.956
O
N
NH2
F
3
180
170
160
150
140
130
120
110
100
90
80
70
60
50
40
30
ppm

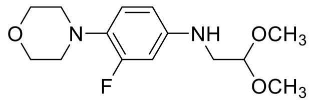

5

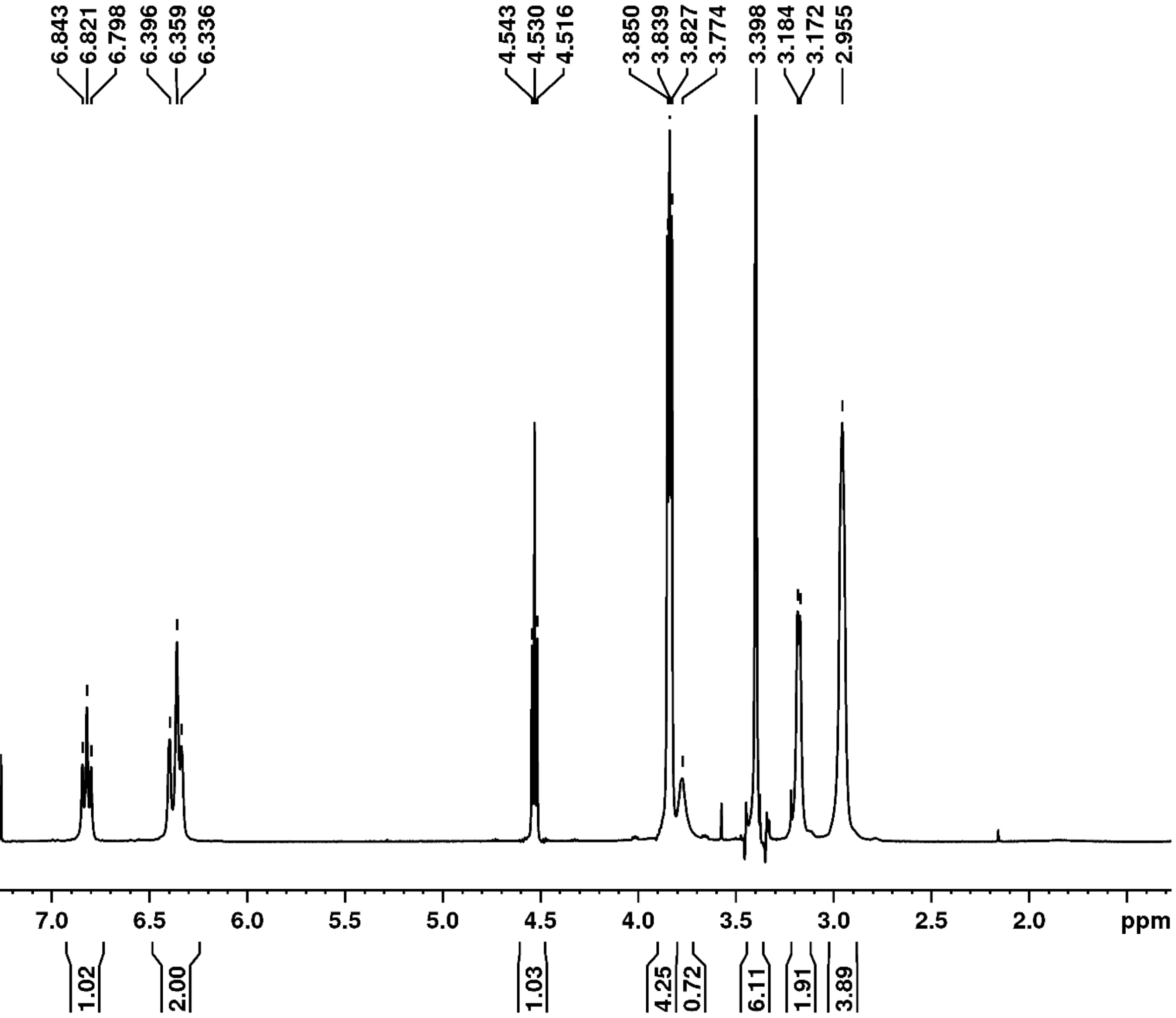
6.843
6.821
6.798
6.396
6.359
6.336
4.543
4.530
4.516
3.850
3.839
3.827
3.774
3.398
3.184
3.172
2.955
8.5
8.0
7.5
7.0
6.5
6.0
5.5
5.0
4.5
4.0
3.5
3.0
2.5
2.0
ppm
1.02
2.00
1.03
4.25
0.72
6.11
1.91
3.89

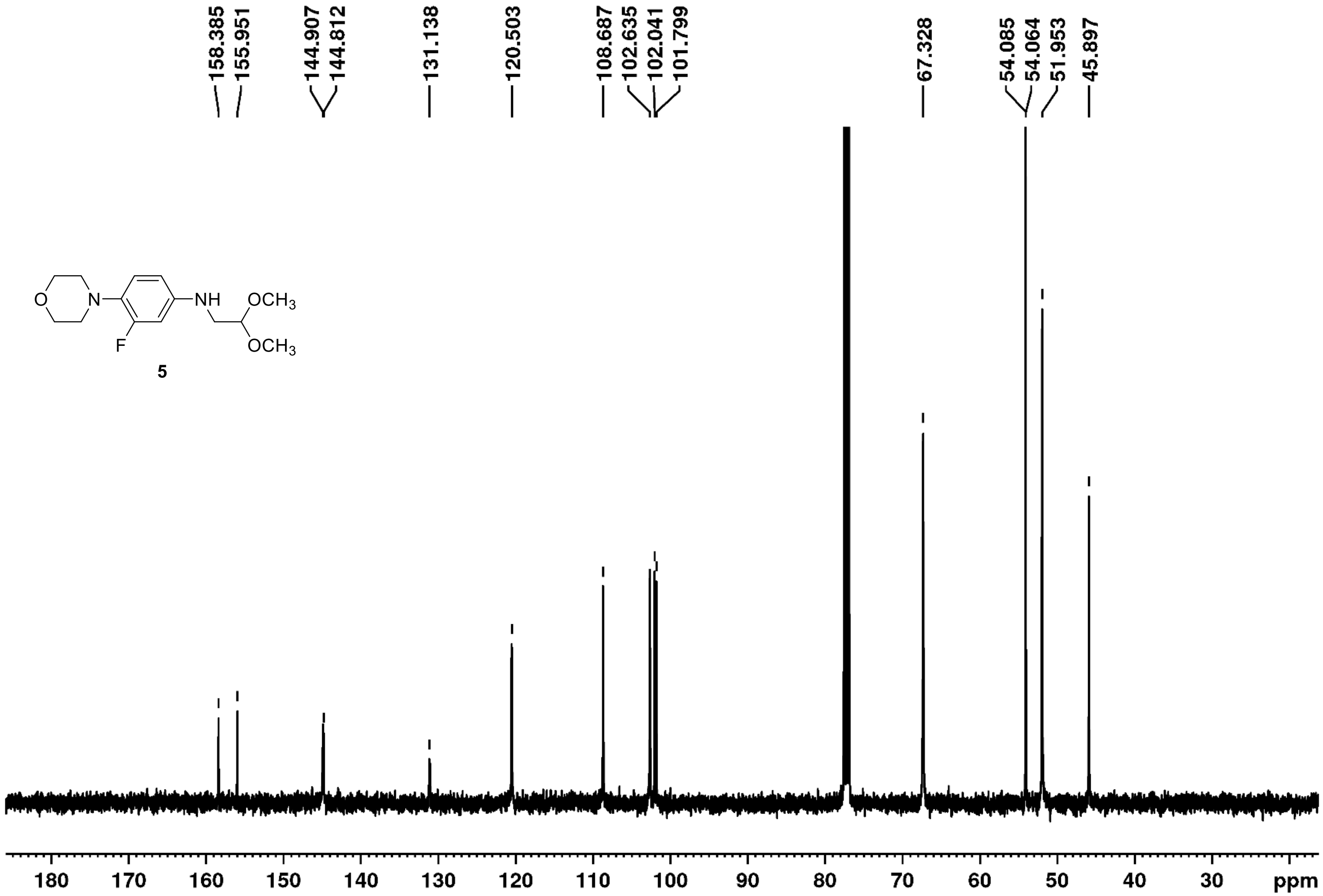
158.385
155.951
144.907
144.812
131.138
120.503
108.687
102.635
102.041
101.799
67.328
54.085
54.064
51.953
45.897
O
N
NH
OCH3
OCH3
F
5
180 170 160 150 140 130 120 110 100 90 80 70 60 50 40 30 ppm

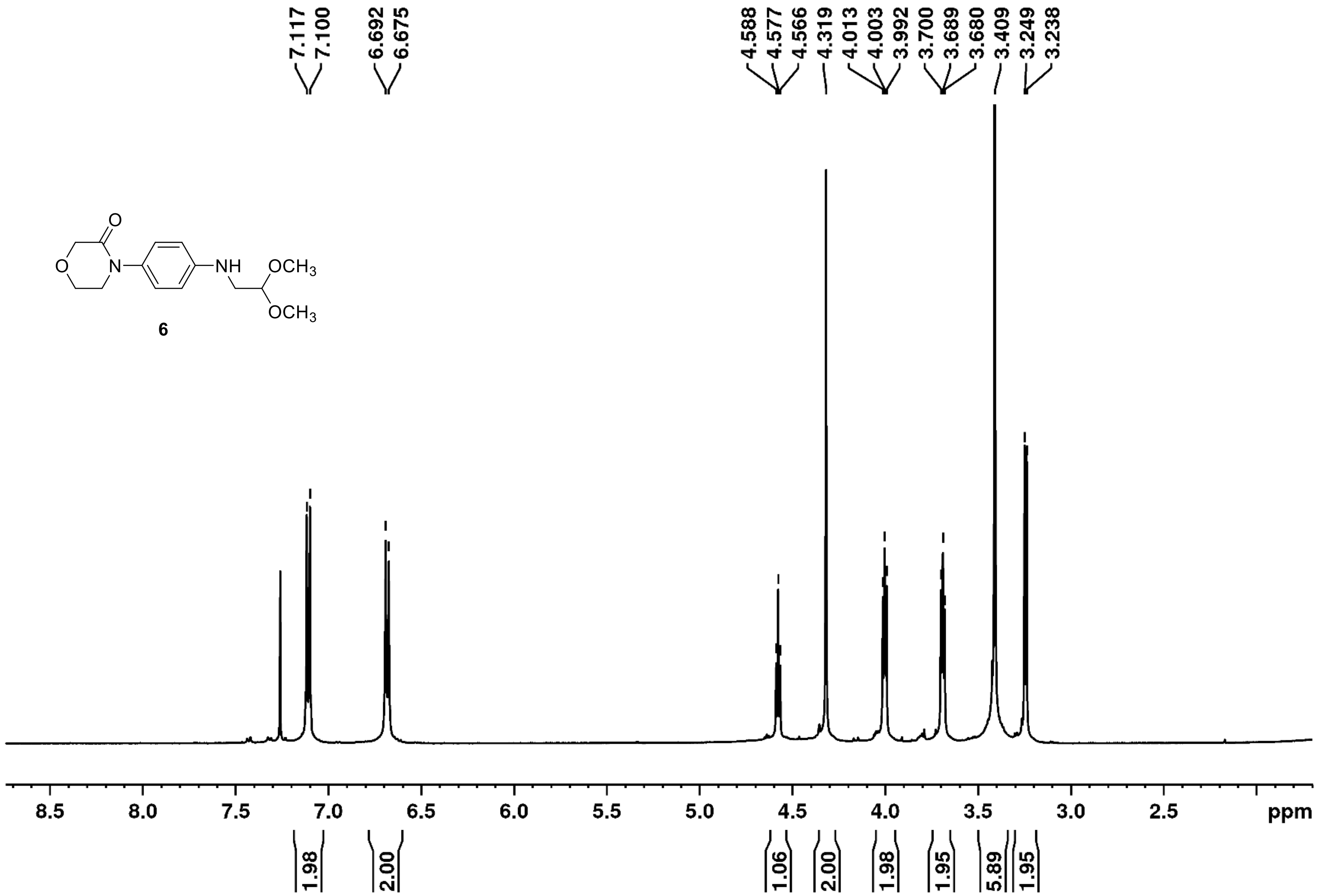
7.117
7.100
6.692
6.675
4.588
4.577
4.566
4.319
4.013
4.003
3.992
3.700
3.689
3.680
3.409
3.249
3.238
O
O
N
NH
OCH3
OCH3
6
8.5
8.0
7.5
7.0
6.5
6.0
5.5
5.0
4.5
4.0
3.5
3.0
2.5
ppm
1.98
2.00
1.06
2.00
1.98
1.95
5.89
1.95

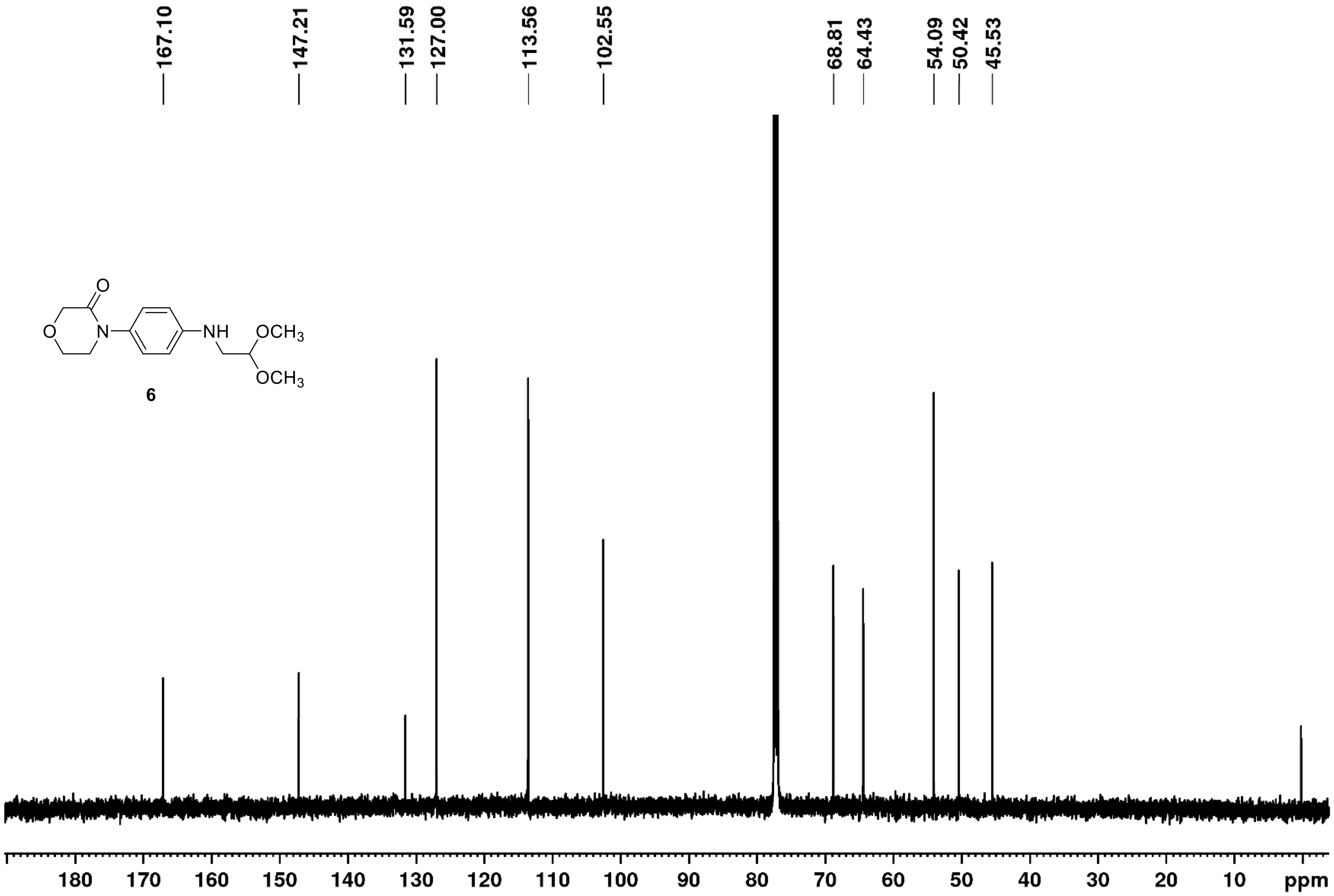
167.10
147.21
131.59
127.00
113.56
102.55
68.81
64.43
54.09
50.42
45.53
O
O
N
NH
OCH3
OCH3
6
180 170 160 150 140 130 120 110 100 90 80 70 60 50 40 30 20 10 ppm

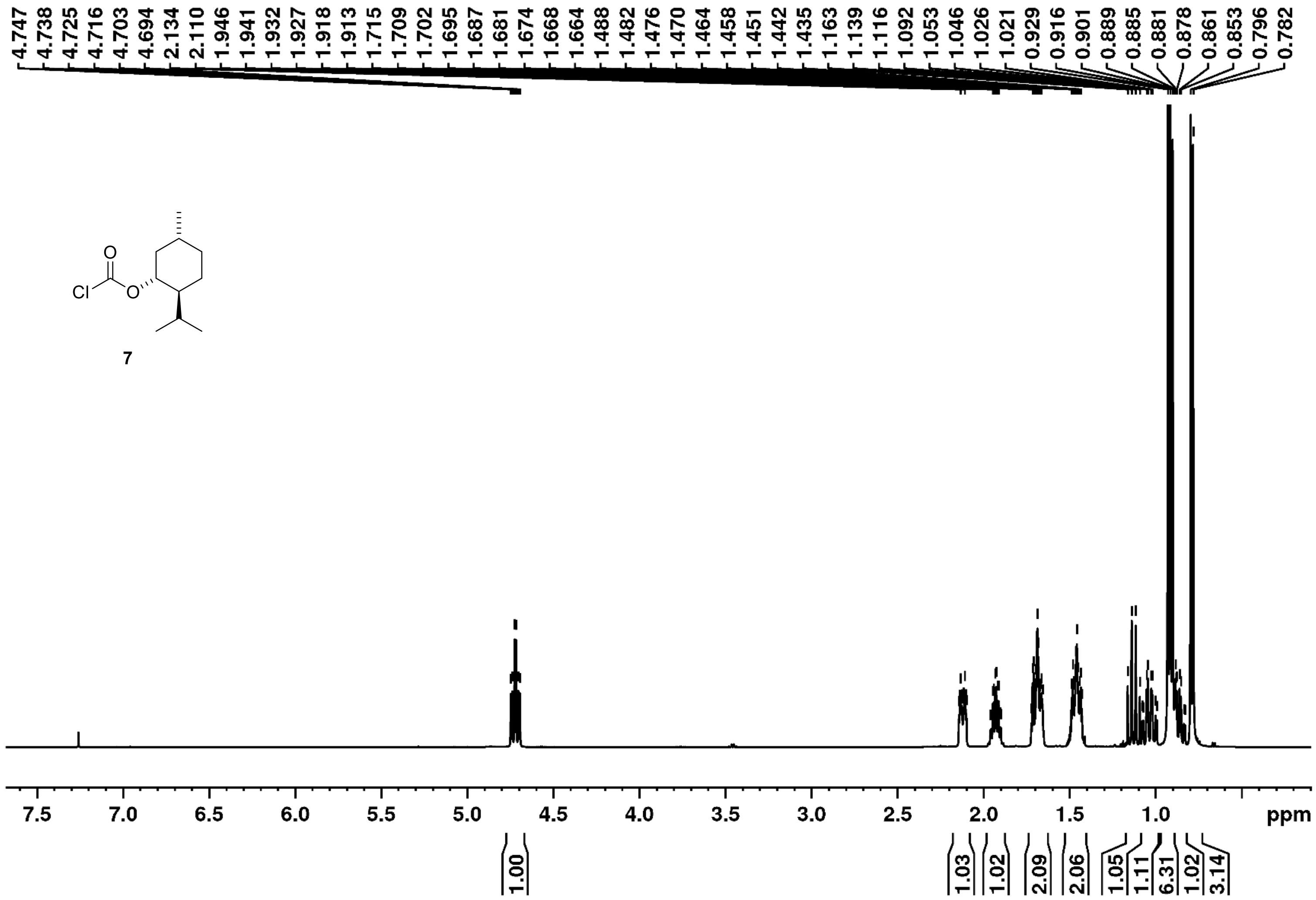
4.747
4.738
4.725
4.716
4.703
4.694
2.134
2.110
1.946
1.941
1.932
1.927
1.918
1.913
1.715
1.709
1.702
1.695
1.687
1.681
1.674
1.668
1.664
1.488
1.482
1.476
1.470
1.464
1.458
1.451
1.442
1.435
1.163
1.139
1.116
1.092
1.053
1.046
1.026
1.021
0.929
0.916
0.901
0.889
0.885
0.881
0.878
0.861
0.853
0.796
0.782
O
Cl
O
7
7.5
7.0
6.5
6.0
5.5
5.0
4.5
4.0
3.5
3.0
2.5
2.0
1.5
1.0
ppm
1.00
1.03
1.02
2.09
2.06
1.05
1.11
6.31
1.02
3.14

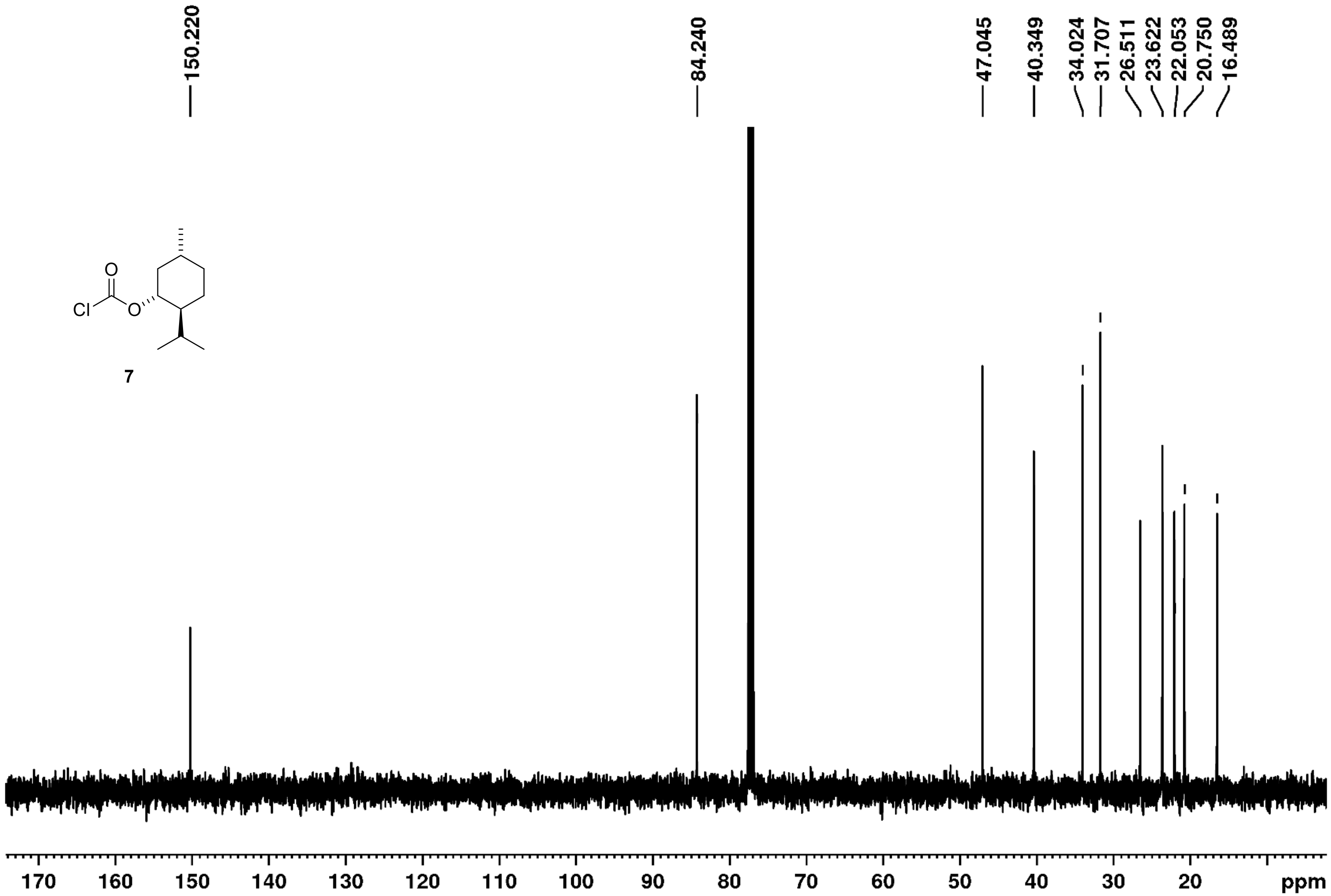
150.220
84.240
47.045
40.349
34.024
31.707
26.511
23.622
22.053
20.750
16.489
O
Cl
O
7
170 160 150 140 130 120 110 100 90 80 70 60 50 40 30 20 ppm

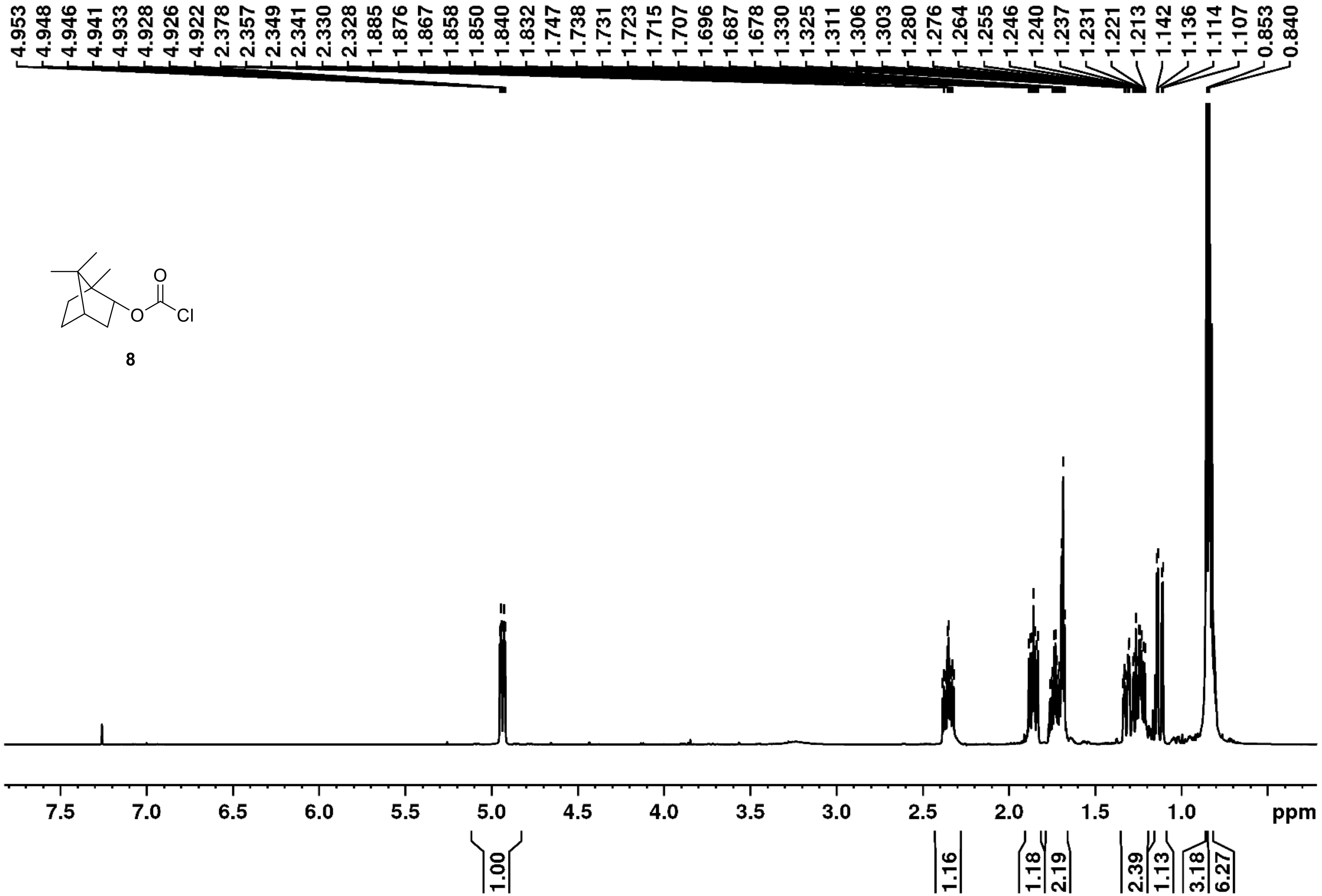
4.953 4.948 4.946 4.941 4.933 4.928 4.926 4.922 2.378 2.357 2.349 2.341 2.330 2.328 1.885 1.876 1.867 1.858 1.850 1.840 1.832 1.747 1.738 1.731 1.723 1.715 1.707 1.696 1.687 1.678 1.330 1.325 1.311 1.306 1.303 1.280 1.276 1.264 1.255 1.246 1.240 1.237 1.231 1.221 1.213 1.142 1.136 1.114 1.107 0.853 0.840
O
O
Cl
8
7.5 7.0 6.5 6.0 5.5 5.0 4.5 4.0 3.5 3.0 2.5 2.0 1.5 1.0 ppm
1.00
1.16
1.18
2.19
2.39
1.13
3.18
6.27

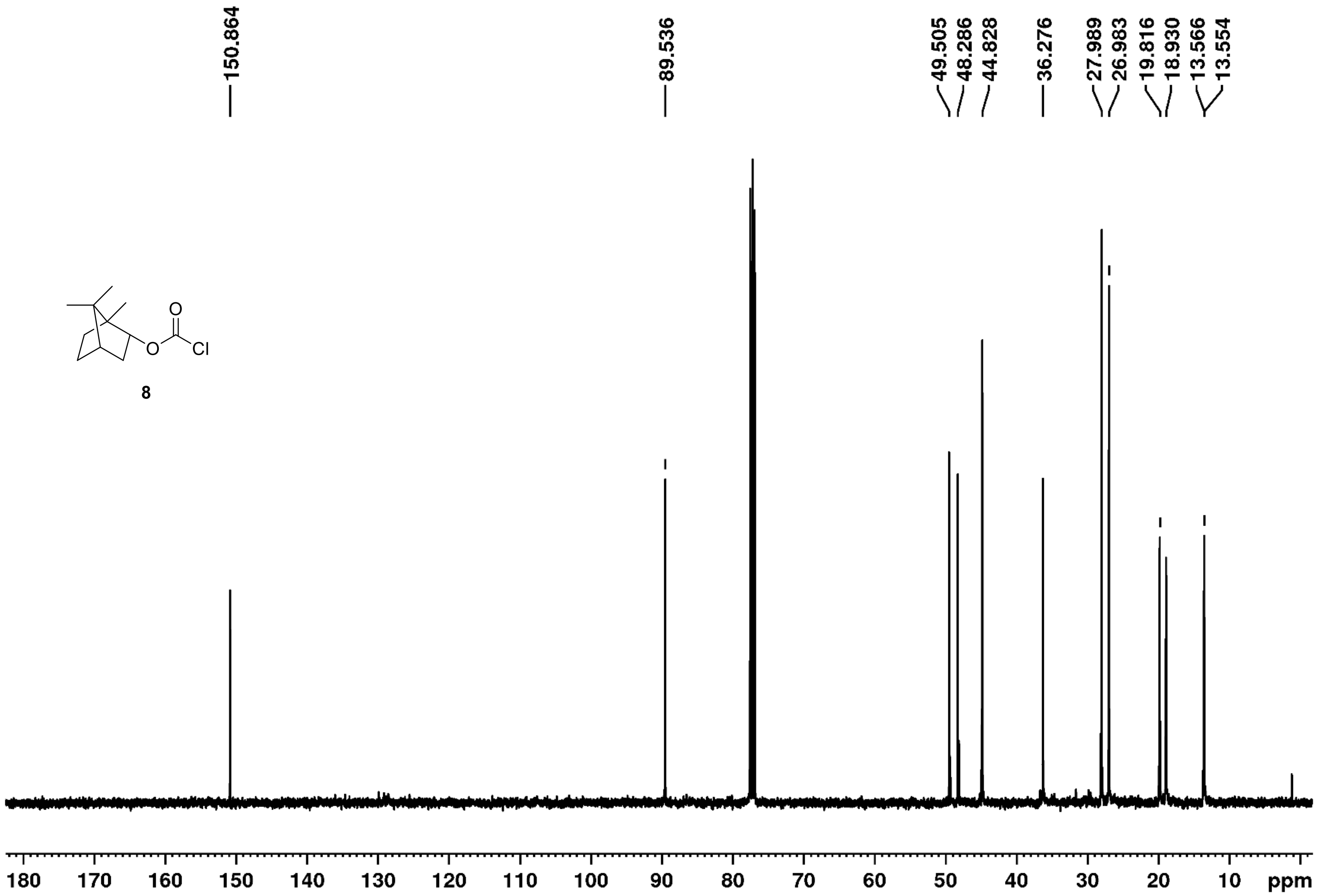
150.864
89.536
49.505
48.286
44.828
36.276
27.989
26.983
19.816
18.930
13.566
13.554
O
O
Cl
8
180
170
160
150
140
130
120
110
100
90
80
70
60
50
40
30
20
10
ppm

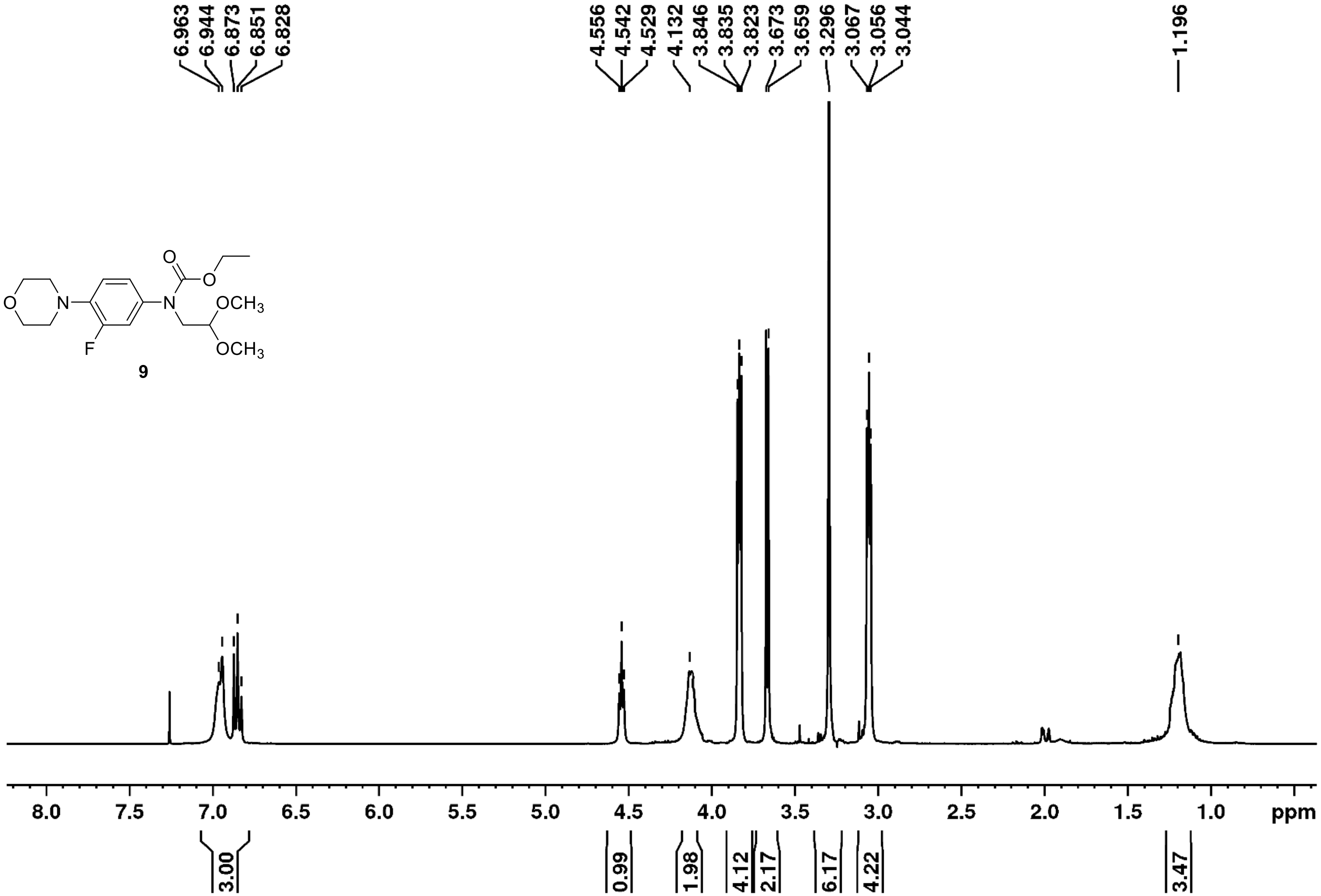
6.963
6.944
6.873
6.851
6.828
4.556
4.542
4.529
4.132
3.846
3.835
3.823
3.673
3.659
3.296
3.067
3.056
3.044
1.196
O
OCH$_3$
OCH$_3$
F
9
8.0 7.5 7.0 6.5 6.0 5.5 5.0 4.5 4.0 3.5 3.0 2.5 2.0 1.5 1.0 ppm
3.00
0.99
1.98
4.12
2.17
6.17
4.22
3.47

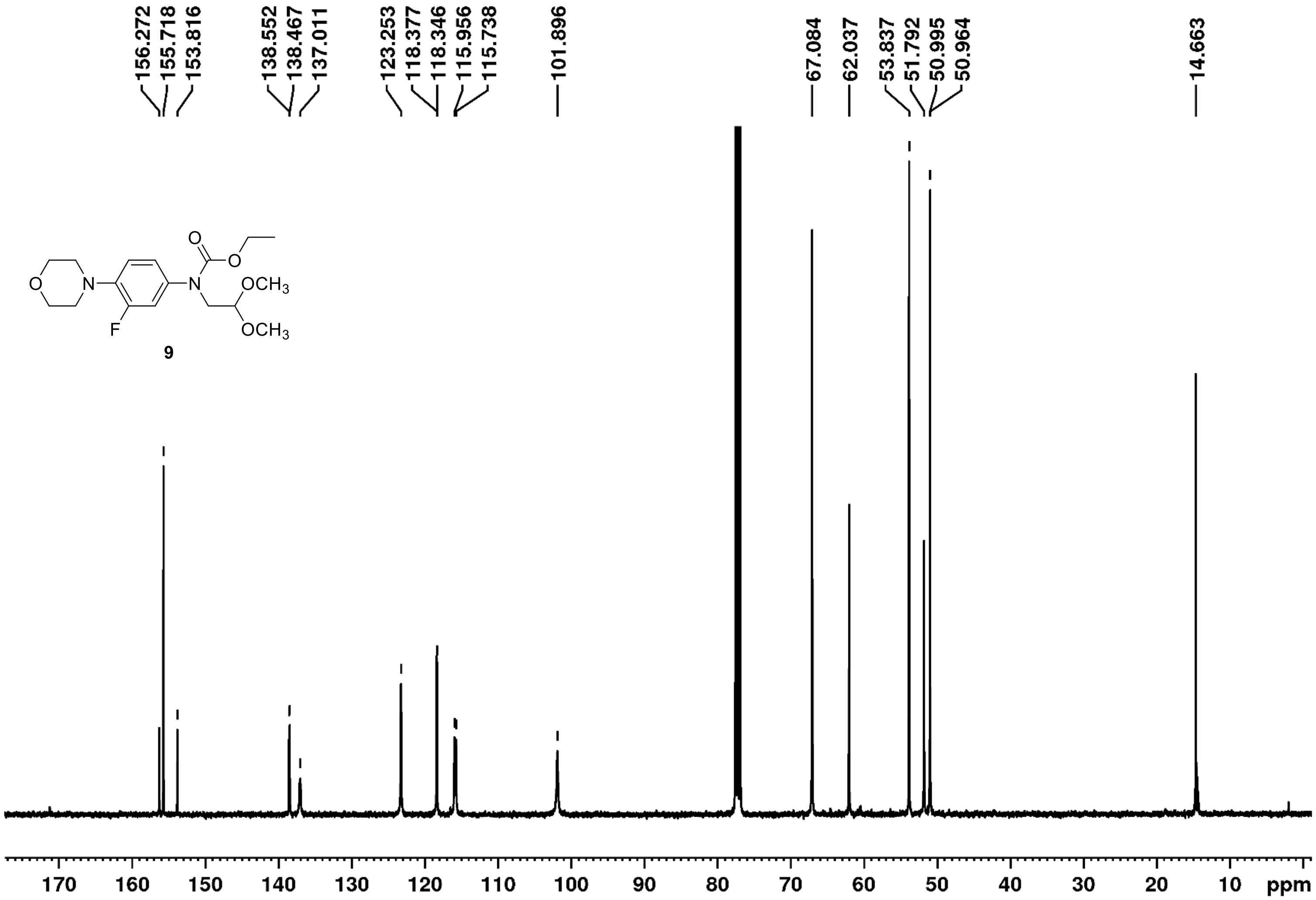
156.272
155.718
153.816
138.552
138.467
137.011
123.253
118.377
118.346
115.956
115.738
101.896
67.084
62.037
53.837
51.792
50.995
50.964
14.663
O
O
O
N
N
OCH3
OCH3
F
9
170 160 150 140 130 120 110 100 90 80 70 60 50 40 30 20 10 ppm

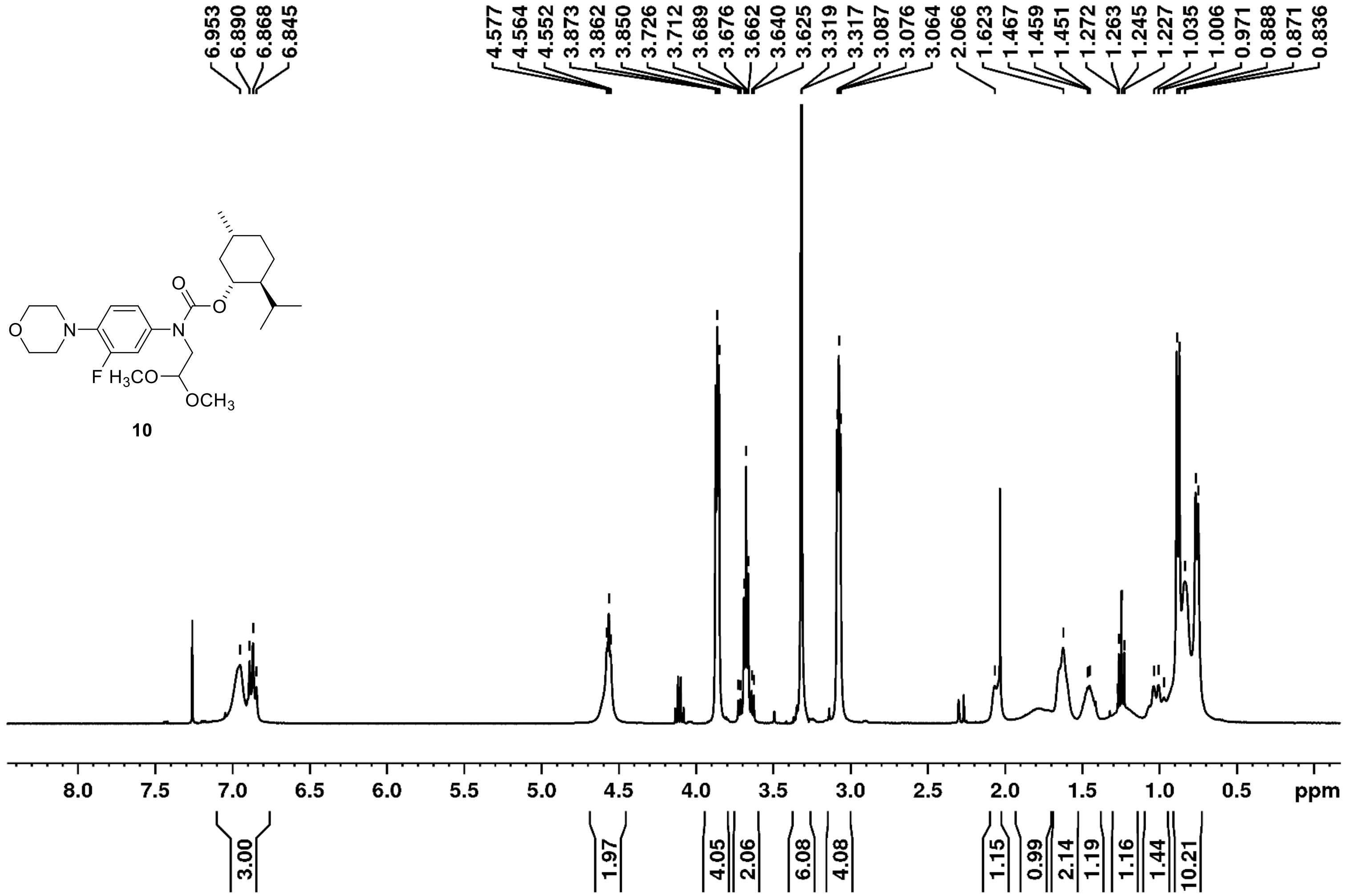
6.953
6.890
6.868
6.845
4.577
4.564
4.552
3.873
3.862
3.850
3.726
3.712
3.689
3.676
3.662
3.640
3.625
3.319
3.317
3.087
3.076
3.064
2.066
1.623
1.467
1.459
1.451
1.272
1.263
1.245
1.227
1.035
1.006
0.971
0.888
0.871
0.836
10
8.0
7.5
7.0
6.5
6.0
5.5
5.0
4.5
4.0
3.5
3.0
2.5
2.0
1.5
1.0
0.5
ppm
3.00
1.97
4.05
2.06
6.08
4.08
1.15
0.99
2.14
1.19
1.16
1.44
10.21

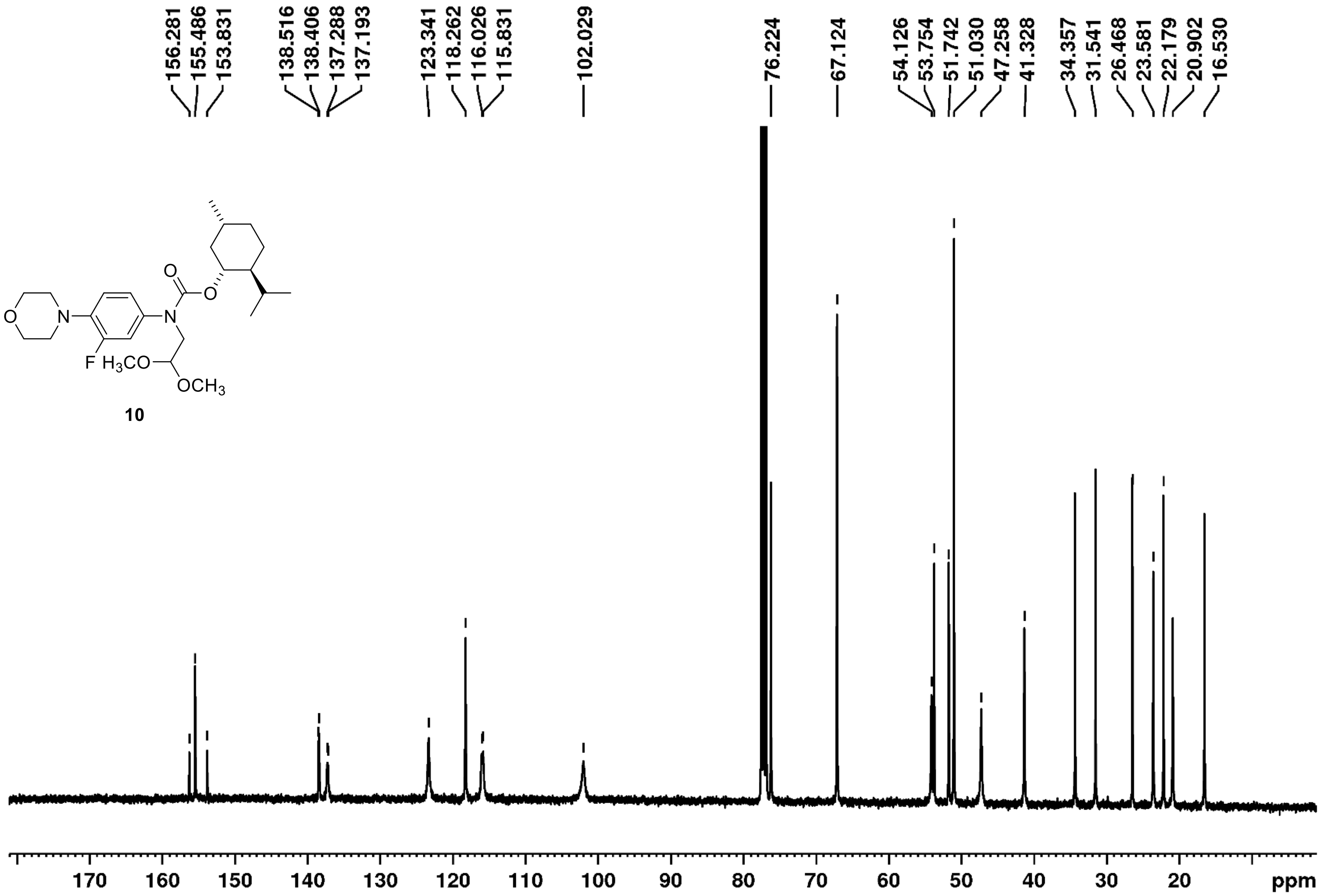
156.281
155.486
153.831
138.516
138.406
137.288
137.193
123.341
118.262
116.026
115.831
102.029
76.224
67.124
54.126
53.754
51.742
51.030
47.258
41.328
34.357
31.541
26.468
23.581
22.179
20.902
16.530
O
N
O
O
N
F
H_3CO
OCH_3
10
170
160
150
140
130
120
110
100
90
80
70
60
50
40
30
20
ppm

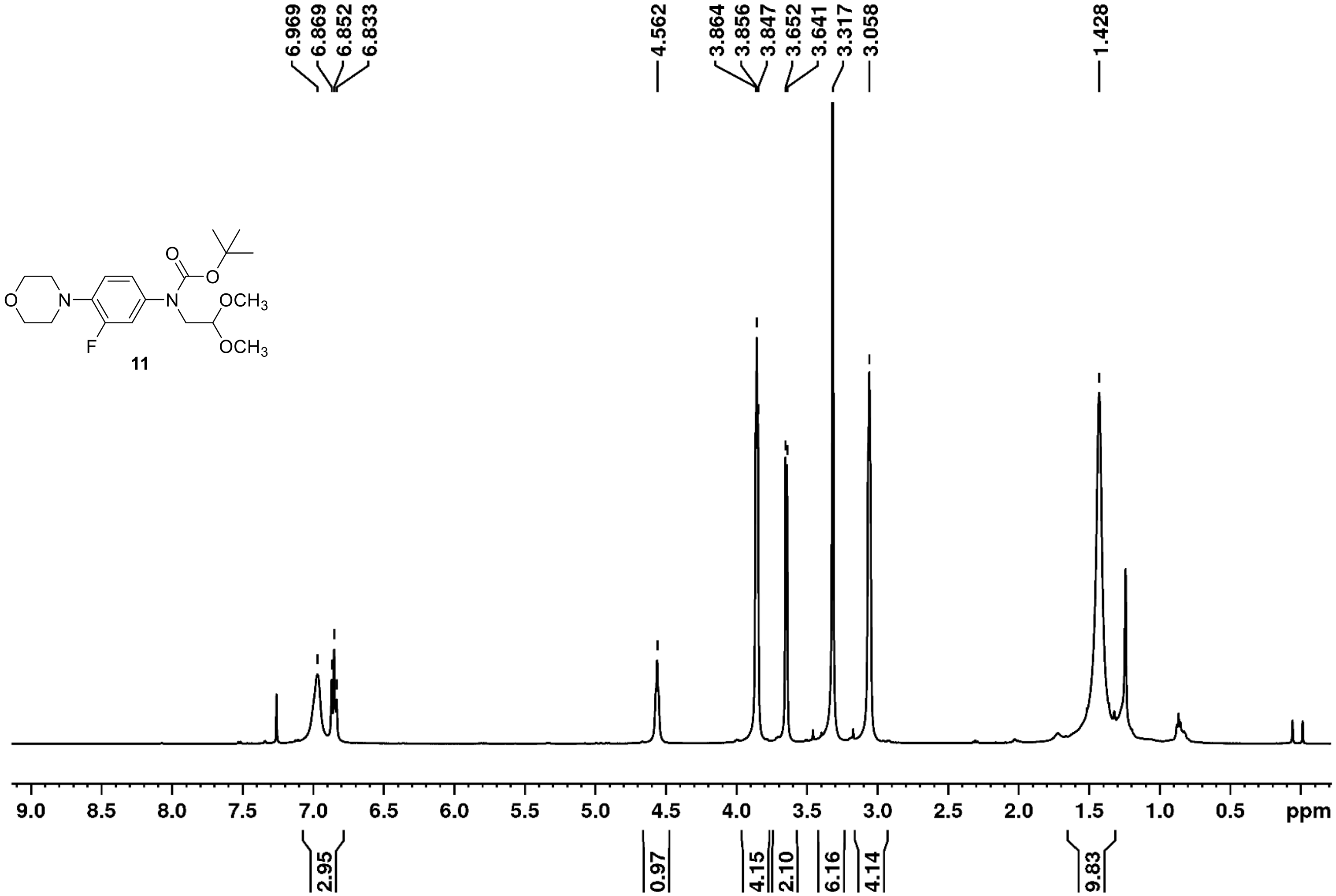
6.969
6.869
6.852
6.833
4.562
3.864
3.856
3.847
3.652
3.641
3.317
3.058
1.428
O
N
F
N
O
O
OCH3
OCH3
11
9.0 8.5 8.0 7.5 7.0 6.5 6.0 5.5 5.0 4.5 4.0 3.5 3.0 2.5 2.0 1.5 1.0 0.5 ppm
2.95
0.97
4.15
2.10
6.16
4.14
9.83

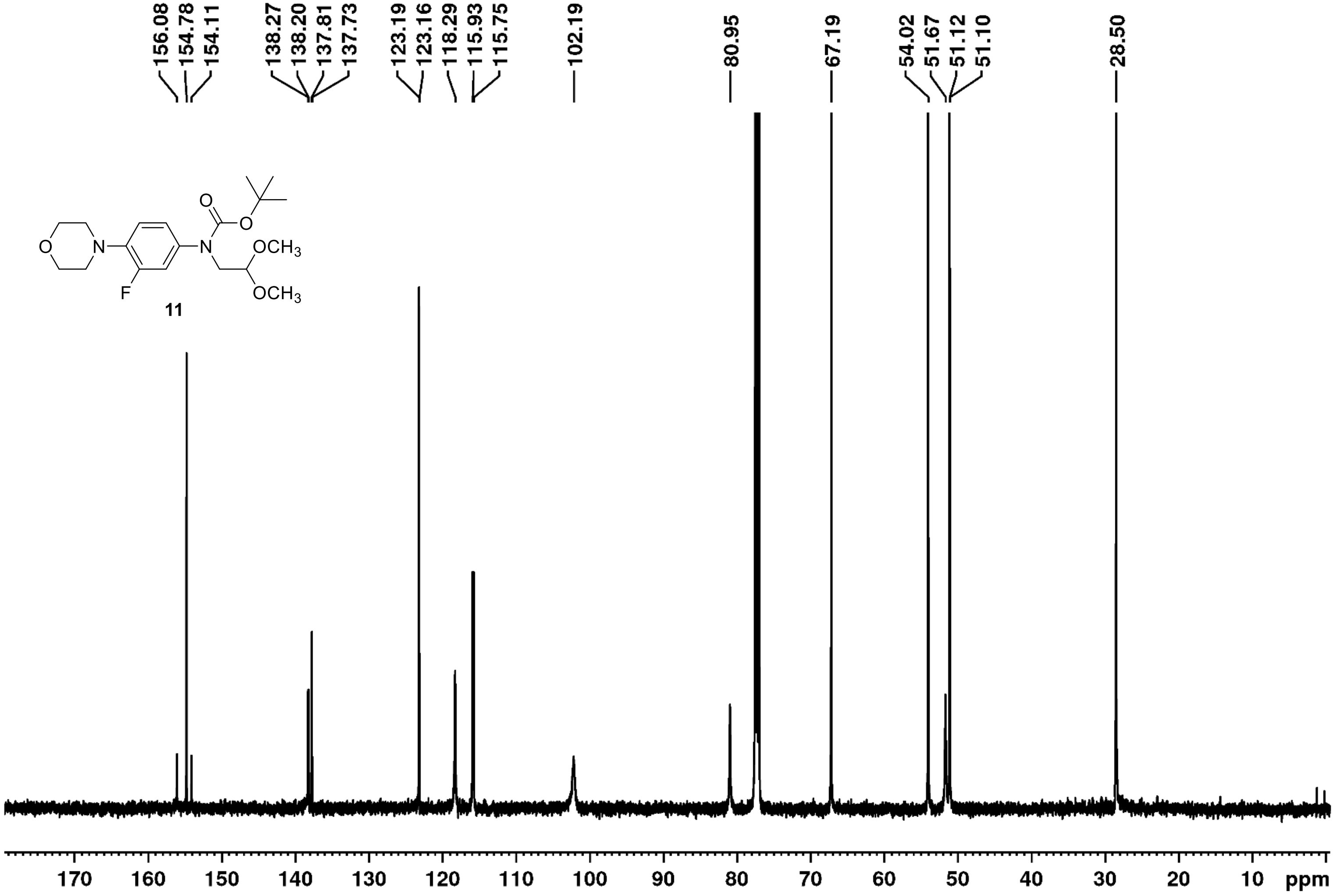

156.08
154.78
154.11
138.27
138.20
137.81
137.73
123.19
123.16
118.29
115.93
115.75
102.19
80.95
67.19
54.02
51.67
51.12
51.10
28.50
O
N
F
N
O
O
OCH3
OCH3
11
170
160
150
140
130
120
110
100
90
80
70
60
50
40
30
20
10
ppm

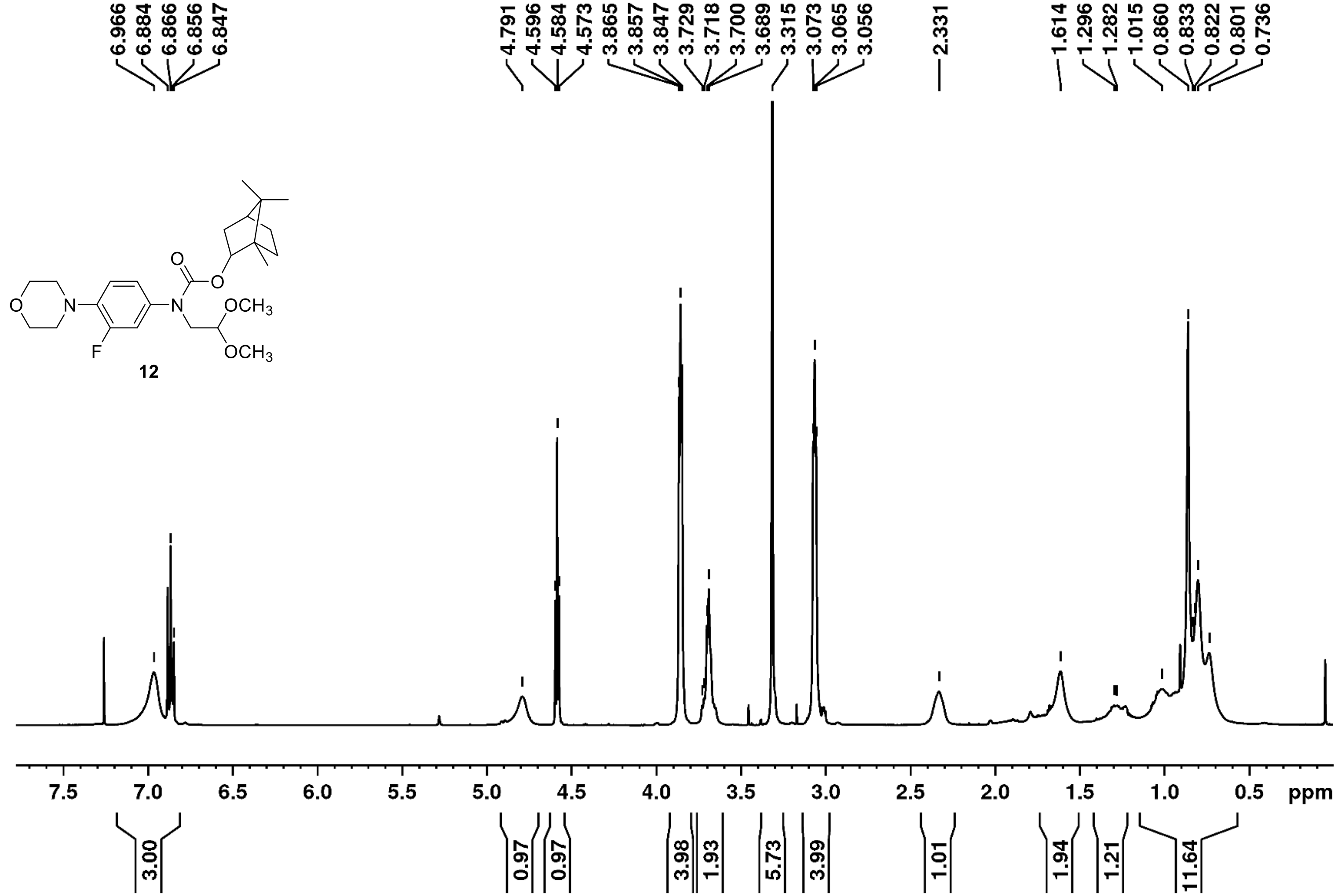
6.966
6.884
6.866
6.856
6.847
4.791
4.596
4.584
4.573
3.865
3.857
3.847
3.729
3.718
3.700
3.689
3.315
3.073
3.065
3.056
2.331
1.614
1.296
1.282
1.015
0.860
0.833
0.822
0.801
0.736
O
N
F
N
O
O
OCH3
OCH3
12
7.5 7.0 6.5 6.0 5.5 5.0 4.5 4.0 3.5 3.0 2.5 2.0 1.5 1.0 0.5 ppm
3.00
0.97
0.97
3.98
1.93
5.73
3.99
1.01
1.94
1.21
11.64

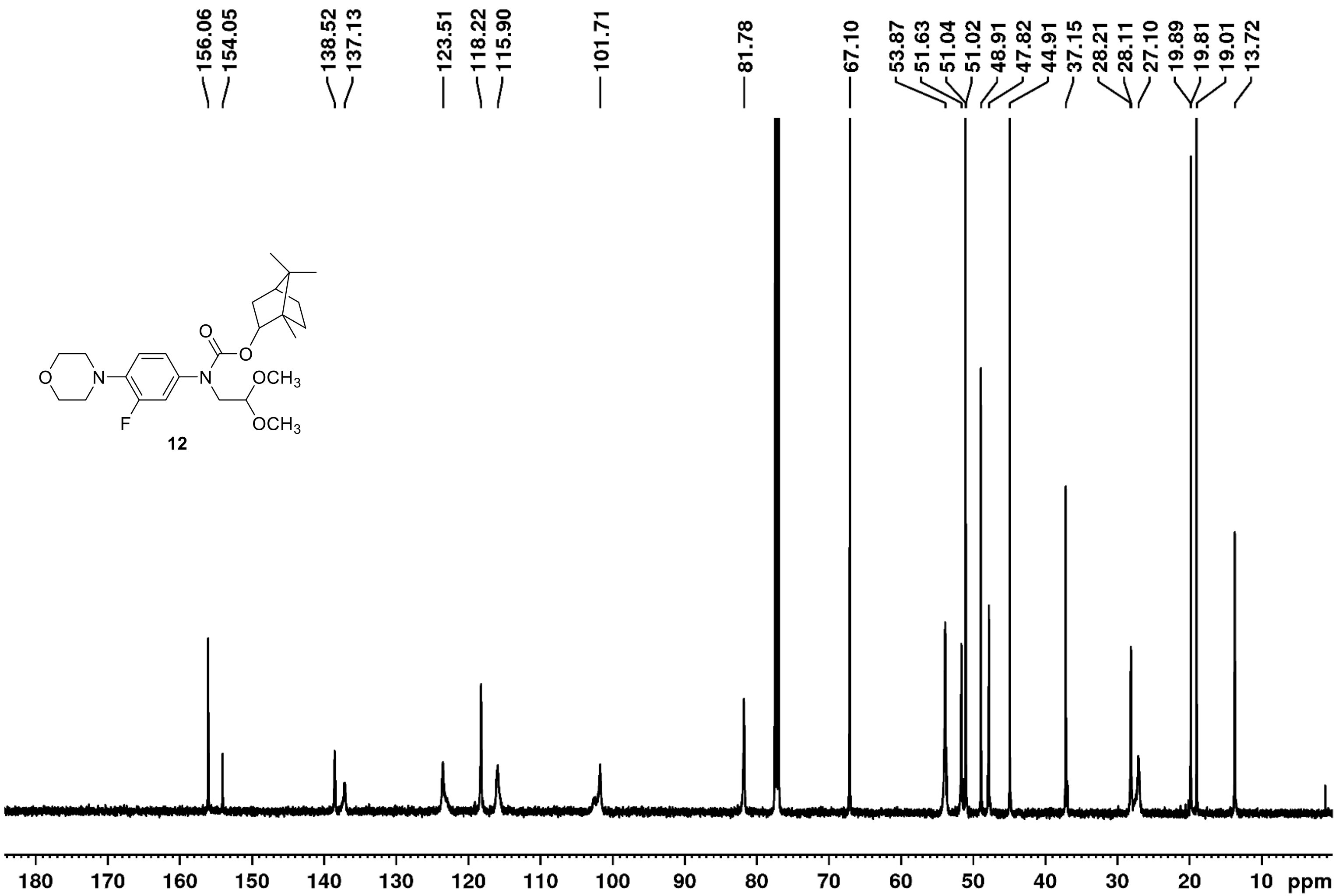
156.06
154.05
138.52
137.13
123.51
118.22
115.90
101.71
81.78
67.10
53.87
51.63
51.04
51.02
48.91
47.82
44.91
37.15
28.21
28.11
27.10
19.89
19.81
19.01
13.72
O
N
F
O
O
N
OCH3
OCH3
12
180
170
160
150
140
130
120
110
100
90
80
70
60
50
40
30
20
10
ppm

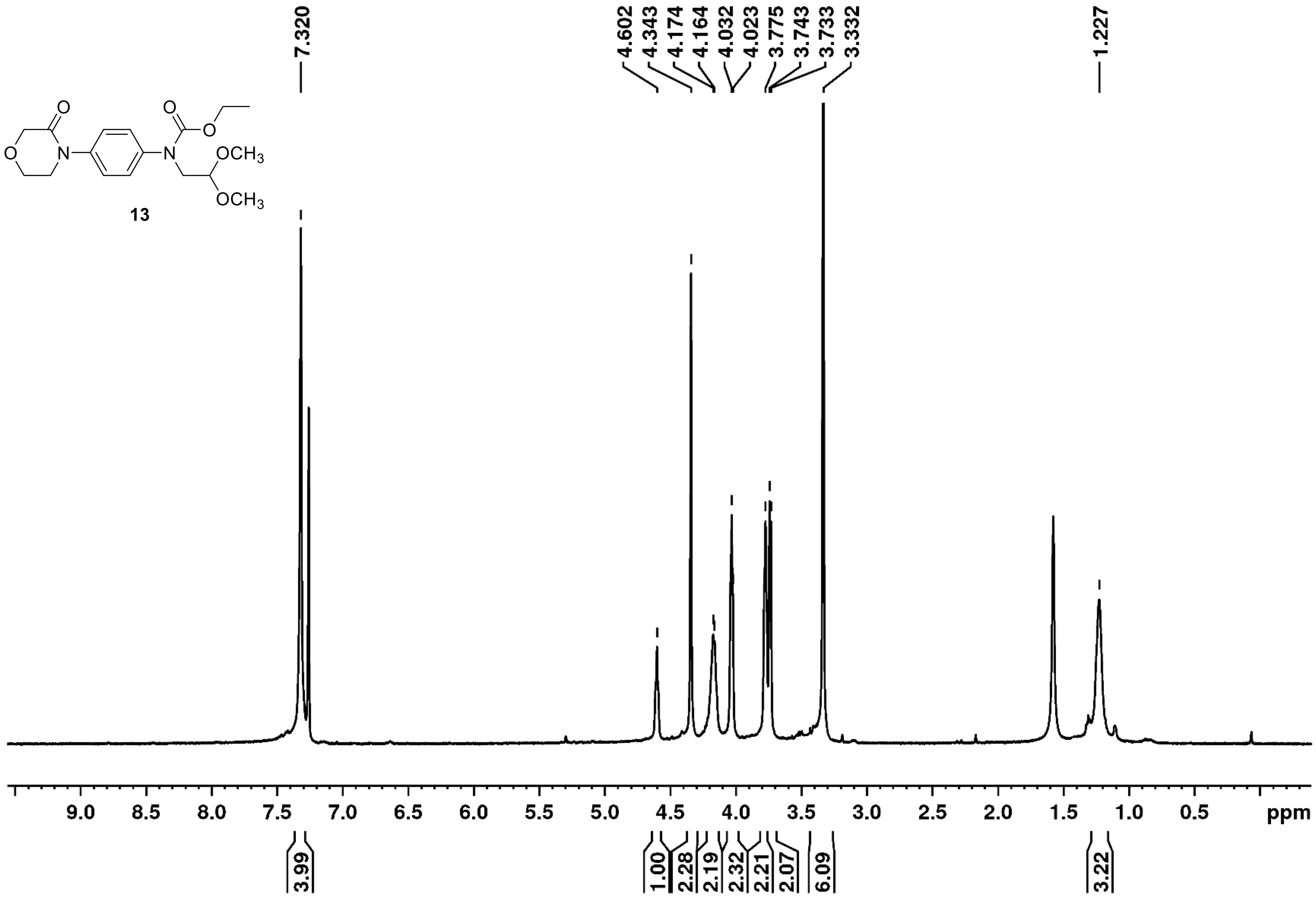

O
O
N
O
O
N
OCH3
OCH3
13
7.320
4.602
4.343
4.174
4.164
4.032
4.023
3.775
3.743
3.733
3.332
1.227
9.0 8.5 8.0 7.5 7.0 6.5 6.0 5.5 5.0 4.5 4.0 3.5 3.0 2.5 2.0 1.5 1.0 0.5 ppm
3.99
1.00
2.28
2.19
2.32
2.21
2.07
6.09
3.22

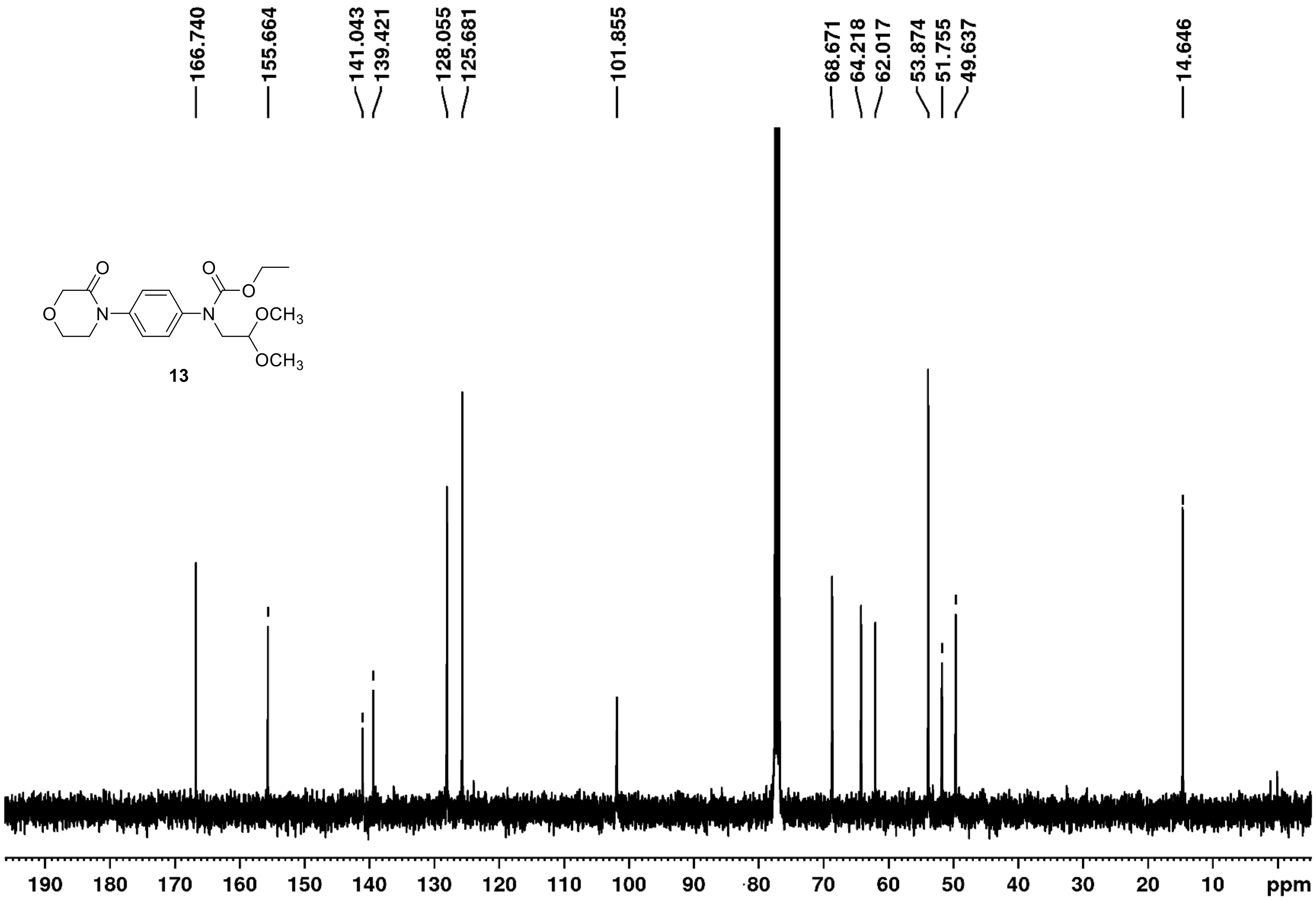
166.740
155.664
141.043
139.421
128.055
125.681
101.855
68.671
64.218
62.017
53.874
51.755
49.637
14.646
O
O
N
N
O
O
OCH3
OCH3
13
190 180 170 160 150 140 130 120 110 100 90 80 70 60 50 40 30 20 10 ppm

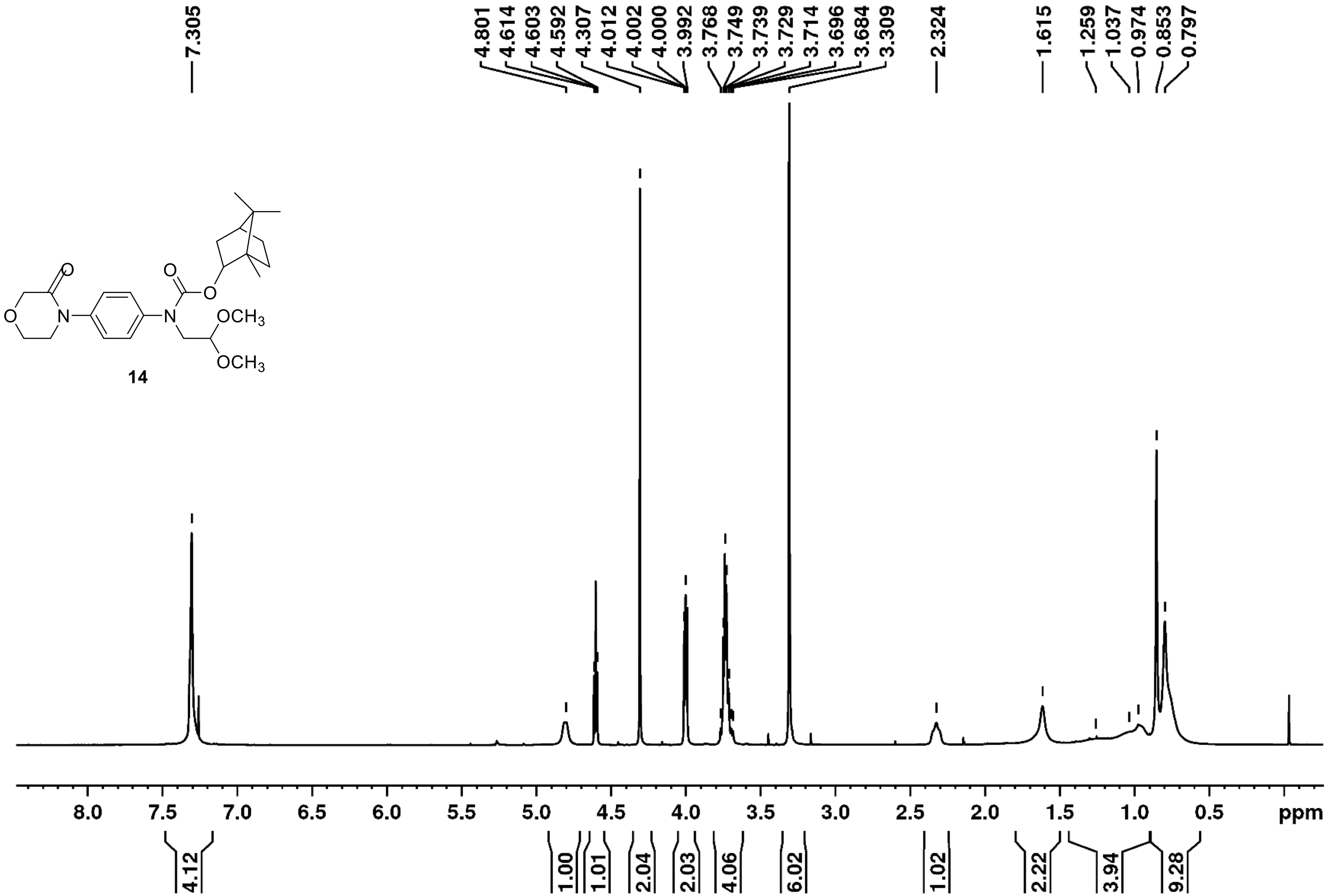

7.305
4.801
4.614
4.603
4.592
4.307
4.012
4.002
4.000
3.992
3.768
3.749
3.739
3.729
3.714
3.696
3.684
3.309
2.324
1.615
1.259
1.037
0.974
0.853
0.797
O
O
O
N
N
O
OCH3
OCH3
14
8.0
7.5
7.0
6.5
6.0
5.5
5.0
4.5
4.0
3.5
3.0
2.5
2.0
1.5
1.0
0.5
ppm
4.12
1.00
1.01
2.04
2.03
4.06
6.02
1.02
2.22
3.94
9.28

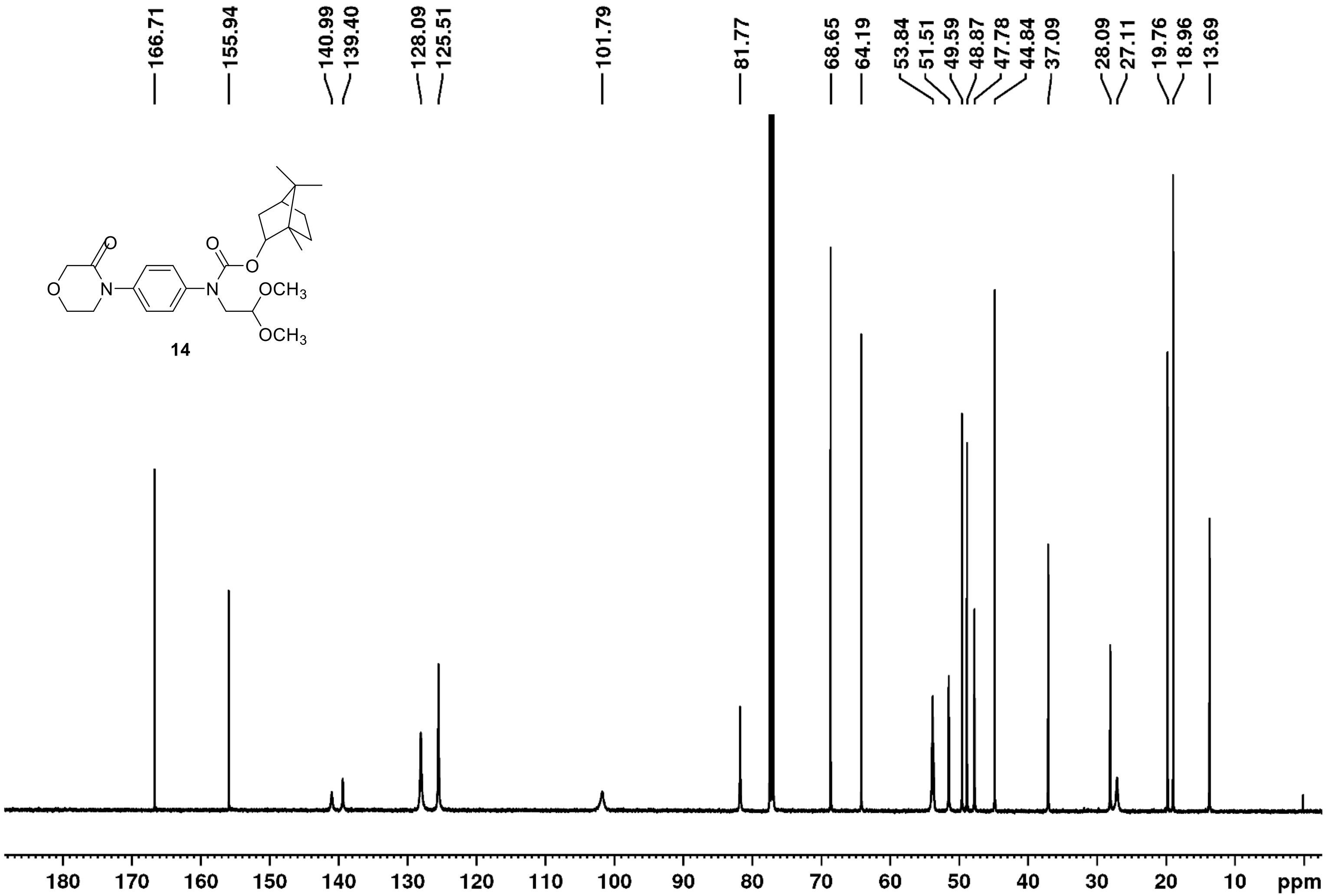
166.71
155.94
140.99
139.40
128.09
125.51
101.79
81.77
68.65
64.19
53.84
51.51
49.59
48.87
47.78
44.84
37.09
28.09
27.11
19.76
18.96
13.69
OCH3
OCH3
14
180
170
160
150
140
130
120
110
100
90
80
70
60
50
40
30
20
10
ppm

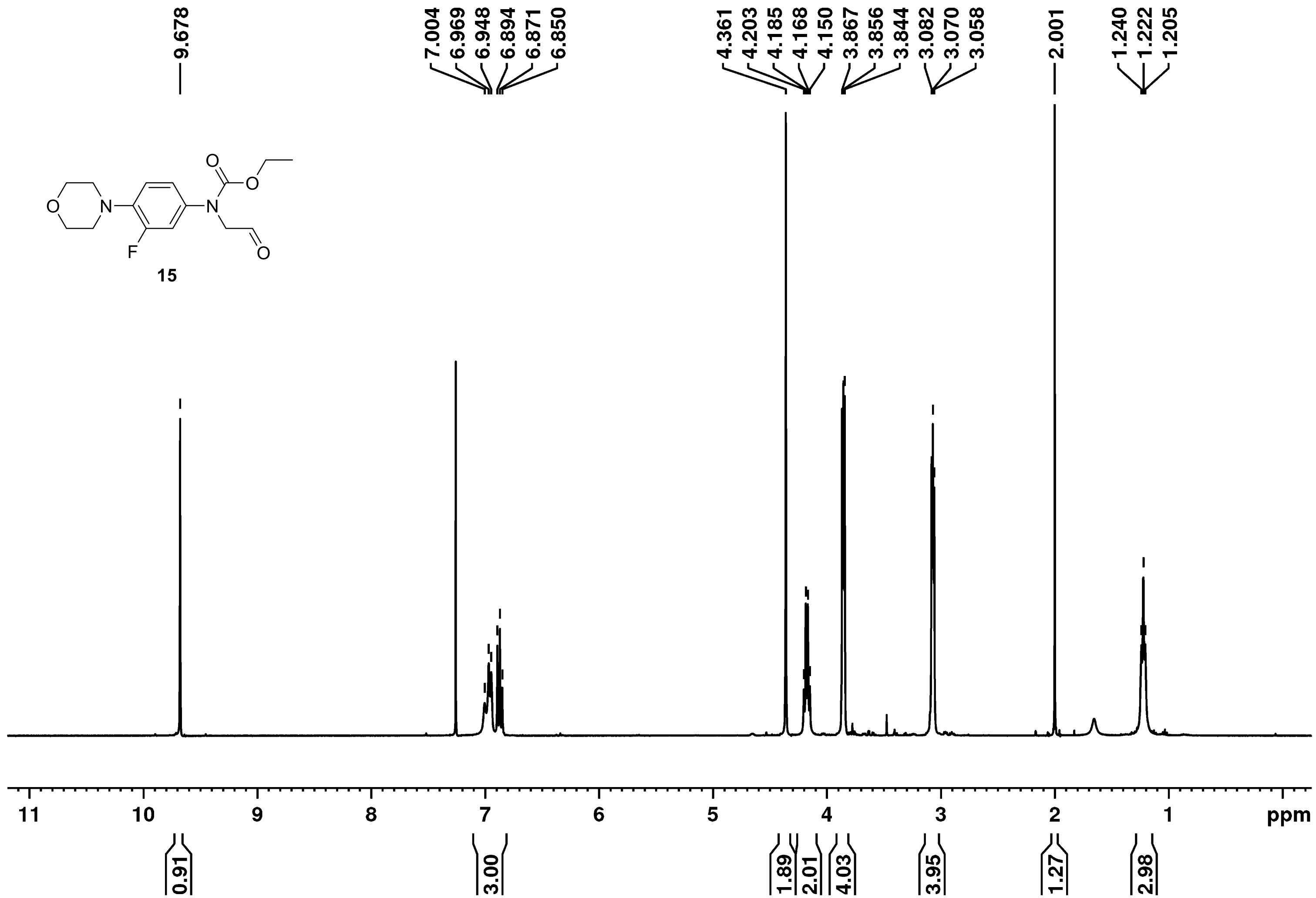
9.678
7.004
6.969
6.948
6.894
6.871
6.850
4.361
4.203
4.185
4.168
4.150
3.867
3.856
3.844
3.082
3.070
3.058
2.001
1.240
1.222
1.205
15
11
10
9
8
7
6
5
4
3
2
1
ppm
0.91
3.00
1.89
2.01
4.03
3.95
1.27
2.98

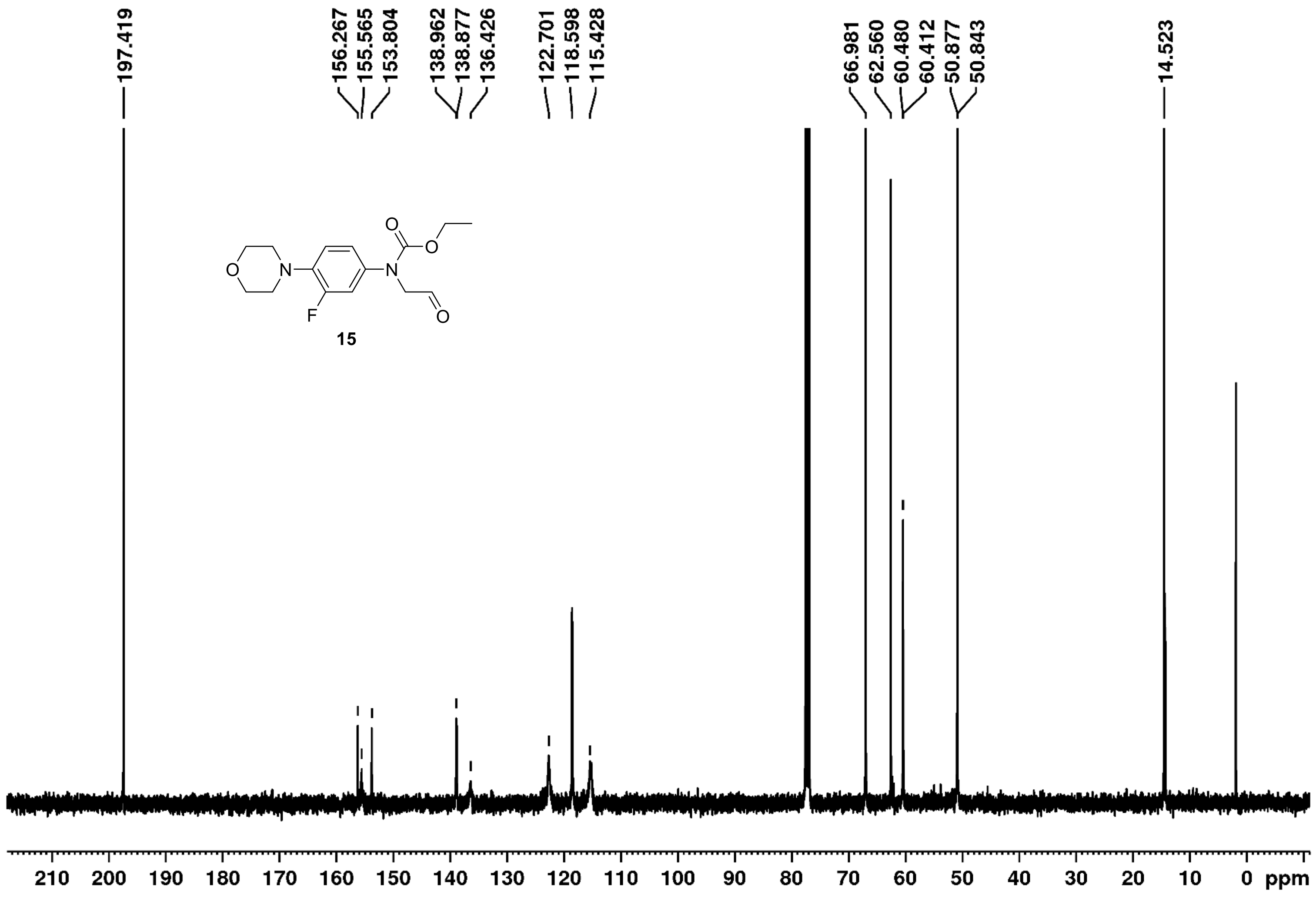
197.419
156.267
155.565
153.804
138.962
138.877
136.426
122.701
118.598
115.428
66.981
62.560
60.480
60.412
50.877
50.843
14.523
O
N
F
O
O
N
O
15
210 200 190 180 170 160 150 140 130 120 110 100 90 80 70 60 50 40 30 20 10 0 ppm

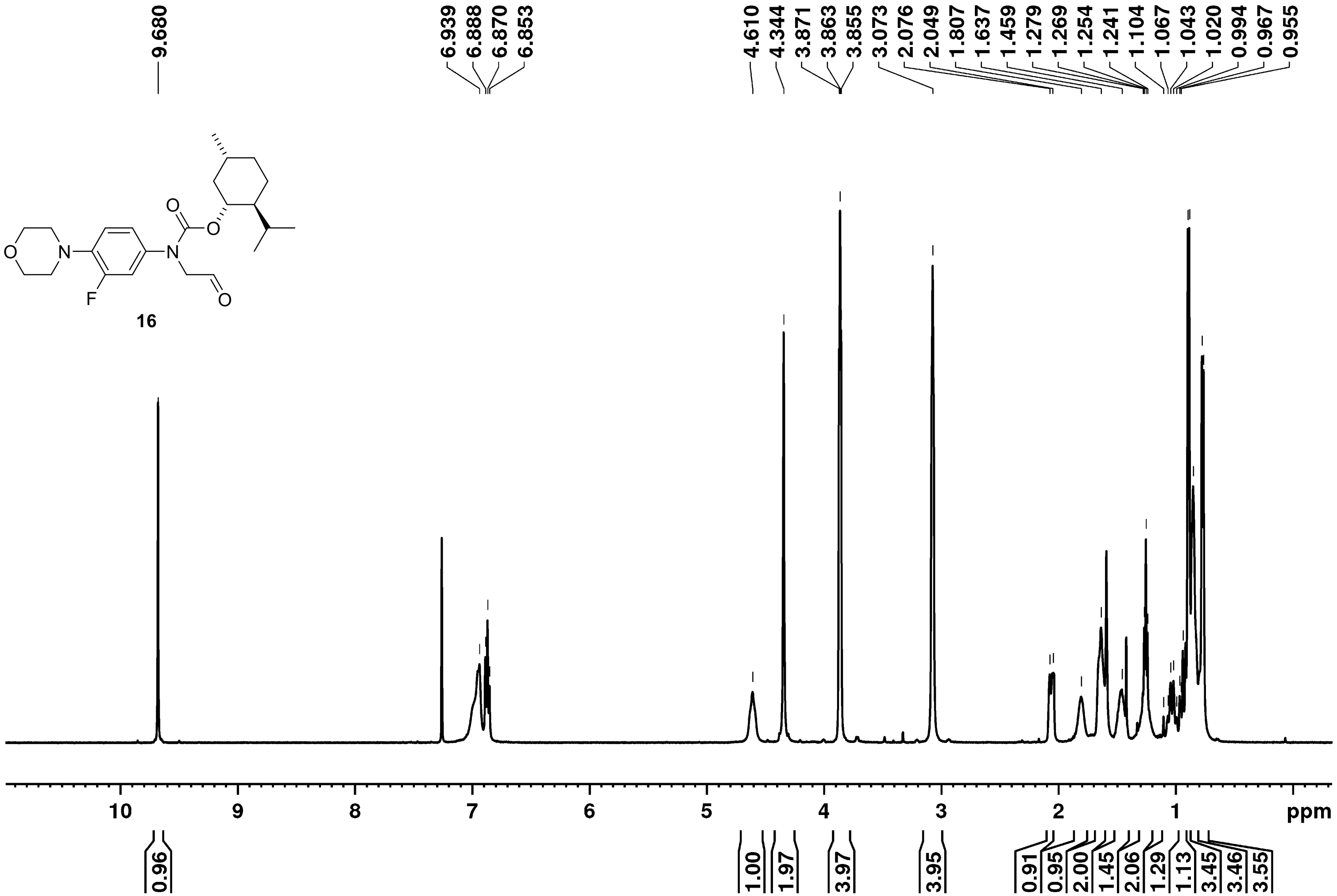

16

9.680
6.939
6.888
6.870
6.853
4.610
4.344
3.871
3.863
3.855
3.073
2.076
2.049
1.807
1.637
1.459
1.279
1.269
1.254
1.241
1.104
1.067
1.043
1.020
0.994
0.967
0.955
10
9
8
7
6
5
4
3
2
1
ppm
0.96
1.00
1.97
3.97
3.95
0.91
0.95
2.00
1.45
2.06
1.29
1.13
3.45
3.46
3.55

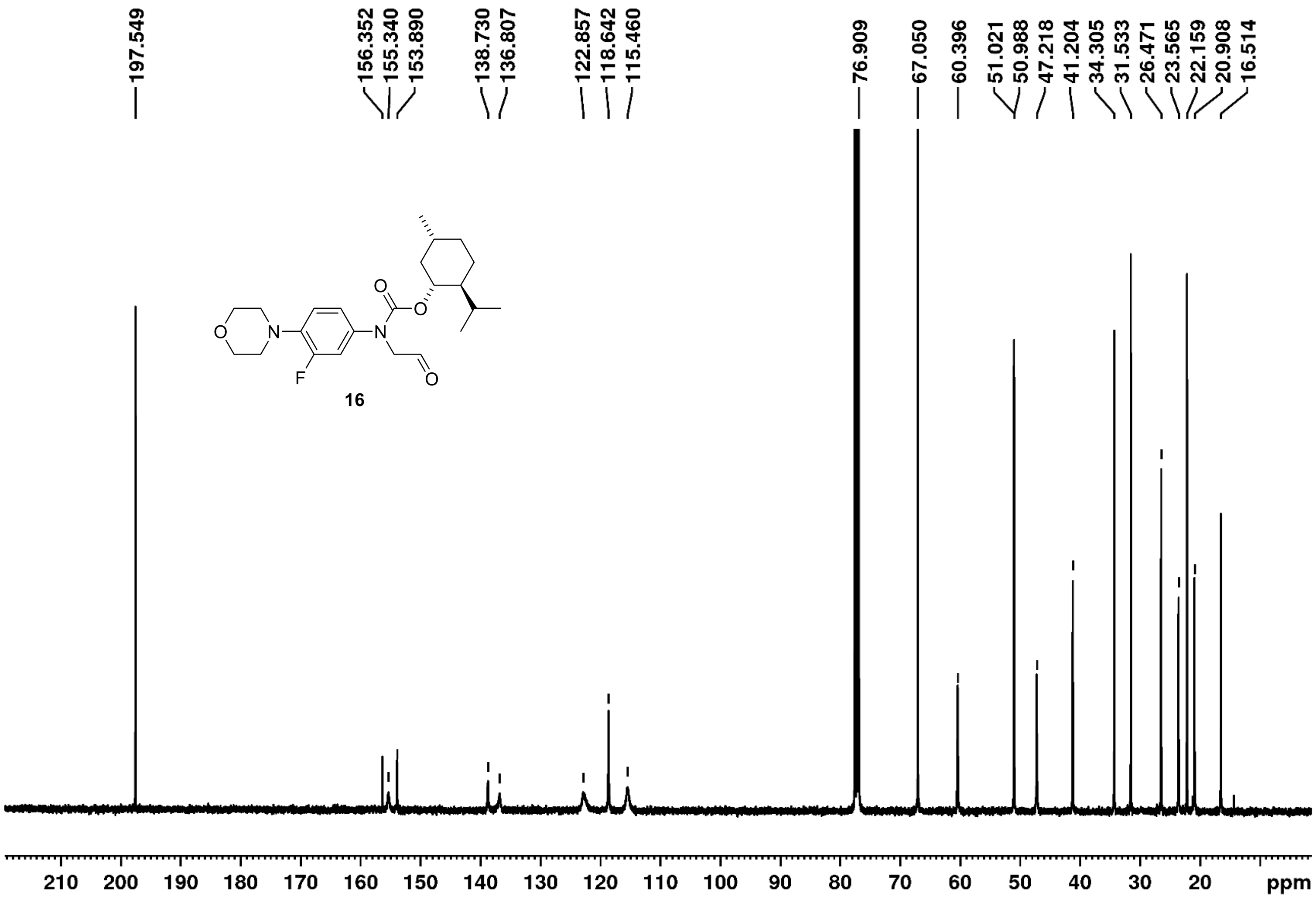
197.549
156.352
155.340
153.890
138.730
136.807
122.857
118.642
115.460
76.909
67.050
60.396
51.021
50.988
47.218
41.204
34.305
31.533
26.471
23.565
22.159
20.908
16.514
16
210 200 190 180 170 160 150 140 130 120 110 100 90 80 70 60 50 40 30 20 ppm

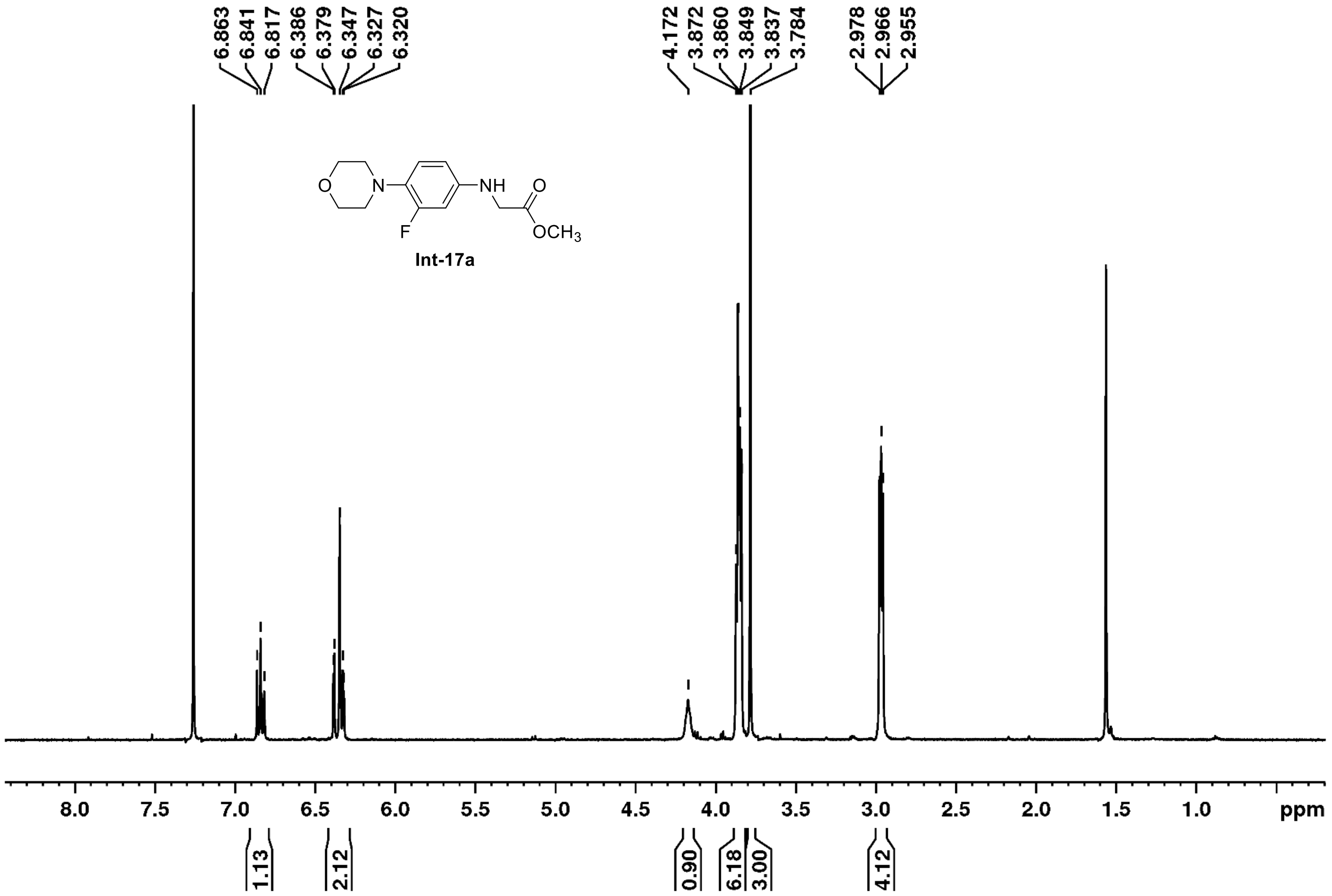
6.863
6.841
6.817
6.386
6.379
6.347
6.327
6.320
4.172
3.872
3.860
3.849
3.837
3.784
2.978
2.966
2.955
NH
O
N
F
OCH3
Int-17a
8.0
7.5
7.0
6.5
6.0
5.5
5.0
4.5
4.0
3.5
3.0
2.5
2.0
1.5
1.0
ppm
1.13
2.12
0.90
6.18
3.00
4.12

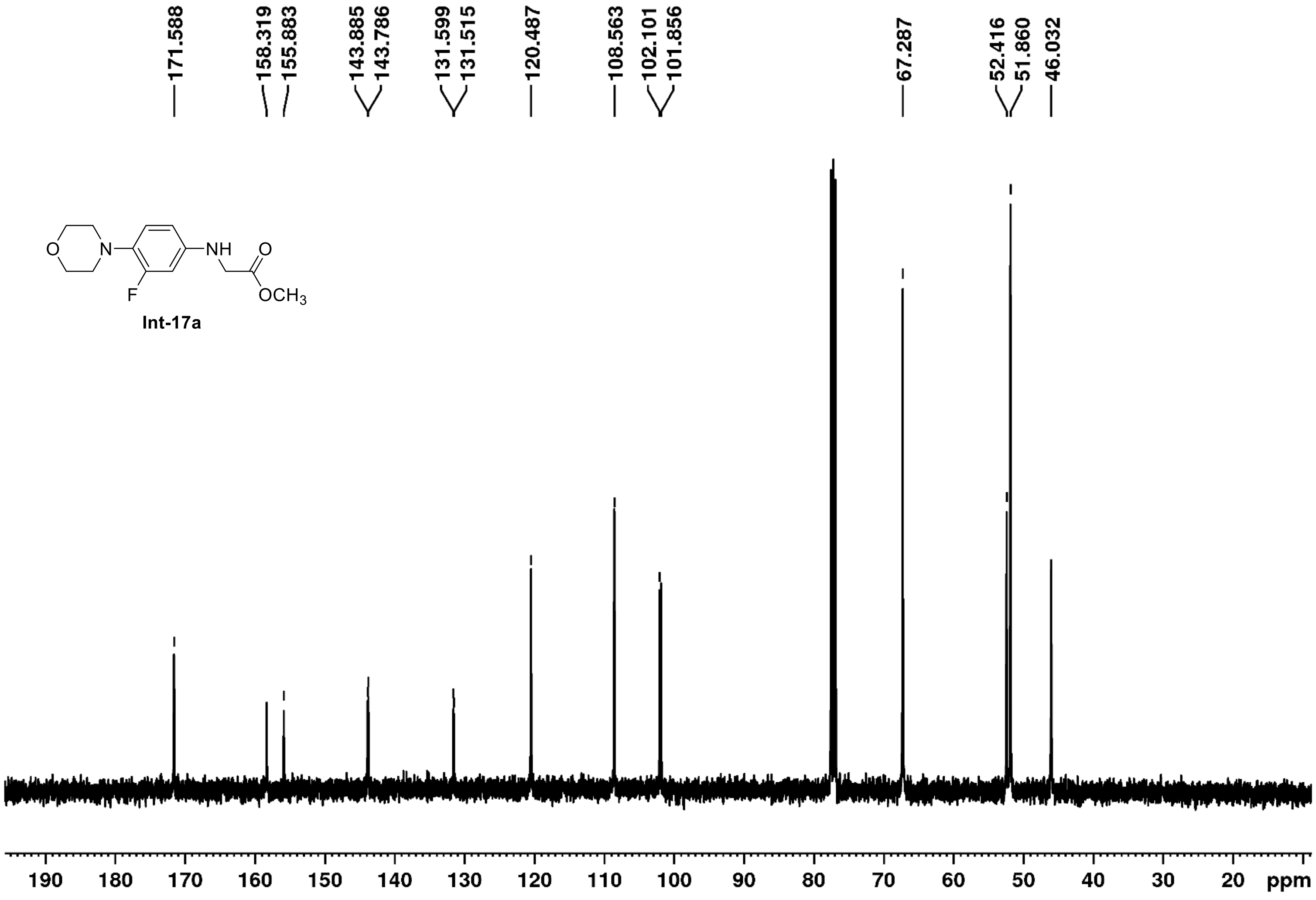

171.588
158.319
155.883
143.885
143.786
131.599
131.515
120.487
108.563
102.101
101.856
67.287
52.416
51.860
46.032
O
N
NH
O
F
OCH3
Int-17a
190 180 170 160 150 140 130 120 110 100 90 80 70 60 50 40 30 20 ppm

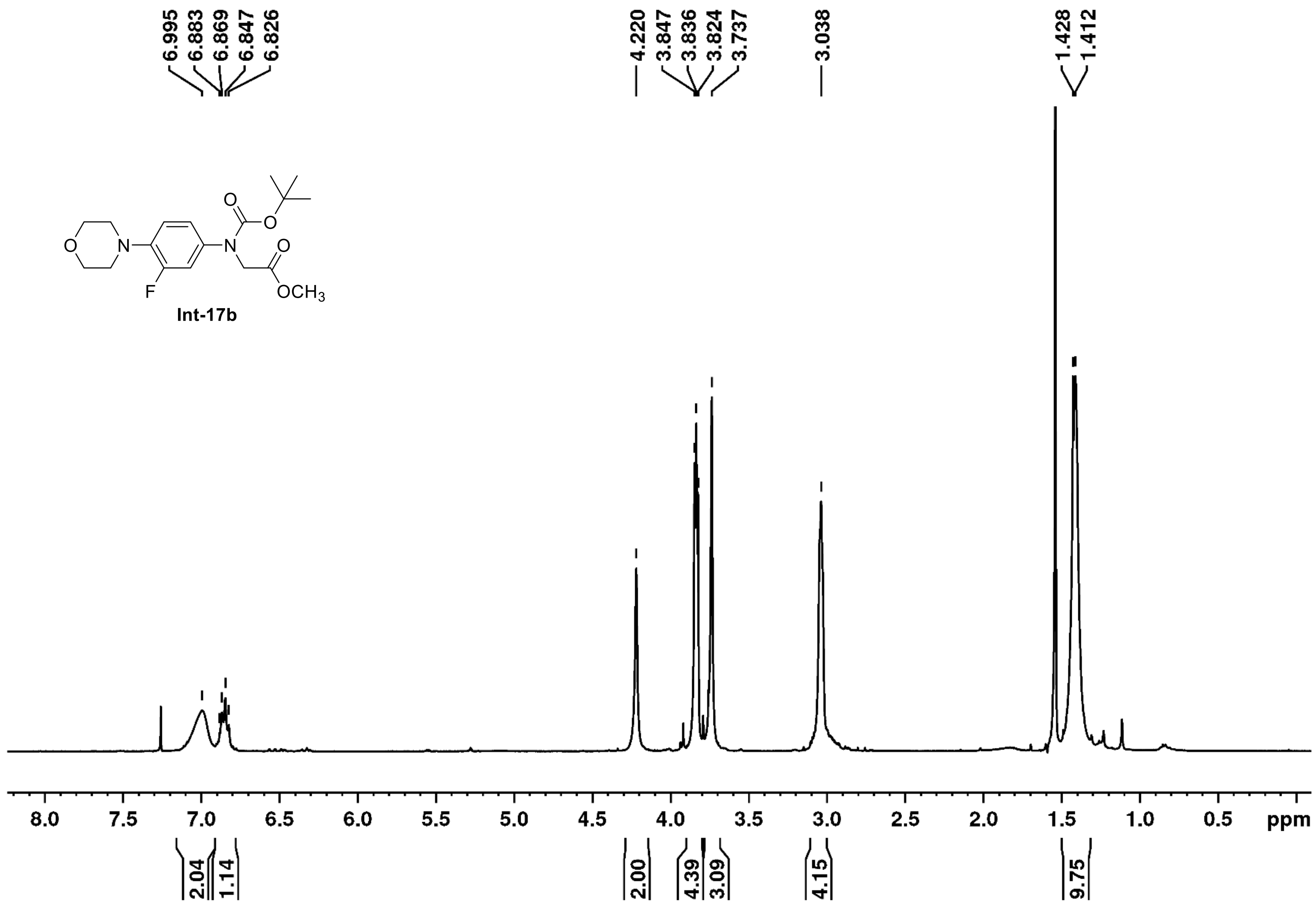
6.995
6.883
6.869
6.847
6.826
4.220
3.847
3.836
3.824
3.737
3.038
1.428
1.412
O
N
F
N
O
O
O
OCH3
Int-17b
8.0
7.5
7.0
6.5
6.0
5.5
5.0
4.5
4.0
3.5
3.0
2.5
2.0
1.5
1.0
0.5
ppm
2.04
1.14
2.00
4.39
3.09
4.15
9.75

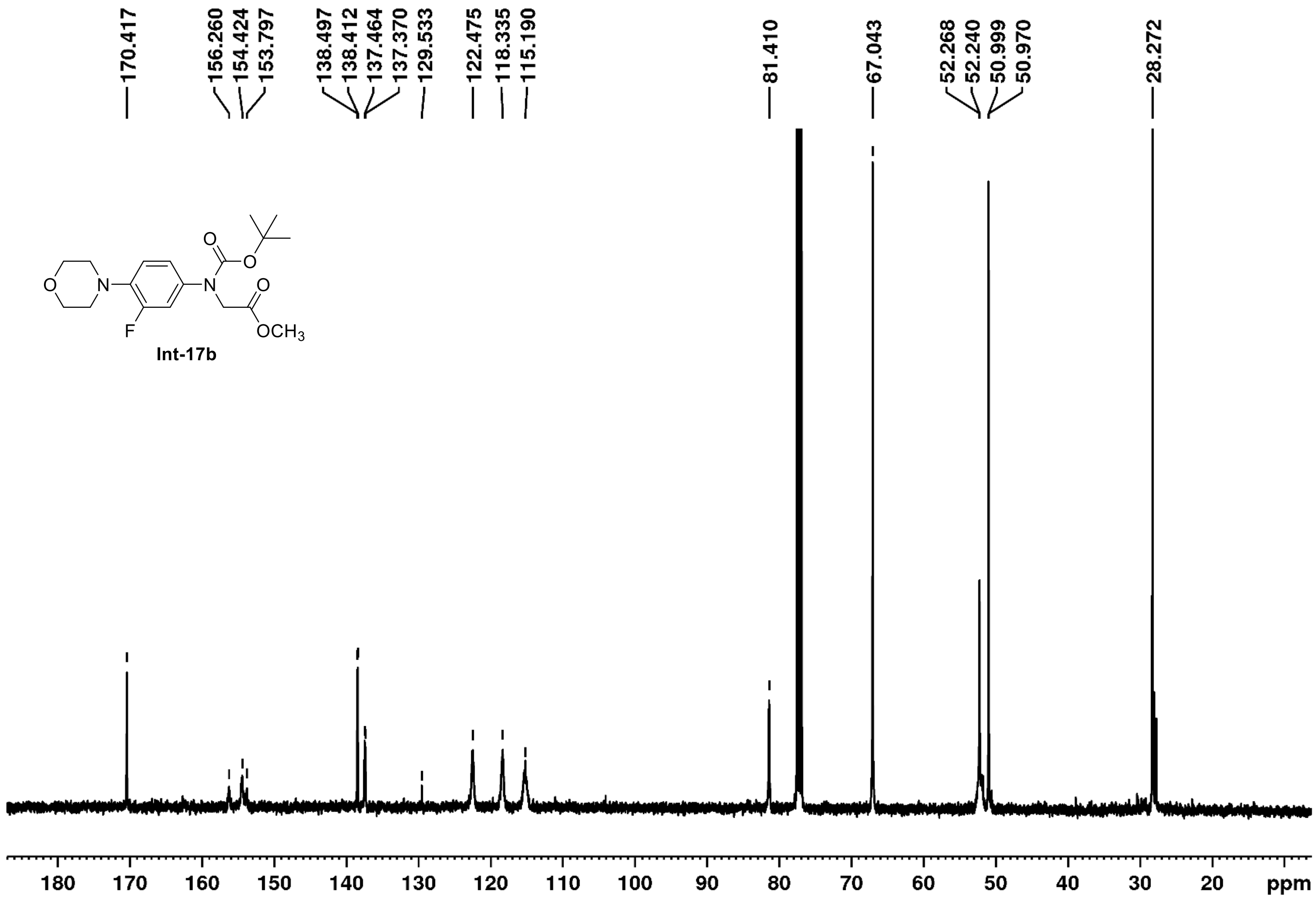
170.417
156.260
154.424
153.797
138.497
138.412
137.464
137.370
129.533
122.475
118.335
115.190
81.410
67.043
52.268
52.240
50.999
50.970
28.272
O
N
F
N
O
O
O
OCH3
Int-17b
180
170
160
150
140
130
120
110
100
90
80
70
60
50
40
30
20
ppm

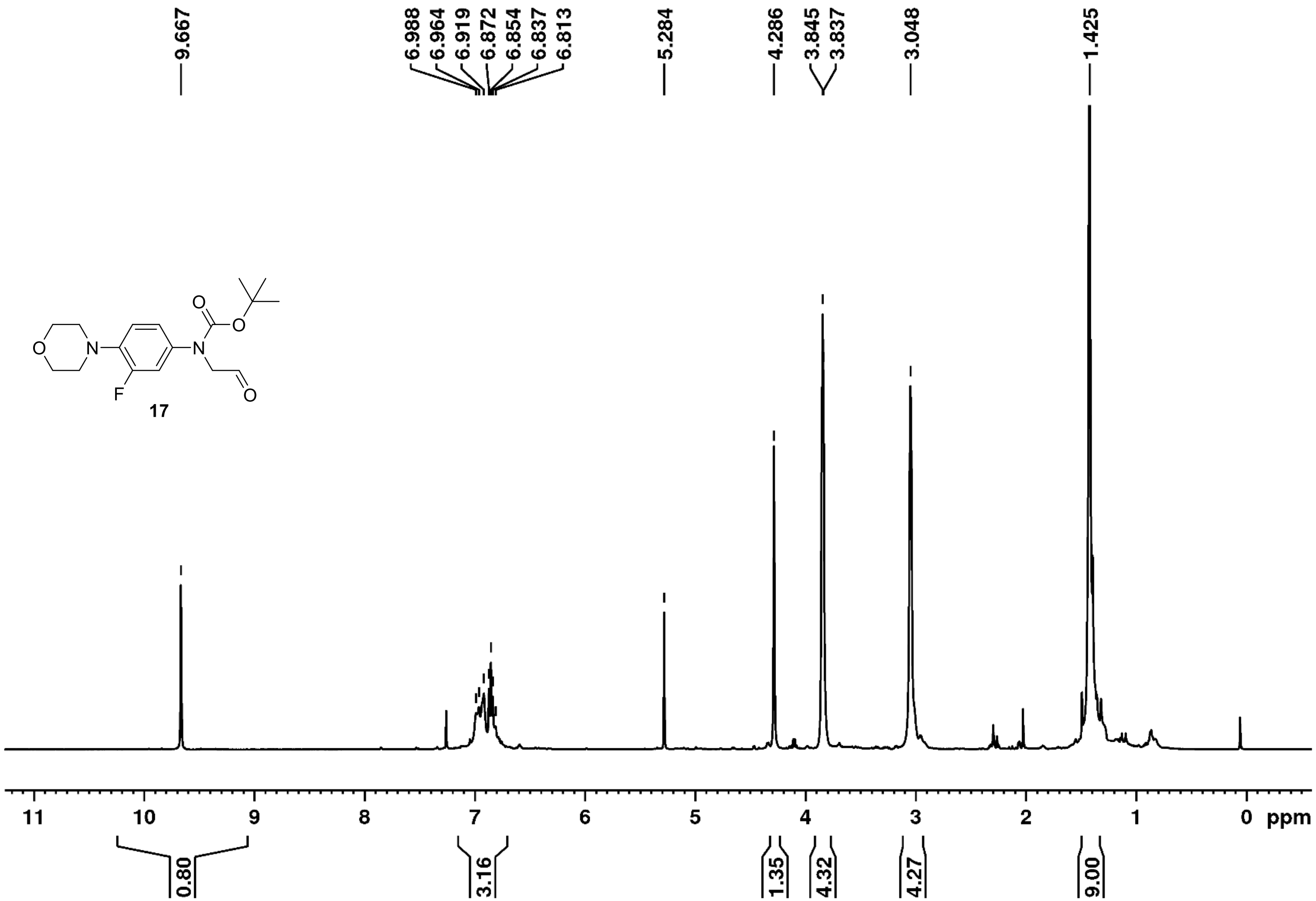
9.667
6.988
6.964
6.919
6.872
6.854
6.837
6.813
5.284
4.286
3.845
3.837
3.048
1.425
O
N
N
O
O
F
O
17
11
10
9
8
7
6
5
4
3
2
1
0
ppm
0.80
3.16
1.35
4.32
4.27
9.00

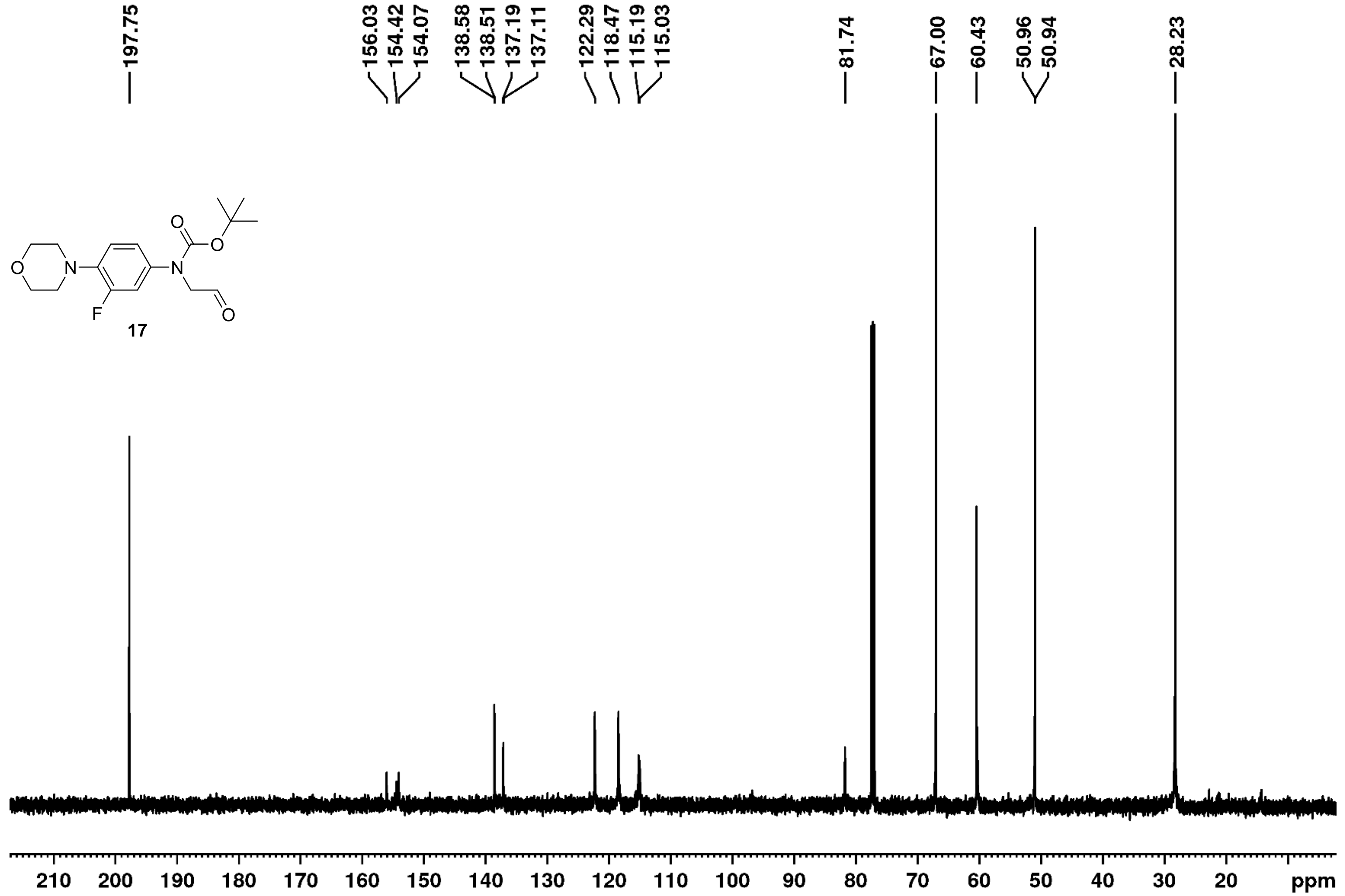
197.75
156.03
154.42
154.07
138.58
138.51
137.19
137.11
122.29
118.47
115.19
115.03
81.74
67.00
60.43
50.96
50.94
28.23
17
210 200 190 180 170 160 150 140 130 120 110 100 90 80 70 60 50 40 30 20 ppm

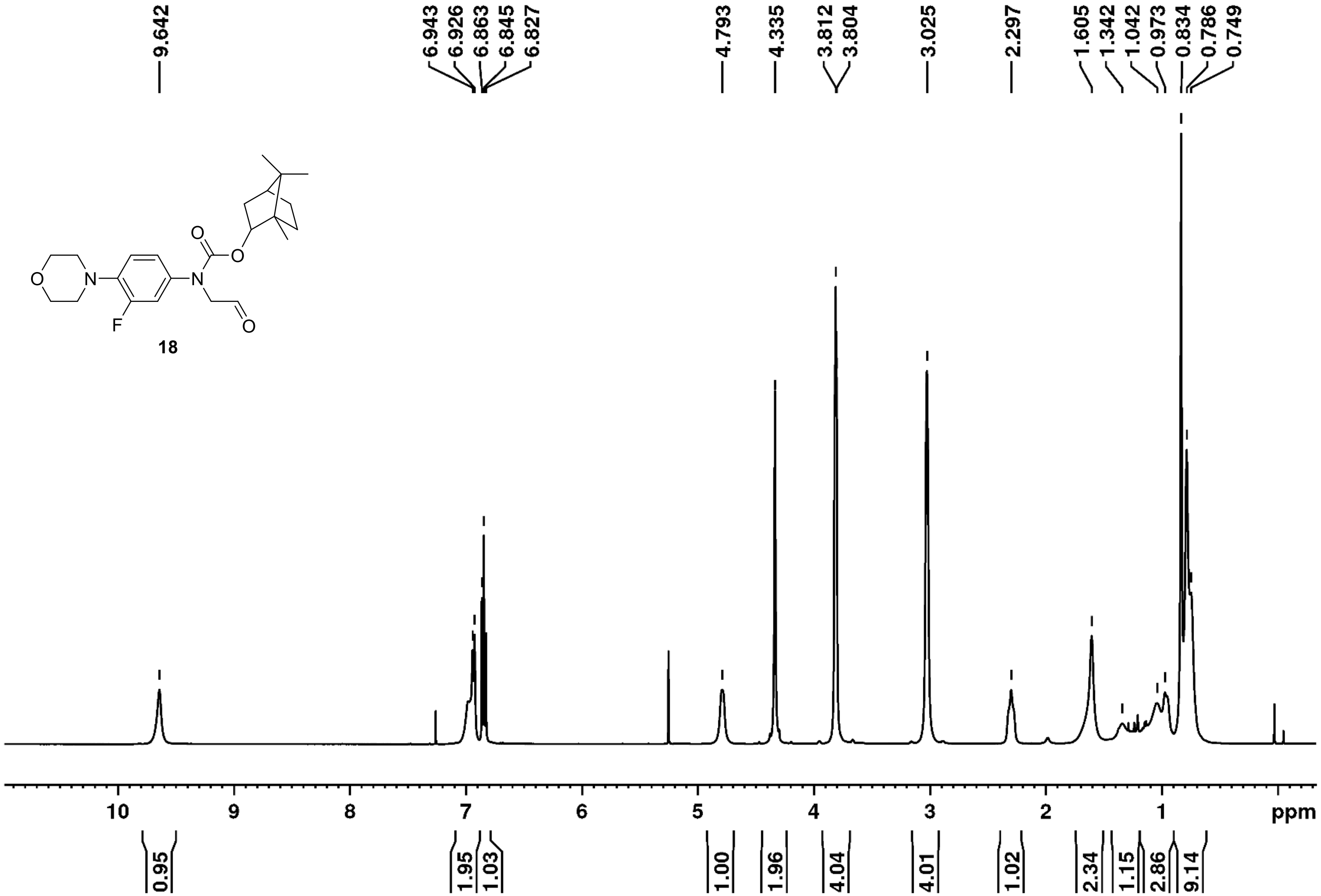
9.642
6.943
6.926
6.863
6.845
6.827
4.793
4.335
3.812
3.804
3.025
2.297
1.605
1.342
1.042
0.973
0.834
0.786
0.749
O
N
F
N
O
O
O
18
10
9
8
7
6
5
4
3
2
1
ppm
0.95
1.95
1.03
1.00
1.96
4.04
4.01
1.02
2.34
1.15
2.86
9.14

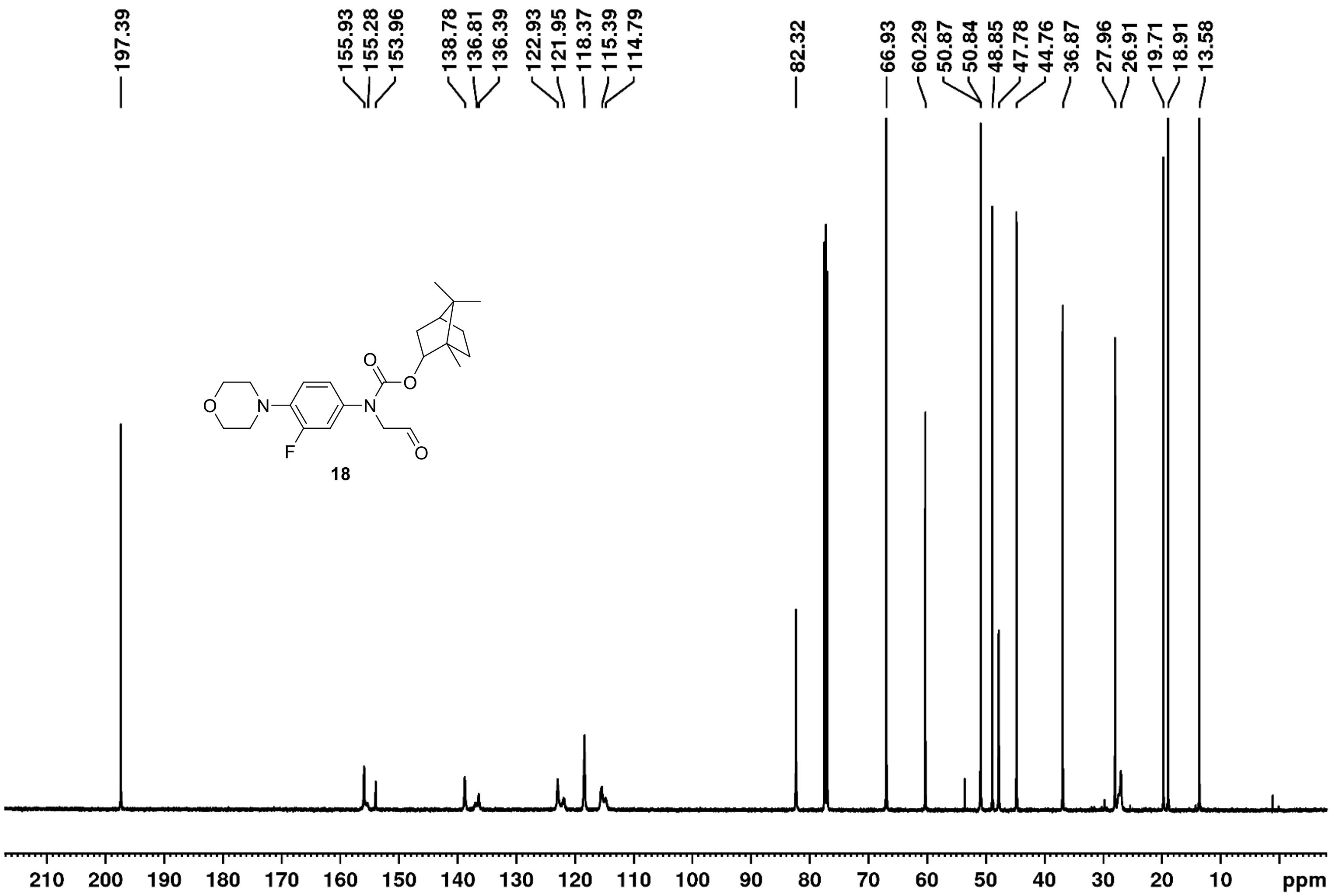
197.39
155.93
155.28
153.96
138.78
136.81
136.39
122.93
121.95
118.37
115.39
114.79
82.32
66.93
60.29
50.87
50.84
48.85
47.78
44.76
36.87
27.96
26.91
19.71
18.91
13.58
O
N
F
18
210 200 190 180 170 160 150 140 130 120 110 100 90 80 70 60 50 40 30 20 10 ppm

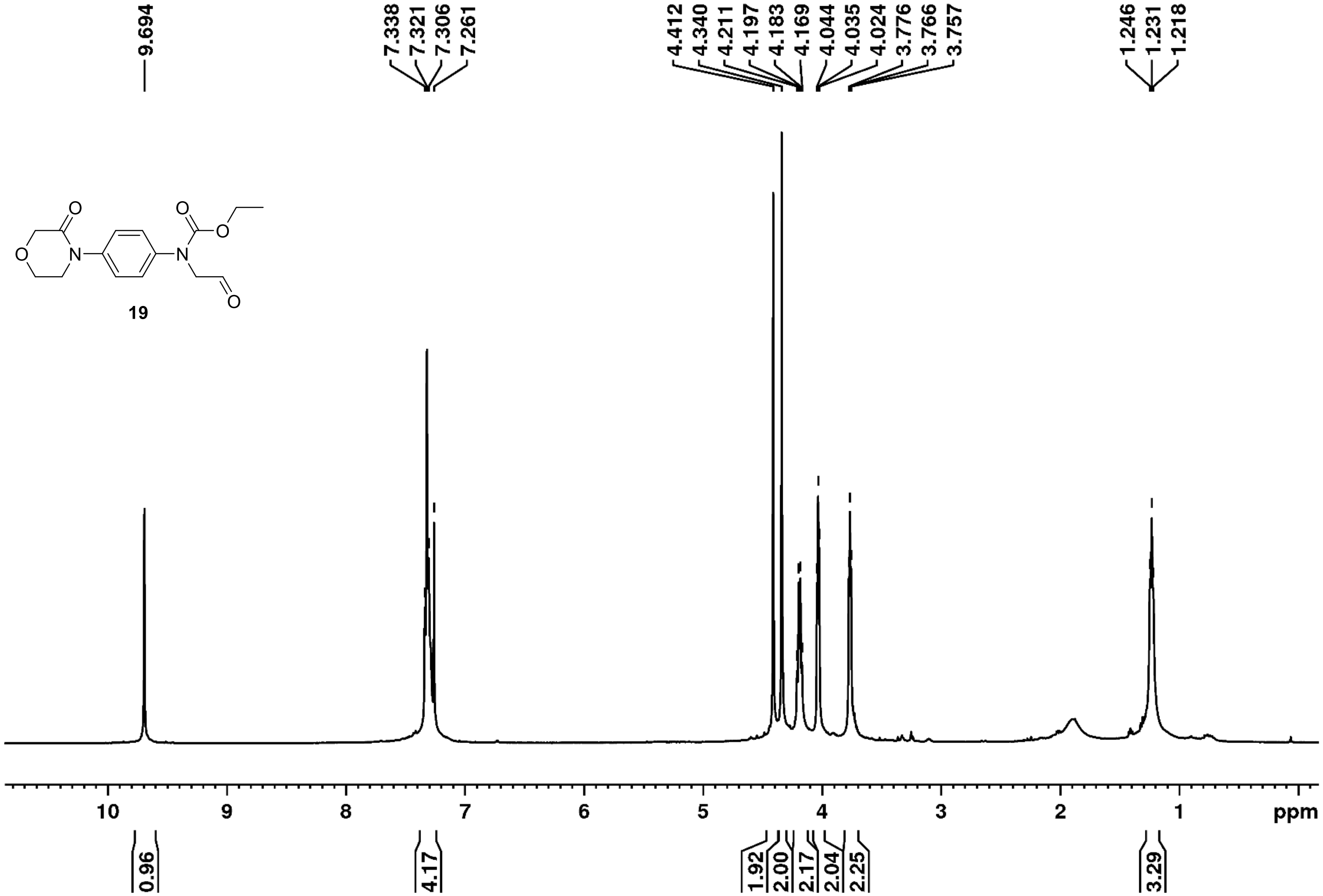
9.694
7.338
7.321
7.306
7.261
4.412
4.340
4.211
4.197
4.183
4.169
4.044
4.035
4.024
3.776
3.766
3.757
1.246
1.231
1.218
19
10
9
8
7
6
5
4
3
2
1
ppm
0.96
4.17
1.92
2.00
2.17
2.04
2.25
3.29

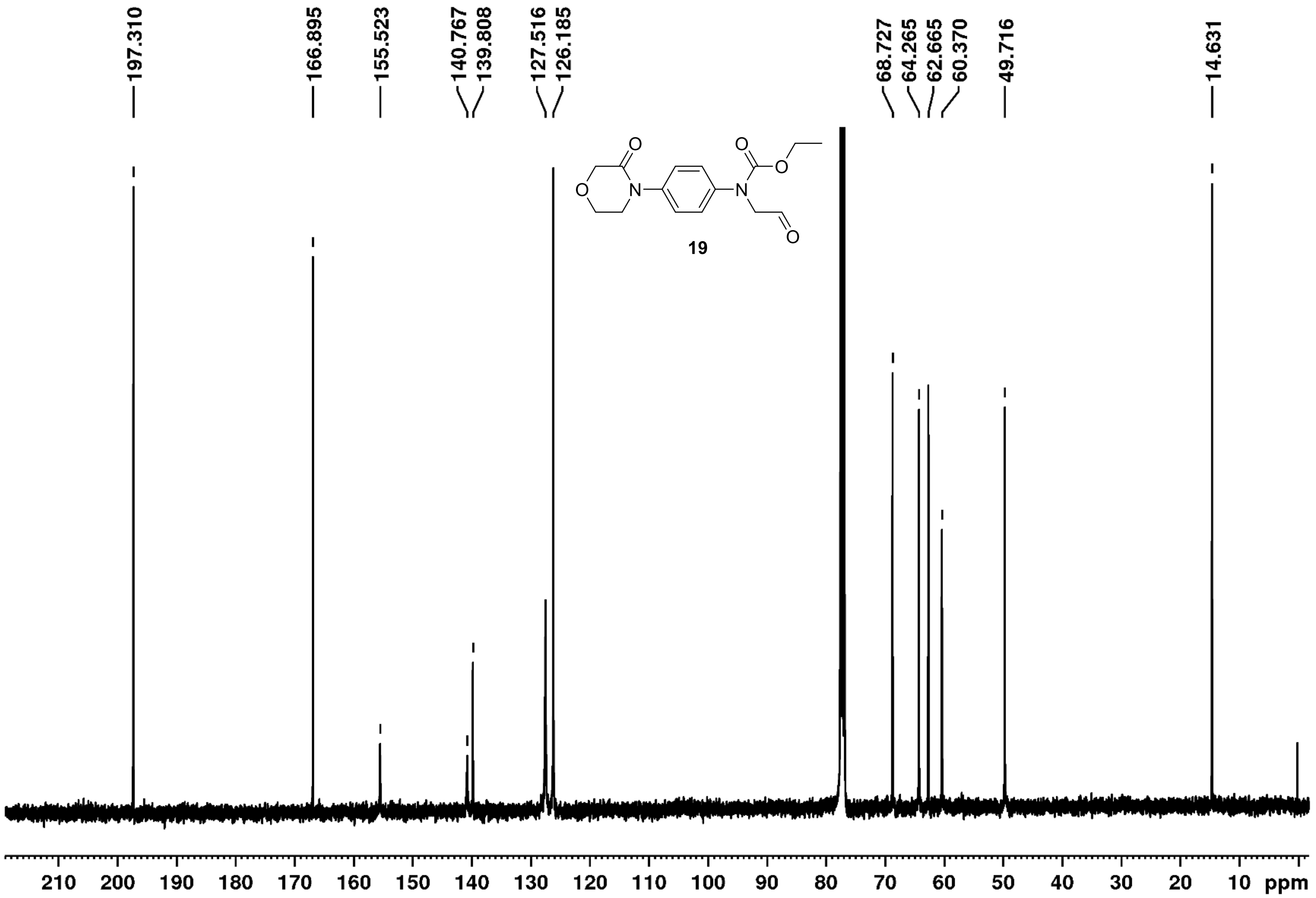
197.310
166.895
155.523
140.767
139.808
127.516
126.185
68.727
64.265
62.665
60.370
49.716
14.631
19
210 200 190 180 170 160 150 140 130 120 110 100 90 80 70 60 50 40 30 20 10 ppm

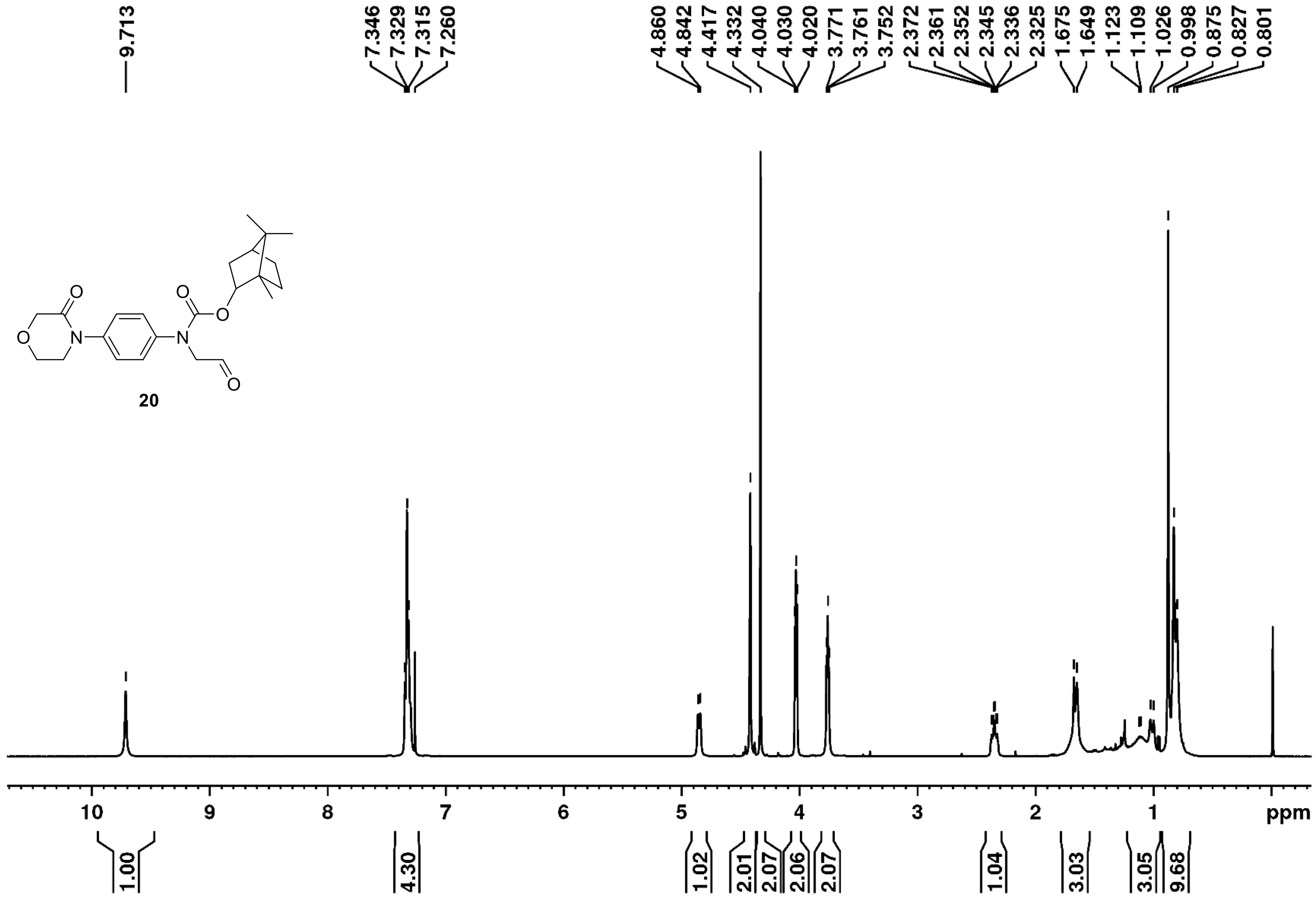
9.713
7.346
7.329
7.315
7.260
4.860
4.842
4.417
4.332
4.040
4.030
4.020
3.771
3.761
3.752
2.372
2.361
2.352
2.345
2.336
2.325
1.675
1.649
1.123
1.109
1.026
0.998
0.875
0.827
0.801
10
9
8
7
6
5
4
3
2
1
ppm
1.00
4.30
1.02
2.01
2.07
2.06
2.07
1.04
3.03
3.05
9.68

20

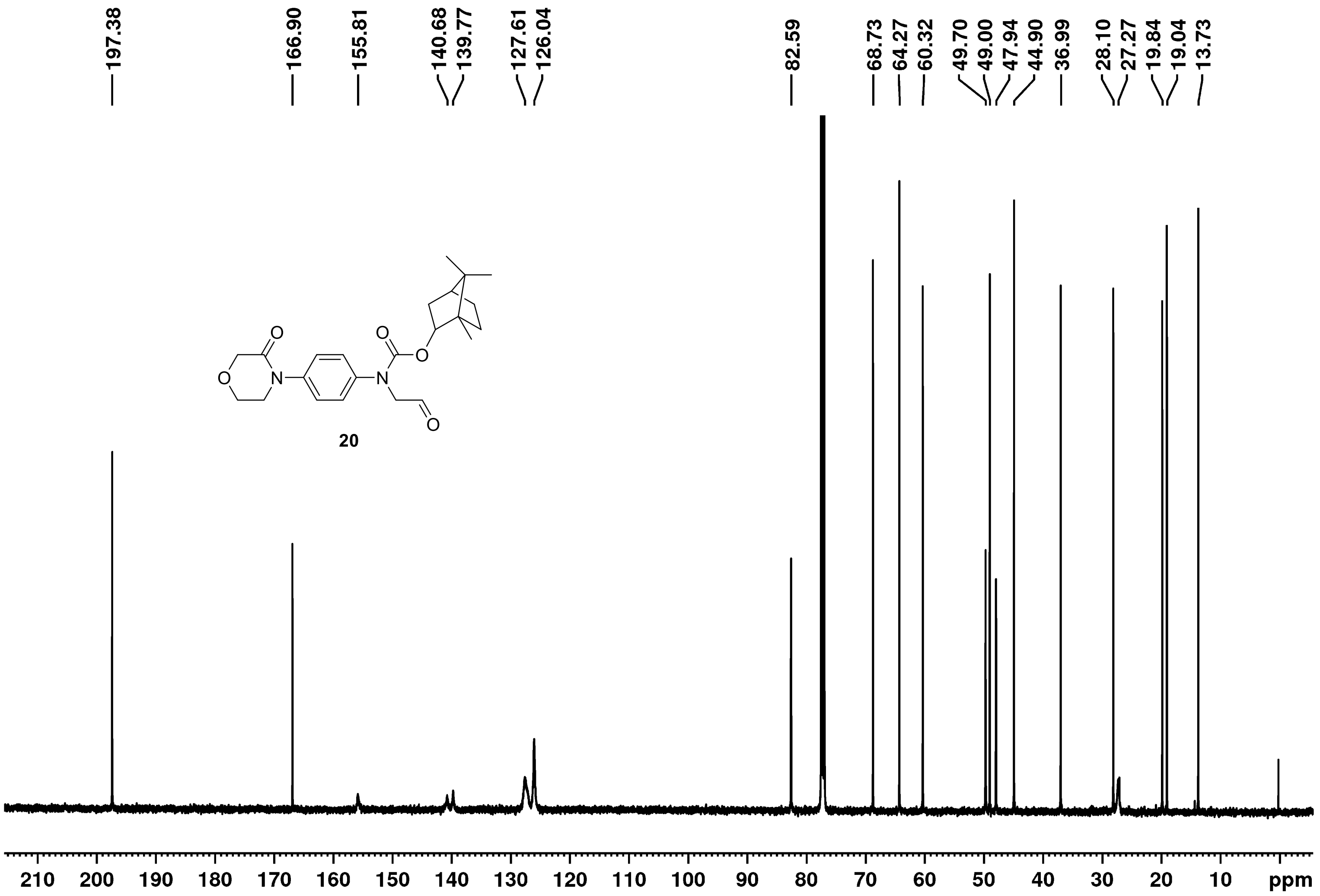
197.38
166.90
155.81
140.68
139.77
127.61
126.04
82.59
68.73
64.27
60.32
49.70
49.00
47.94
44.90
36.99
28.10
27.27
19.84
19.04
13.73
20
210 200 190 180 170 160 150 140 130 120 110 100 90 80 70 60 50 40 30 20 10 ppm

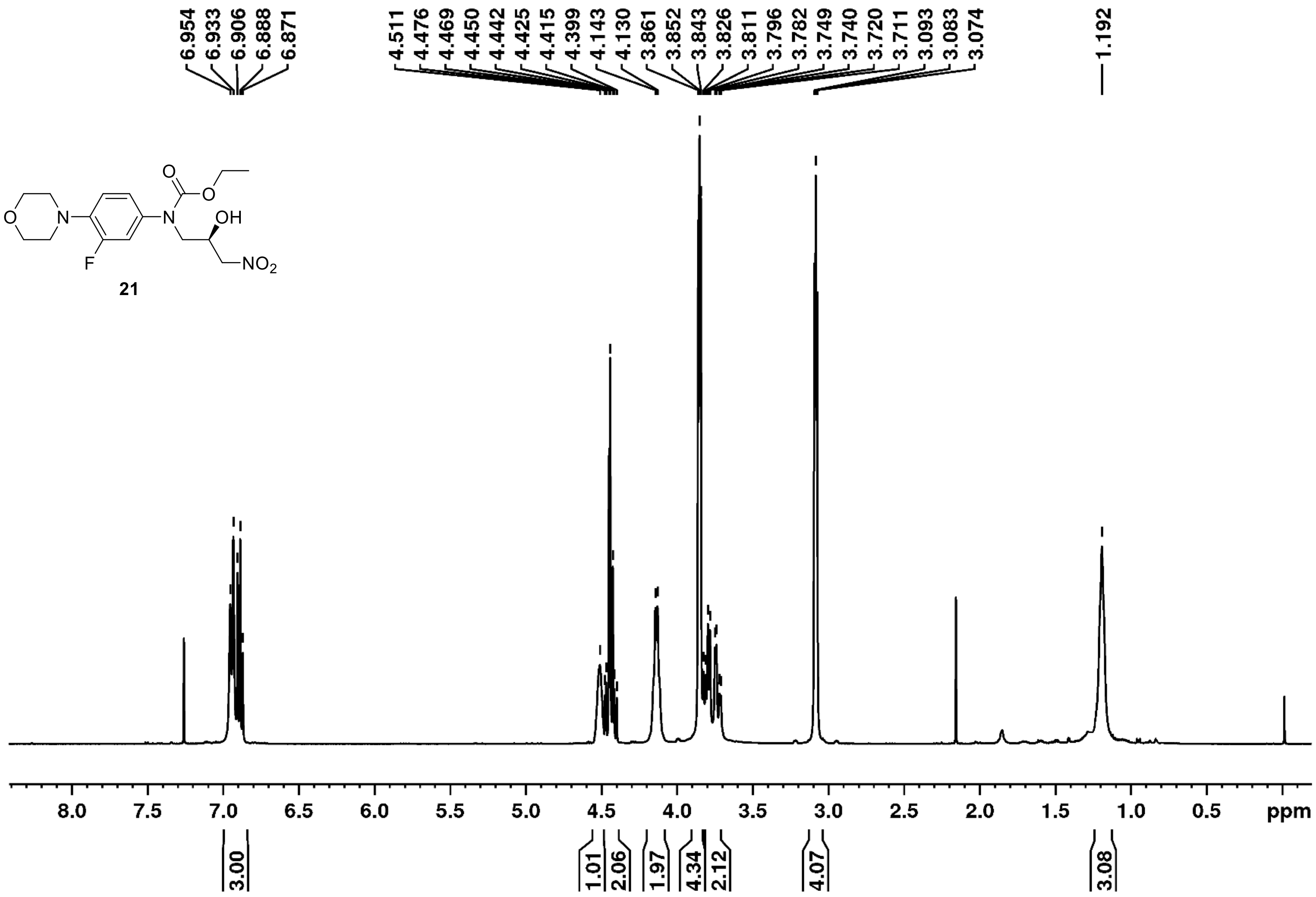
6.954
6.933
6.906
6.888
6.871
4.511
4.476
4.469
4.450
4.442
4.425
4.415
4.399
4.143
4.130
3.861
3.852
3.843
3.826
3.811
3.796
3.782
3.749
3.740
3.720
3.711
3.093
3.083
3.074
1.192
O
N
F
O
O
N
OH
NO2
21
8.0
7.5
7.0
6.5
6.0
5.5
5.0
4.5
4.0
3.5
3.0
2.5
2.0
1.5
1.0
0.5
ppm
3.00
1.01
2.06
1.97
4.34
2.12
4.07
3.08

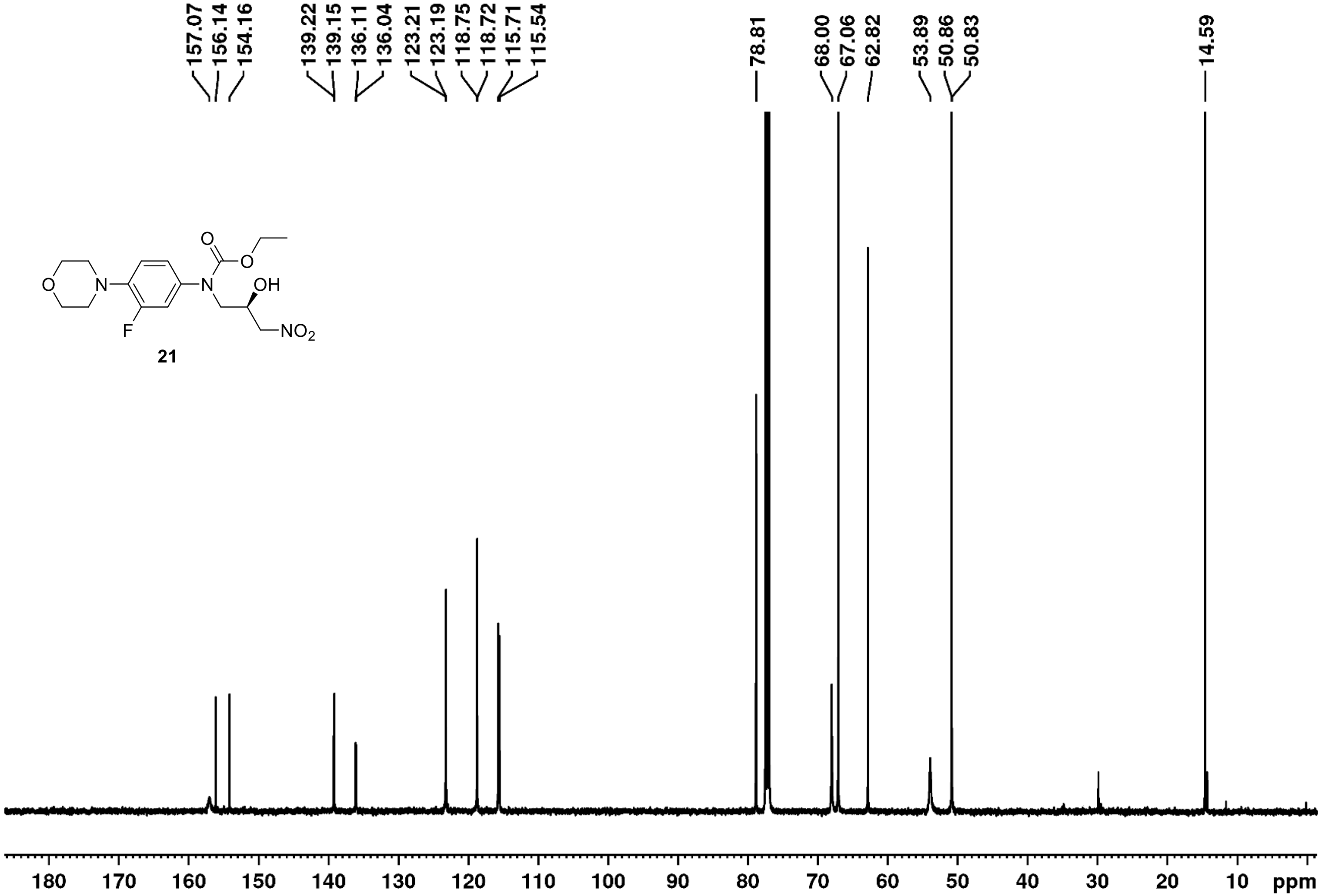
157.07
156.14
154.16
139.22
139.15
136.11
136.04
123.21
123.19
118.75
118.72
115.71
115.54
78.81
68.00
67.06
62.82
53.89
50.86
50.83
14.59
O
N
F
N
O
O
OH
NO2
21
180 170 160 150 140 130 120 110 100 90 80 70 60 50 40 30 20 10 ppm

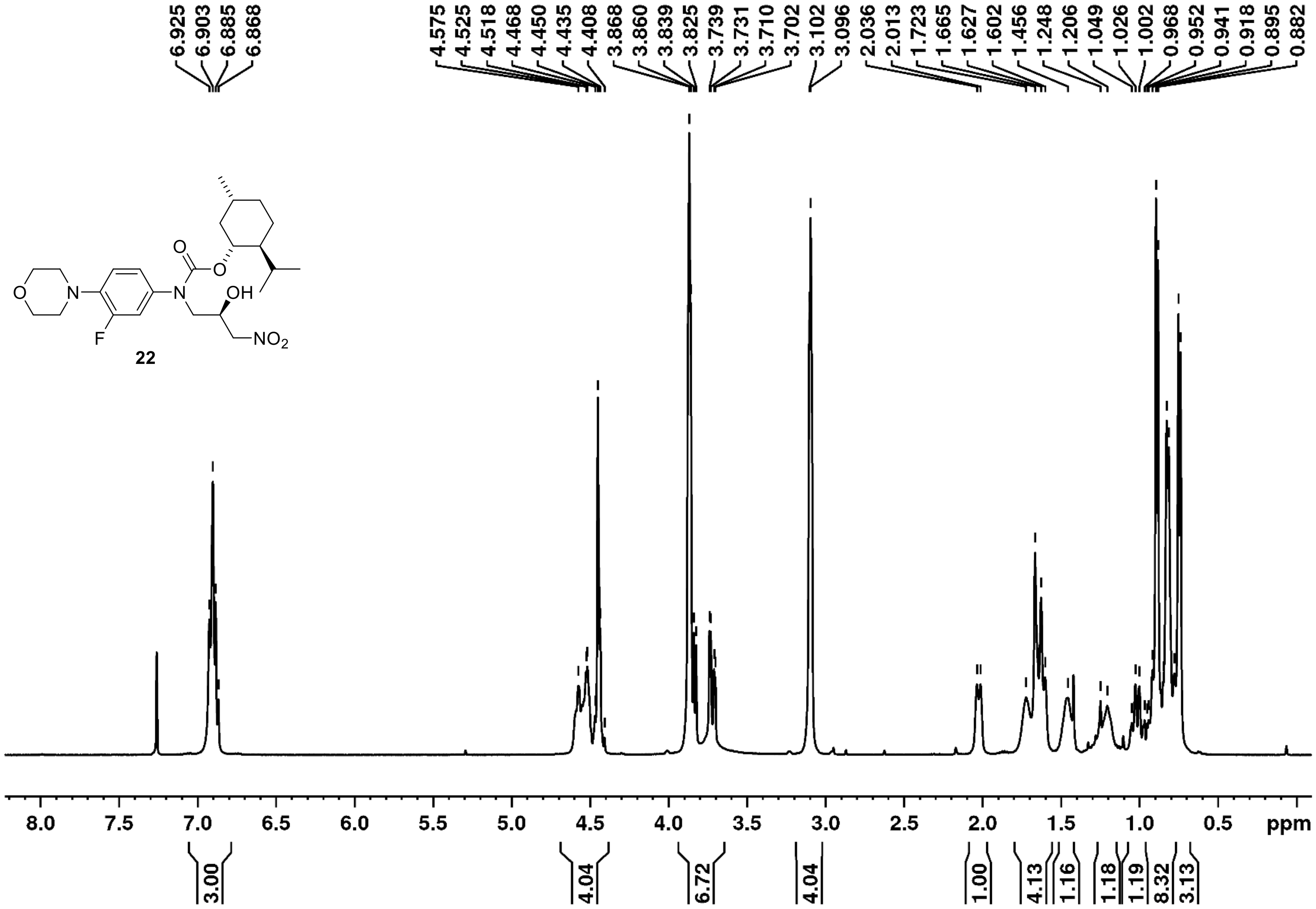
6.925
6.903
6.885
6.868
4.575
4.525
4.518
4.468
4.450
4.435
4.408
3.868
3.860
3.839
3.825
3.739
3.731
3.710
3.702
3.102
3.096
2.036
2.013
1.723
1.665
1.627
1.602
1.456
1.248
1.206
1.049
1.026
1.002
0.968
0.952
0.941
0.918
0.895
0.882
O
N
F
N
O
O
OH
NO2
22
8.0 7.5 7.0 6.5 6.0 5.5 5.0 4.5 4.0 3.5 3.0 2.5 2.0 1.5 1.0 0.5 ppm
3.00
4.04
6.72
4.04
1.00
4.13
1.16
1.18
1.19
8.32
3.13

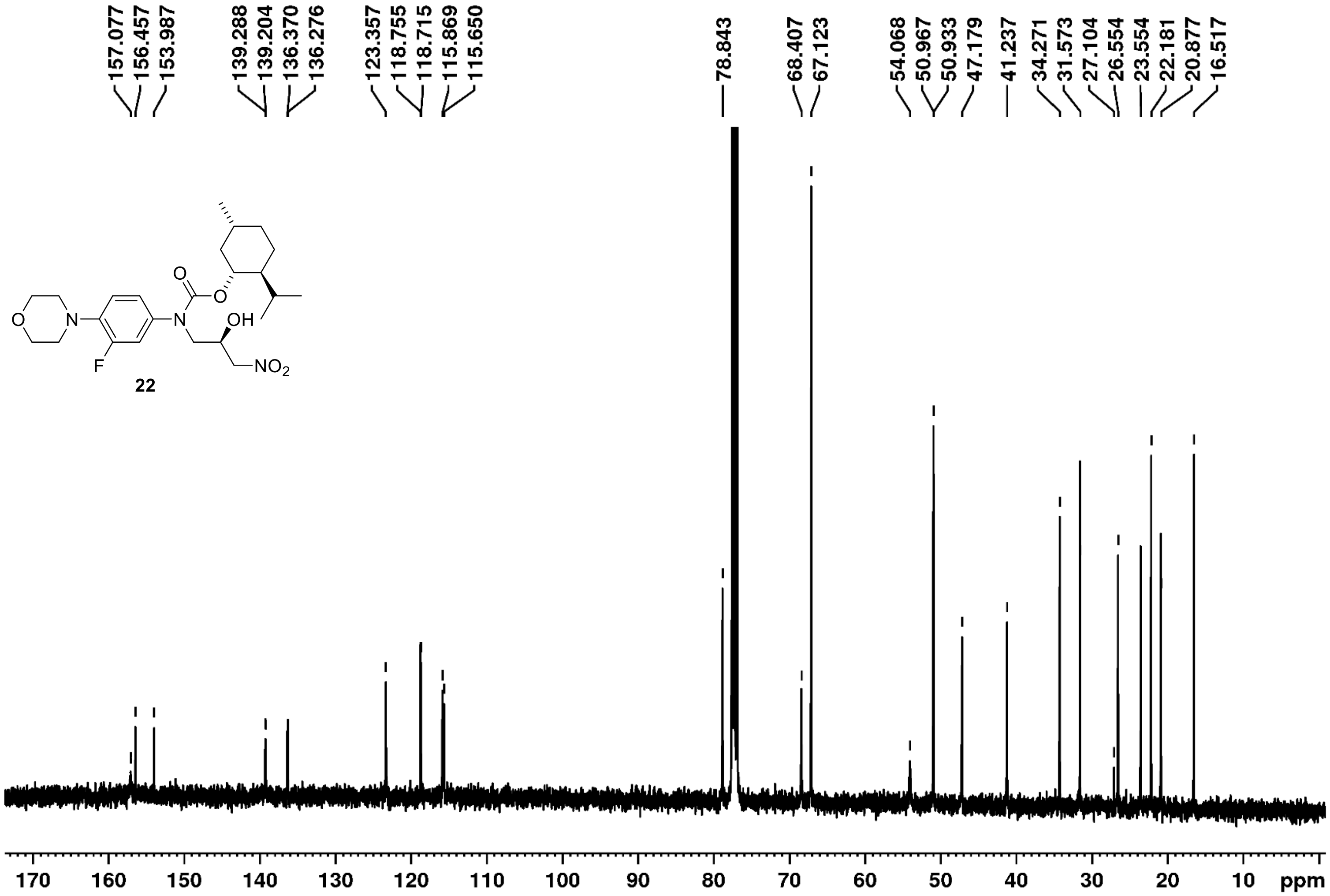
157.077
156.457
153.987
139.288
139.204
136.370
136.276
123.357
118.755
118.715
115.869
115.650
78.843
68.407
67.123
54.068
50.967
50.933
47.179
41.237
34.271
31.573
27.104
26.554
23.554
22.181
20.877
16.517
O
N
F
N
O
O
OH
NO_2
22
170 160 150 140 130 120 110 100 90 80 70 60 50 40 30 20 10 ppm

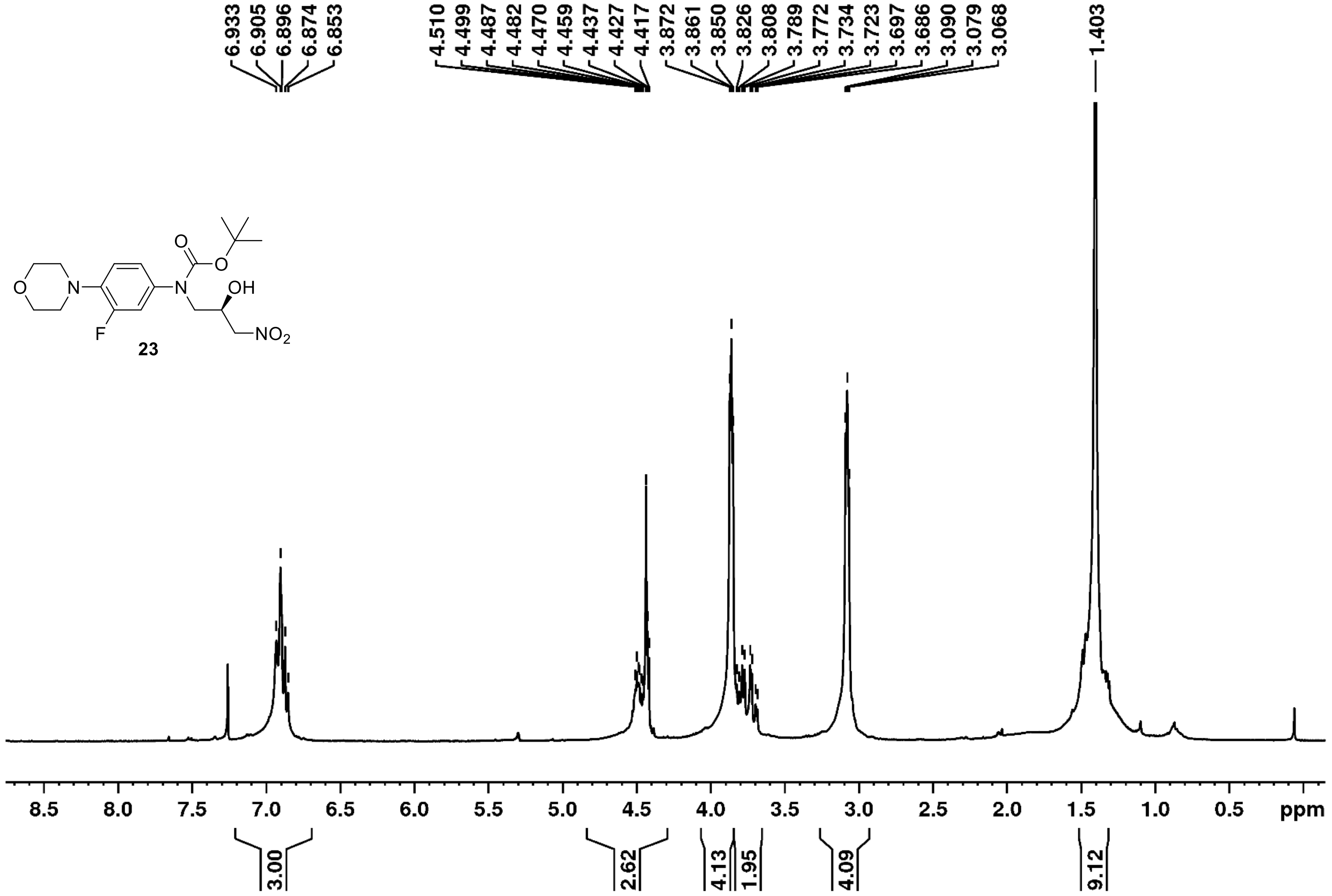
6.933
6.905
6.896
6.874
6.853
4.510
4.499
4.487
4.482
4.470
4.459
4.437
4.427
4.417
3.872
3.861
3.850
3.826
3.808
3.789
3.772
3.734
3.723
3.697
3.686
3.090
3.079
3.068
1.403
O
N
O
O
N
OH
F
NO2
23
8.5
8.0
7.5
7.0
6.5
6.0
5.5
5.0
4.5
4.0
3.5
3.0
2.5
2.0
1.5
1.0
0.5
ppm
3.00
2.62
4.13
1.95
4.09
9.12

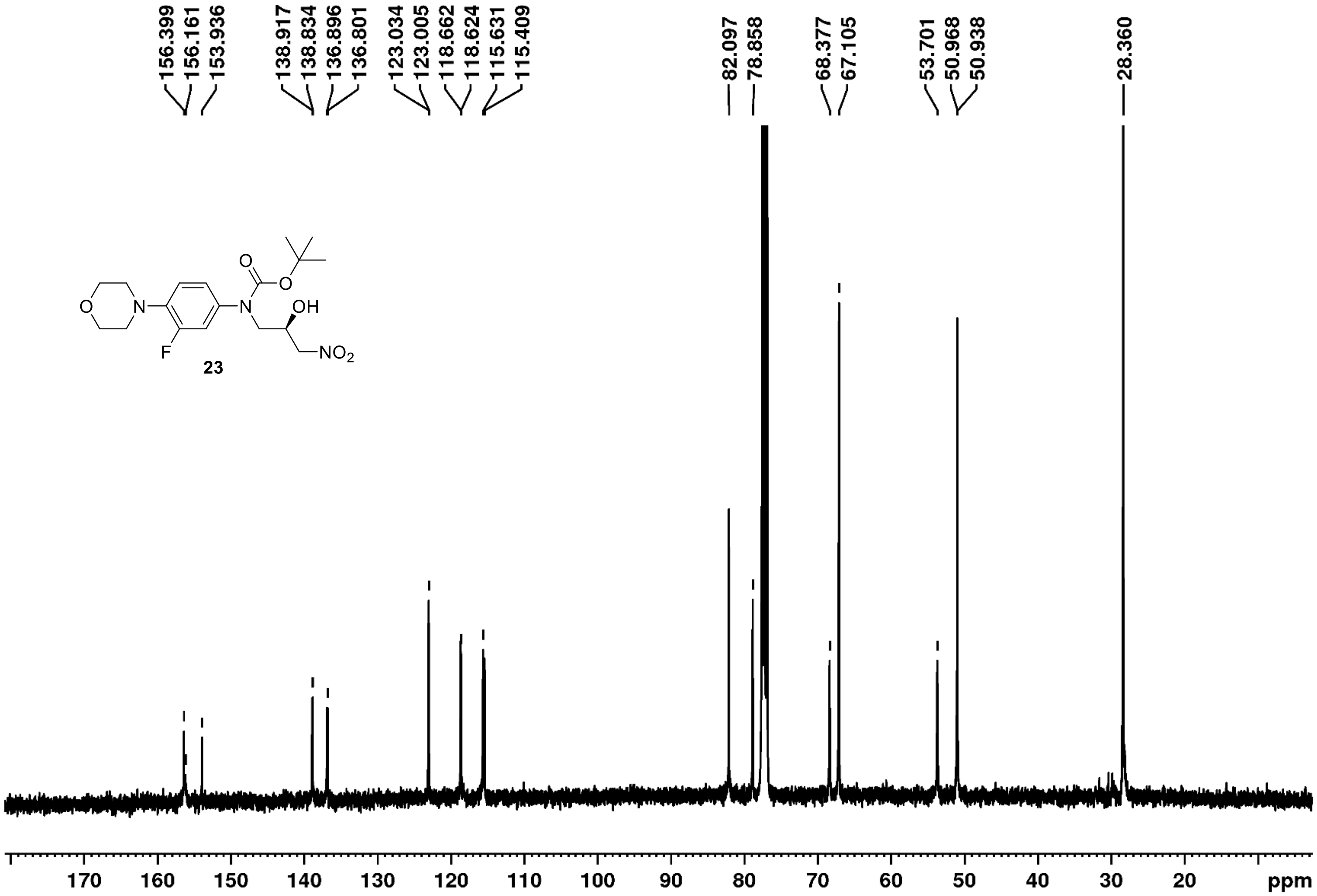

O
N
F
N
O
O
OH
NO2
23
156.399
156.161
153.936
138.917
138.834
136.896
136.801
123.034
123.005
118.662
118.624
115.631
115.409
82.097
78.858
68.377
67.105
53.701
50.968
50.938
28.360
170
160
150
140
130
120
110
100
90
80
70
60
50
40
30
20
ppm

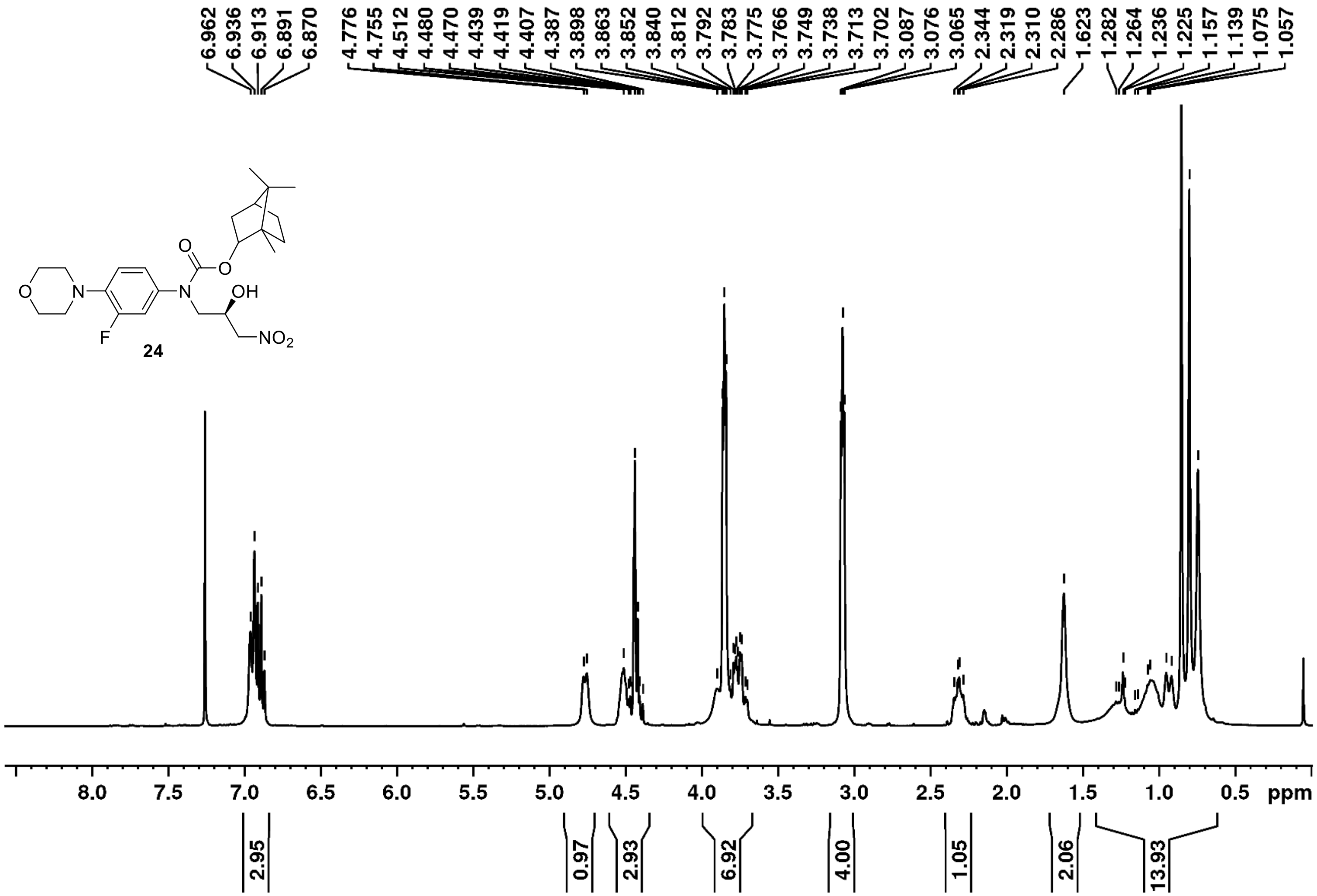

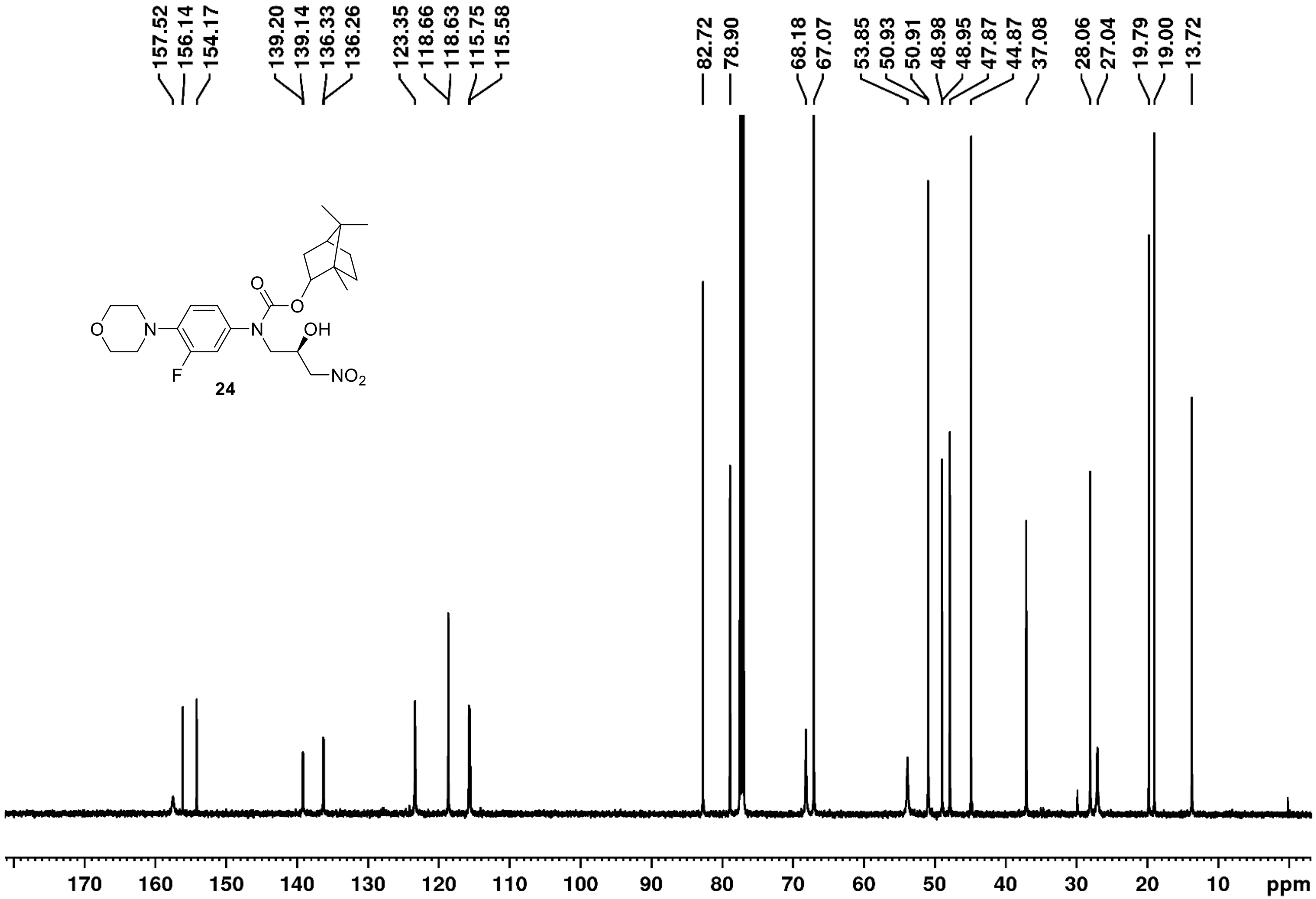
157.52
156.14
154.17
139.20
139.14
136.33
136.26
123.35
118.66
118.63
115.75
115.58
82.72
78.90
68.18
67.07
53.85
50.93
50.91
48.98
48.95
47.87
44.87
37.08
28.06
27.04
19.79
19.00
13.72
O
N
F
N
O
O
OH
NO2
24
170 160 150 140 130 120 110 100 90 80 70 60 50 40 30 20 10 ppm

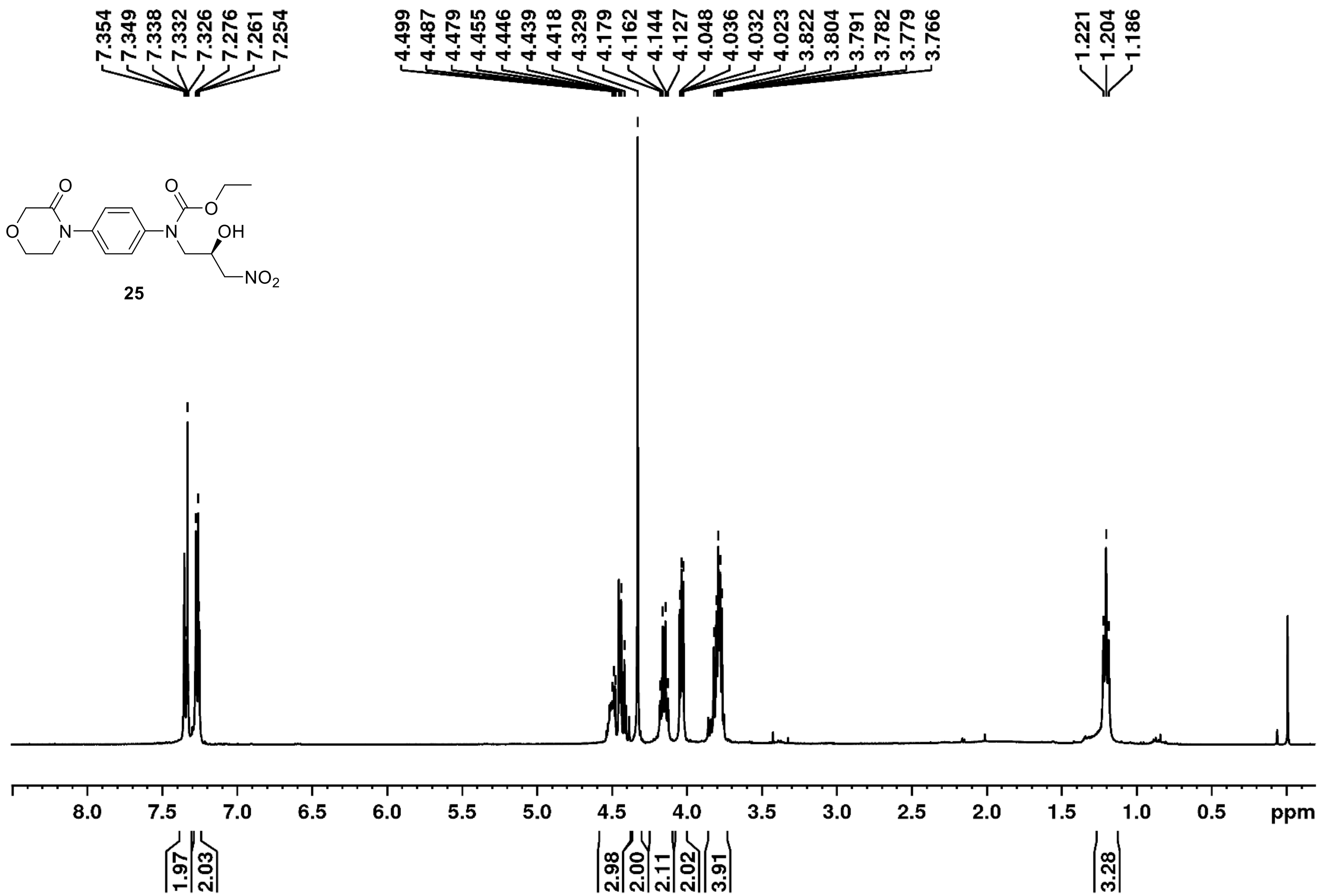
7.354
7.349
7.338
7.332
7.326
7.276
7.261
7.254
4.499
4.487
4.479
4.455
4.446
4.439
4.418
4.329
4.179
4.162
4.144
4.127
4.048
4.036
4.032
4.023
3.822
3.804
3.791
3.782
3.779
3.766
1.221
1.204
1.186
O
O
O
N
N
O
OH
NO2
25
8.0
7.5
7.0
6.5
6.0
5.5
5.0
4.5
4.0
3.5
3.0
2.5
2.0
1.5
1.0
0.5
ppm
1.97
2.03
2.98
2.00
2.11
2.02
3.91
3.28

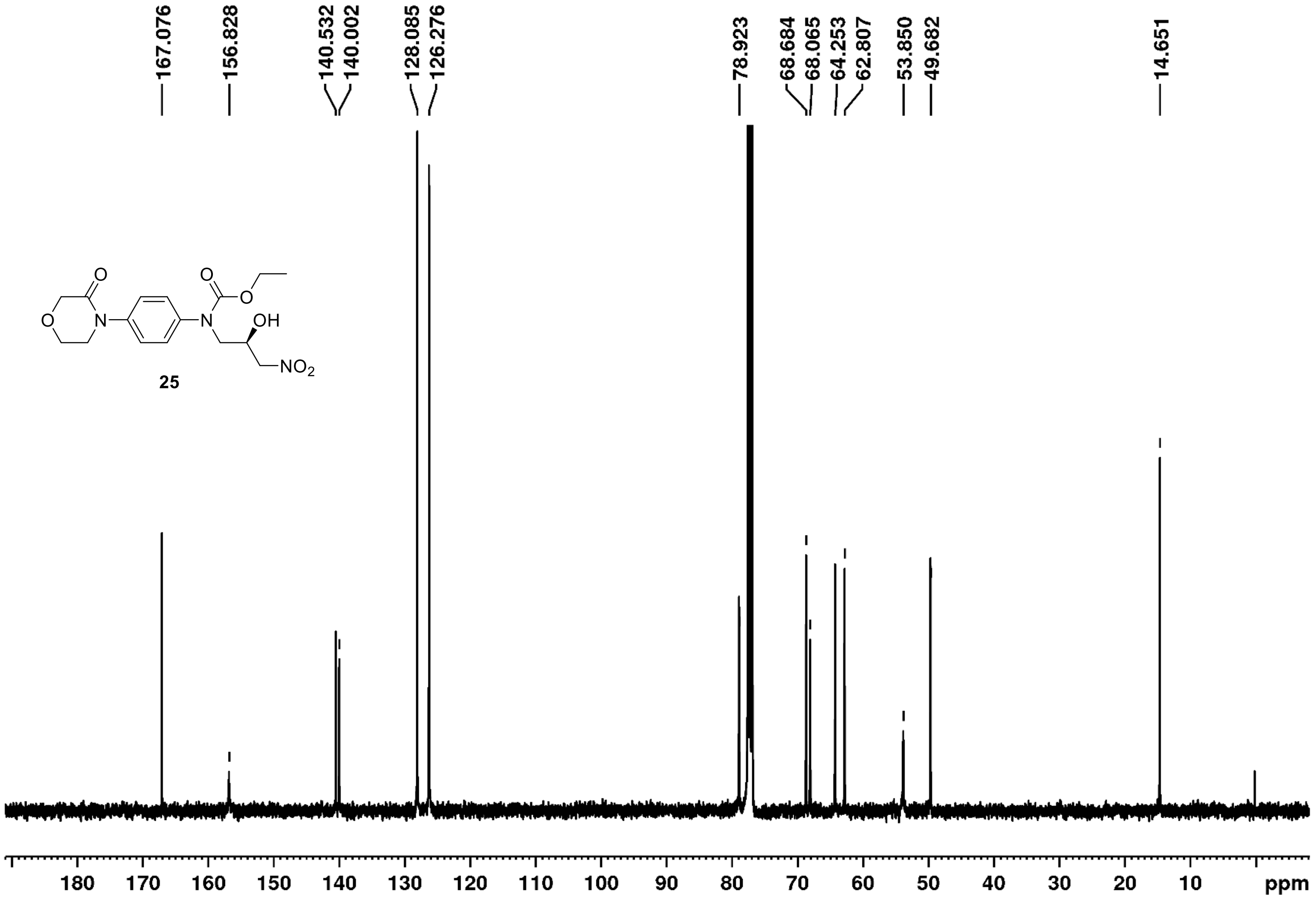
167.076
156.828
140.532
140.002
128.085
126.276
78.923
68.684
68.065
64.253
62.807
53.850
49.682
14.651
O
O
N
N
O
O
OH
NO2
25
180 170 160 150 140 130 120 110 100 90 80 70 60 50 40 30 20 10 ppm

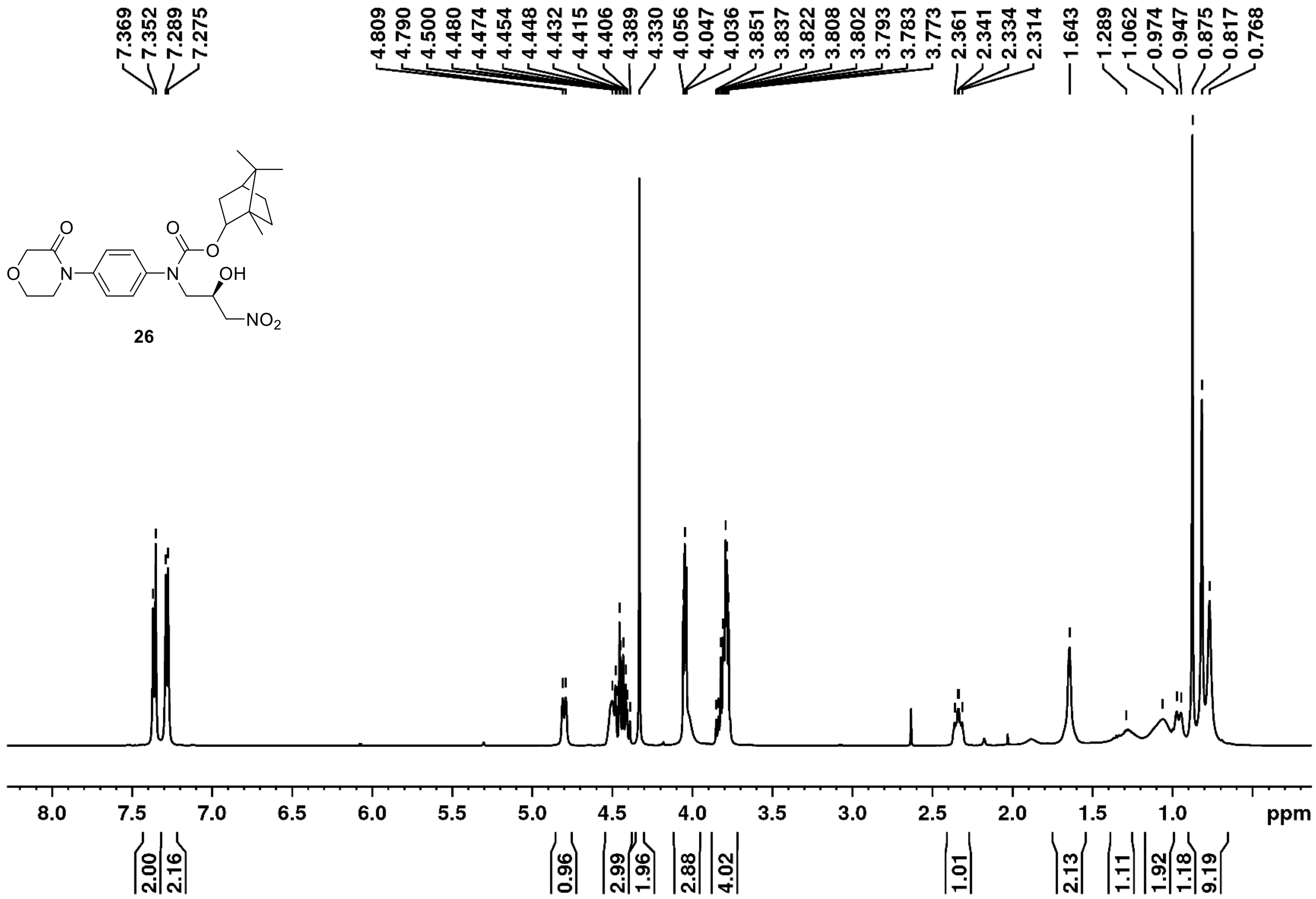
26
ppm
8.0 7.5 7.0 6.5 6.0 5.5 5.0 4.5 4.0 3.5 3.0 2.5 2.0 1.5 1.0
7.369 7.352 7.289 7.275
4.809 4.790 4.500 4.480 4.474 4.454 4.448 4.432 4.415 4.406 4.389 4.330 4.056 4.047 4.036 3.851 3.837 3.822 3.808 3.802 3.793 3.783 3.773 2.361 2.341 2.334 2.314 1.643 1.289 1.062 0.974 0.947 0.875 0.817 0.768
2.00 2.16 0.96 2.99 1.96 2.88 4.02 1.01 2.13 1.11 1.92 1.18 9.19

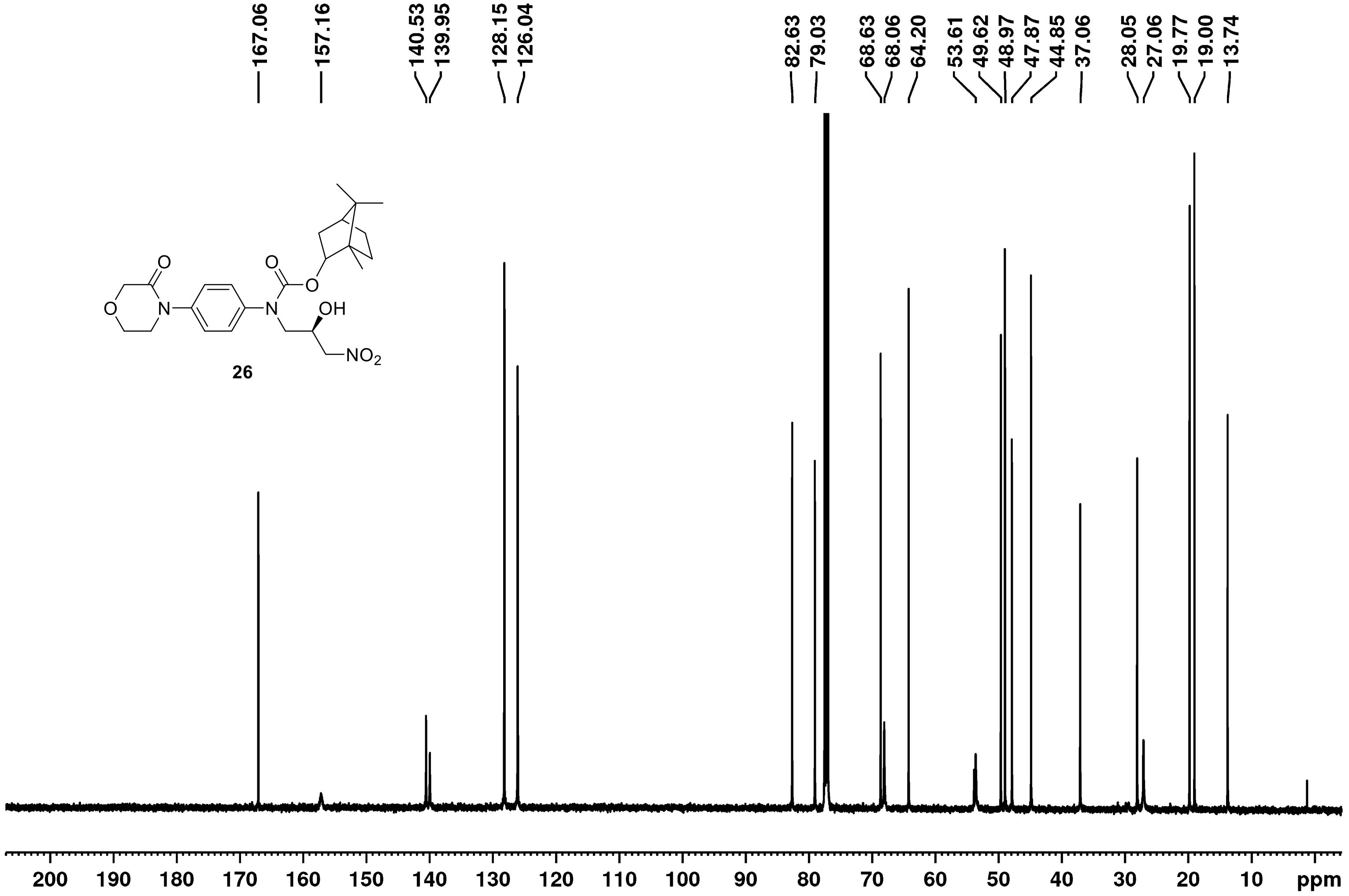
167.06
157.16
140.53
139.95
128.15
126.04
82.63
79.03
68.63
68.06
64.20
53.61
49.62
48.97
47.87
44.85
37.06
28.05
27.06
19.77
19.00
13.74
O
O
N
N
O
O
OH
NO_2
26
200 190 180 170 160 150 140 130 120 110 100 90 80 70 60 50 40 30 20 10 ppm

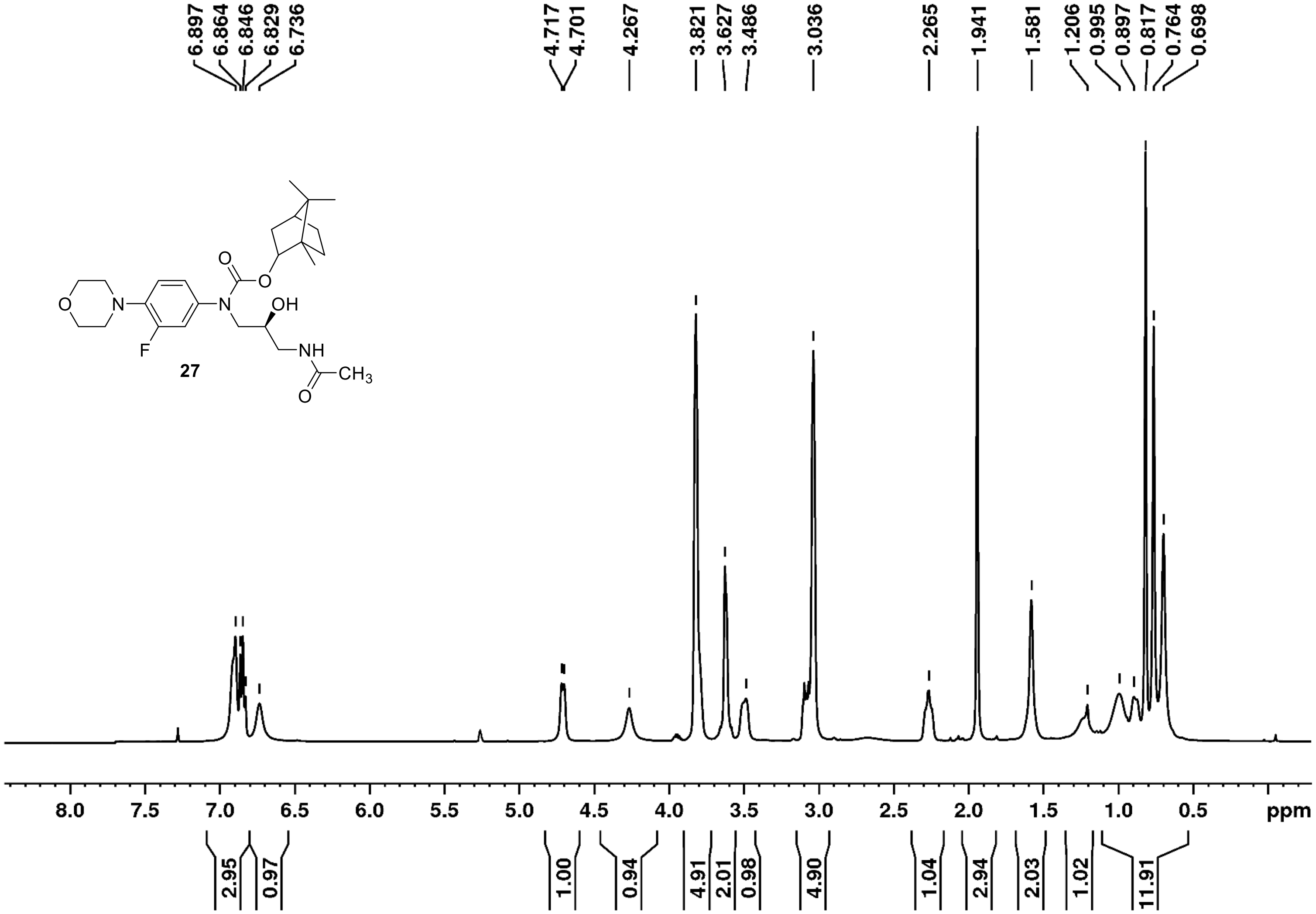
6.897
6.864
6.846
6.829
6.736
4.717
4.701
4.267
3.821
3.627
3.486
3.036
2.265
1.941
1.581
1.206
0.995
0.897
0.817
0.764
0.698
O
N
F
N
O
O
OH
NH
CH3
O
27
8.0
7.5
7.0
6.5
6.0
5.5
5.0
4.5
4.0
3.5
3.0
2.5
2.0
1.5
1.0
0.5
ppm
2.95
0.97
1.00
0.94
4.91
2.01
0.98
4.90
1.04
2.94
2.03
1.02
11.91

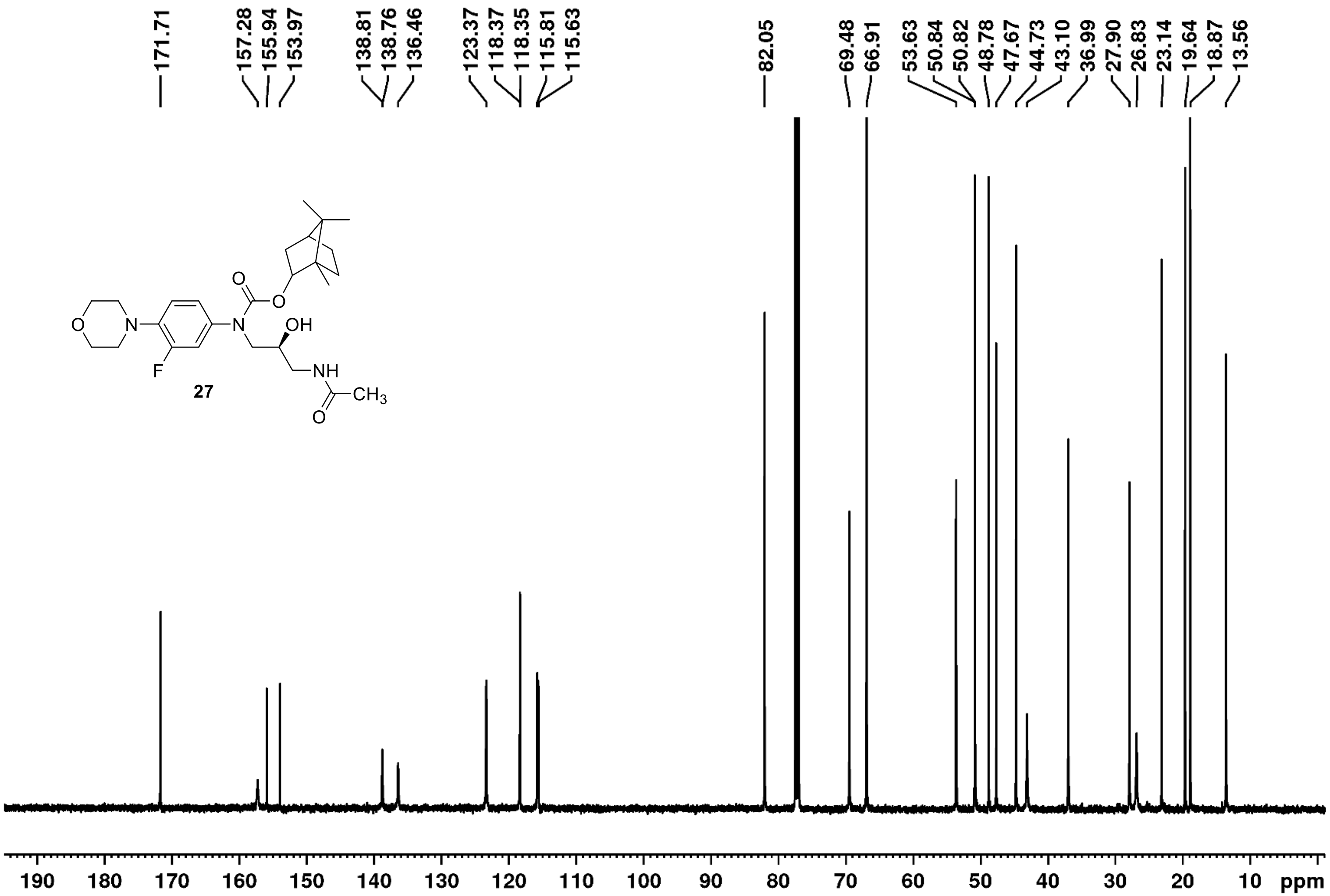
171.71
157.28
155.94
153.97
138.81
138.76
136.46
123.37
118.37
118.35
115.81
115.63
82.05
69.48
66.91
53.63
50.84
50.82
48.78
47.67
44.73
43.10
36.99
27.90
26.83
23.14
19.64
18.87
13.56
O
N
F
O
O
OH
NH
CH3
O
27
190 180 170 160 150 140 130 120 110 100 90 80 70 60 50 40 30 20 10 ppm

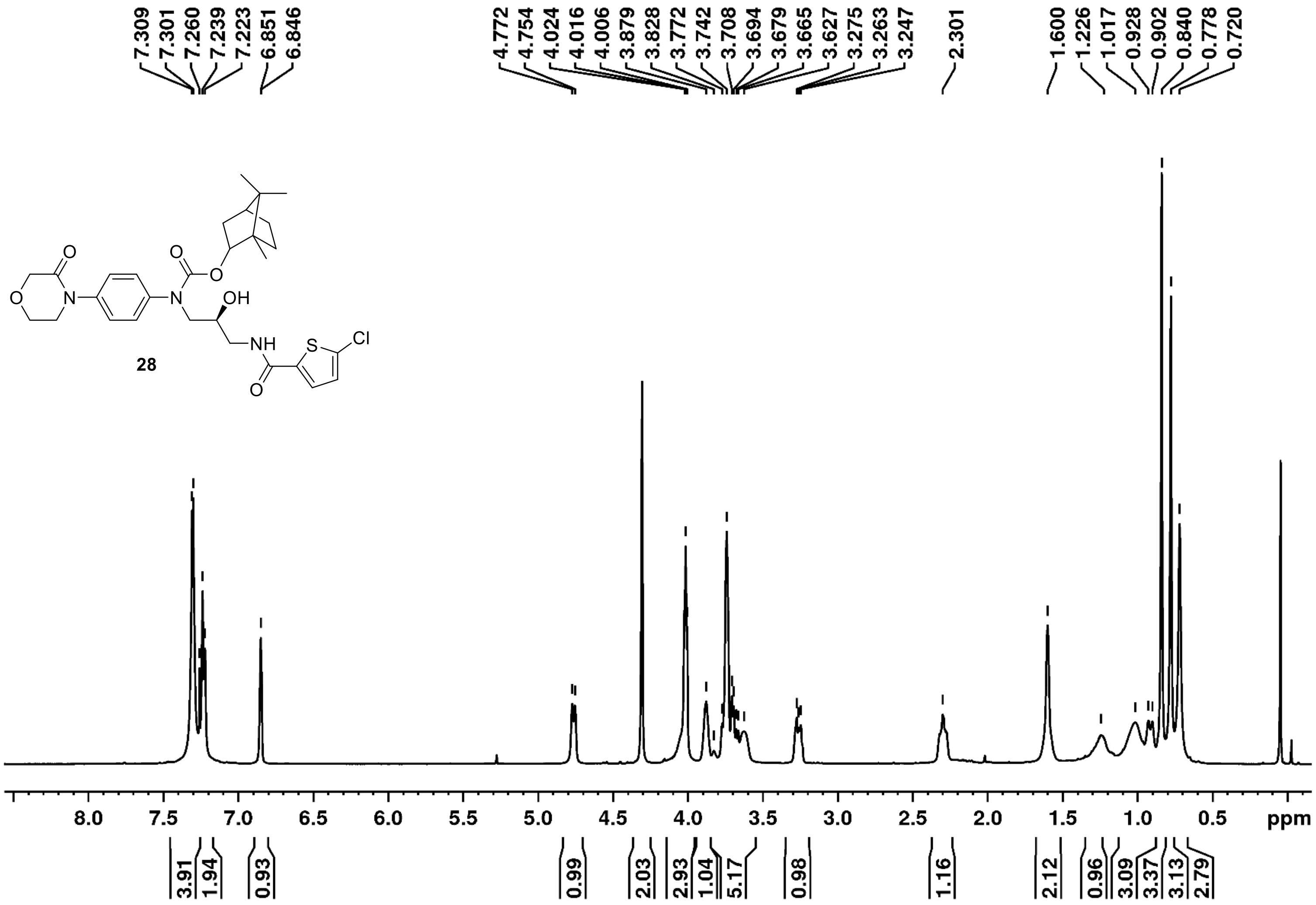
7.309
7.301
7.260
7.239
7.223
6.851
6.846
4.772
4.754
4.024
4.016
4.006
3.879
3.828
3.772
3.742
3.708
3.694
3.679
3.665
3.627
3.275
3.263
3.247
2.301
1.600
1.226
1.017
0.928
0.902
0.840
0.778
0.720
28
ppm
8.0
7.5
7.0
6.5
6.0
5.5
5.0
4.5
4.0
3.5
3.0
2.5
2.0
1.5
1.0
0.5
3.91
1.94
0.93
0.99
2.03
2.93
1.04
5.17
0.98
1.16
2.12
0.96
3.09
3.37
3.13
2.79

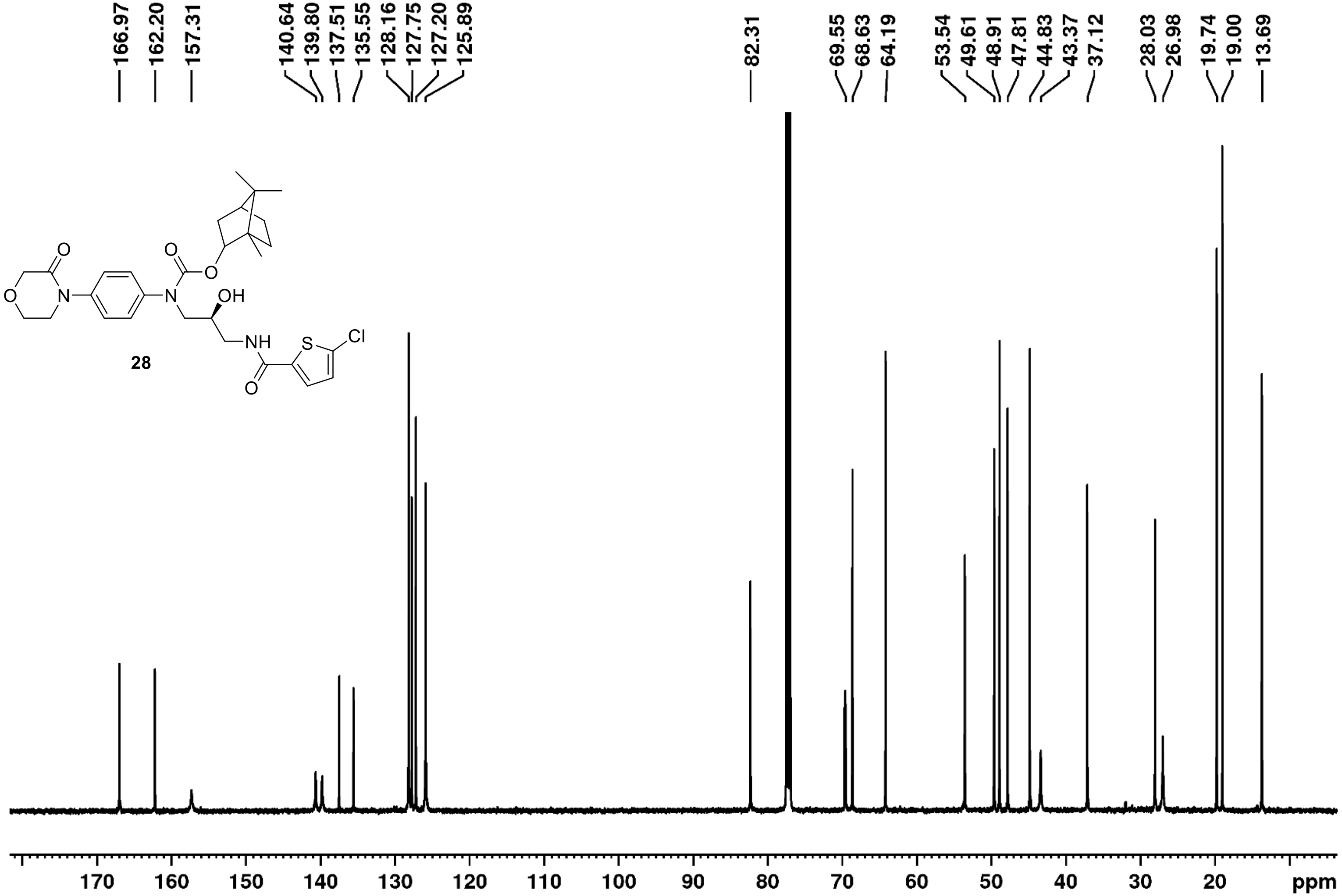
166.97
162.20
157.31
140.64
139.80
137.51
135.55
128.16
127.75
127.20
125.89
82.31
69.55
68.63
64.19
53.54
49.61
48.91
47.81
44.83
43.37
37.12
28.03
26.98
19.74
19.00
13.69
O
O
N
N
O
O
OH
NH
S
Cl
O
170
160
150
140
130
120
110
100
90
80
70
60
50
40
30
20
ppm

28

3. HPLC data

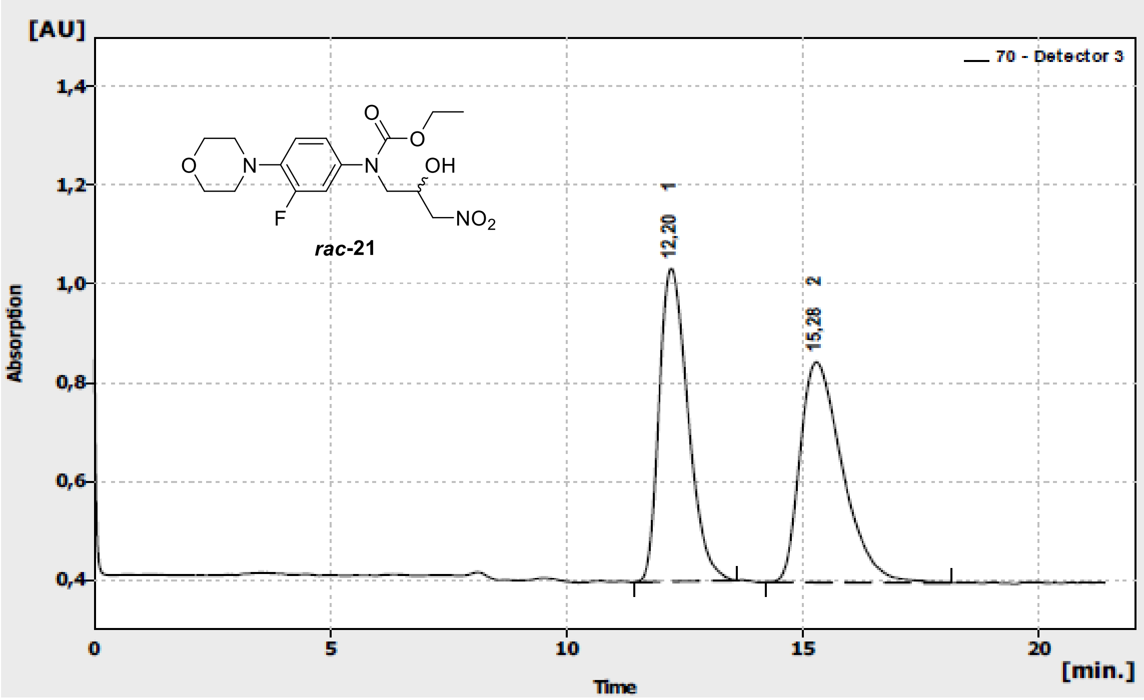

Result Table (Uncal - 70 - Detector 3)

	Reten. Time [min]	Area [mAU.s]	Height [mAU]	Area [%]	Height [%]	W05 [min]	Peak Purity [-]	Compound Name
1	12,200	27037,970	635,553	49,1	58,7	0,68	691	
2	15,275	28038,874	447,672	50,9	41,3	0,96	613	
	Total	55076,844	1083,225	100,0	100,0			

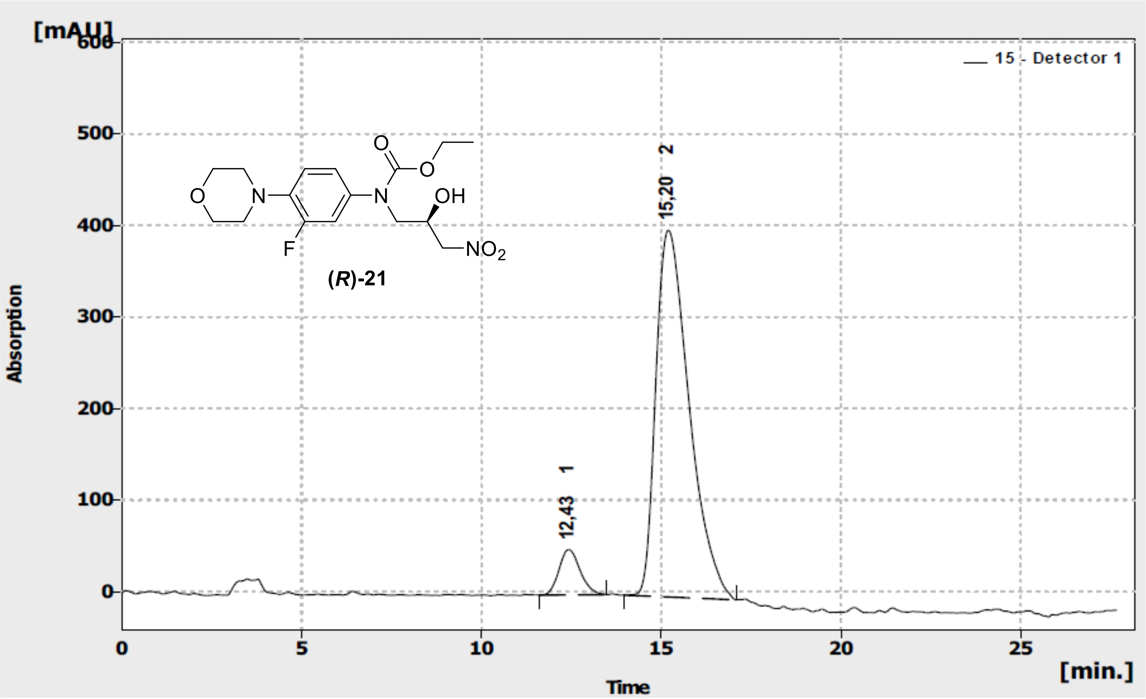

Result Table (Uncal - 15 - Detector 1)

	Reten. Time [min]	Area [mAU.s]	Height [mAU]	Area [%]	Height [%]	W05 [min]	Peak Purity [-]	Compound Name
1	12,433	2035,590	49,468	7,3	11,0	0,64	687	
2	15,200	25796,244	400,603	92,7	89,0	1,00	800	
	Total	27831,834	450,071	100,0	100,0			

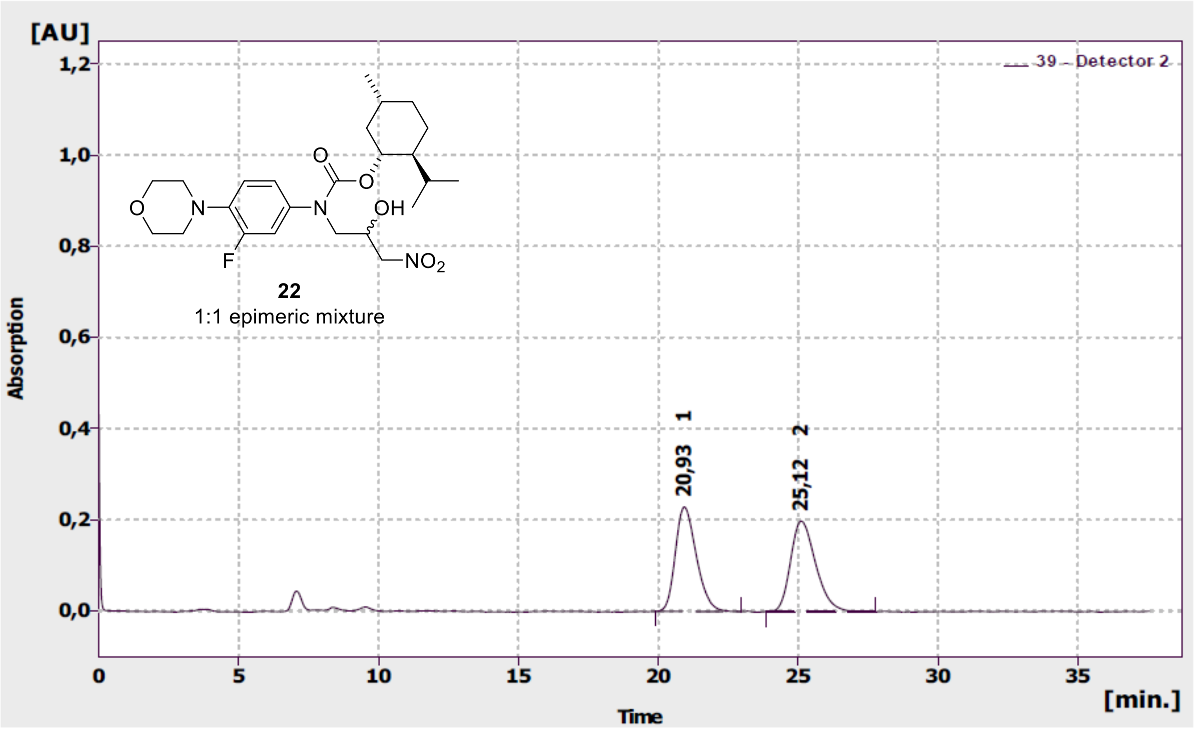

Result Table (Uncal - 39 - Detector 2)

	Reten. Time [min]	Area [mAU.s]	Height [mAU]	Area [%]	Height [%]	W05 [min]	Peak Purity [-]	Compound Name
1	20,933	11986,723	229,086	48,9	53,5	0,82	716	
2	25,117	12508,763	199,107	51,1	46,5	0,97	708	
	Total	24495,486	428,192	100,0	100,0			

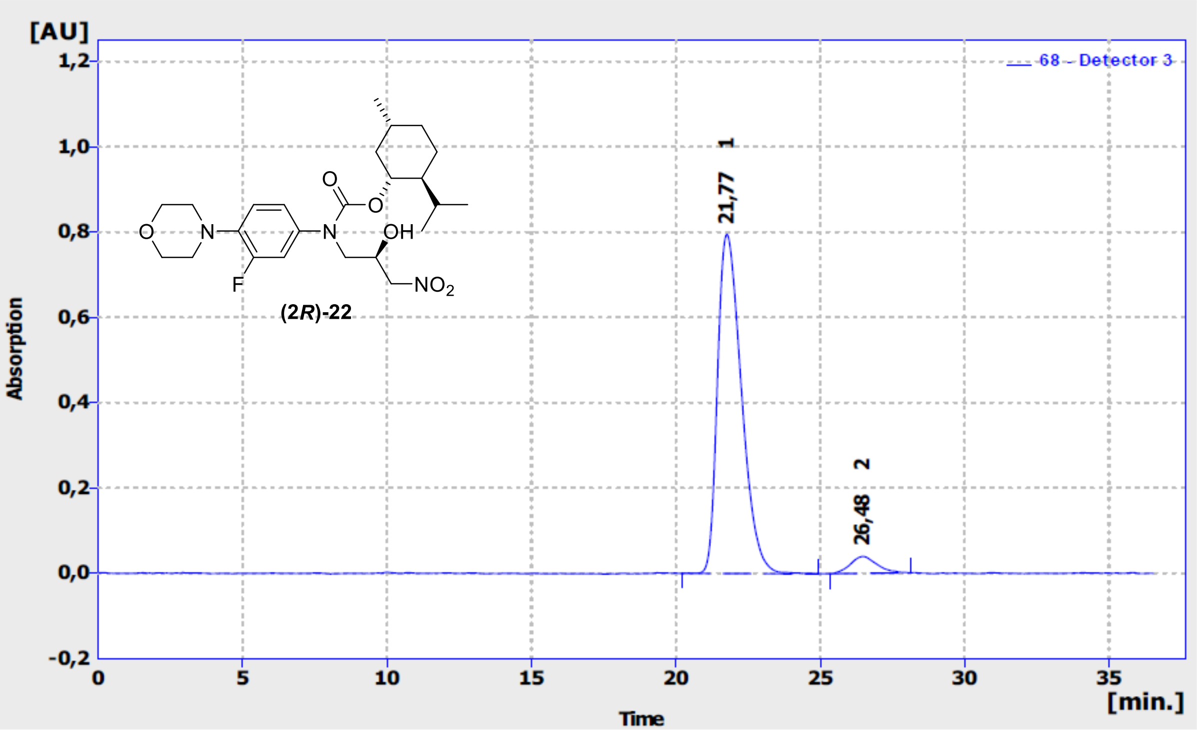

Result Table (Uncal - 68 - Detector 3)

	Reten. Time [min]	Area [mAU.s]	Height [mAU]	Area [%]	Height [%]	W05 [min]	Peak Purity [-]	Compound Name
1	21,767	45862,935	795,319	94,7	95,2	0,90	689	
2	26,475	2560,851	39,713	5,3	4,8	0,99	885	
	Total	48423,786	835,032	100,0	100,0			

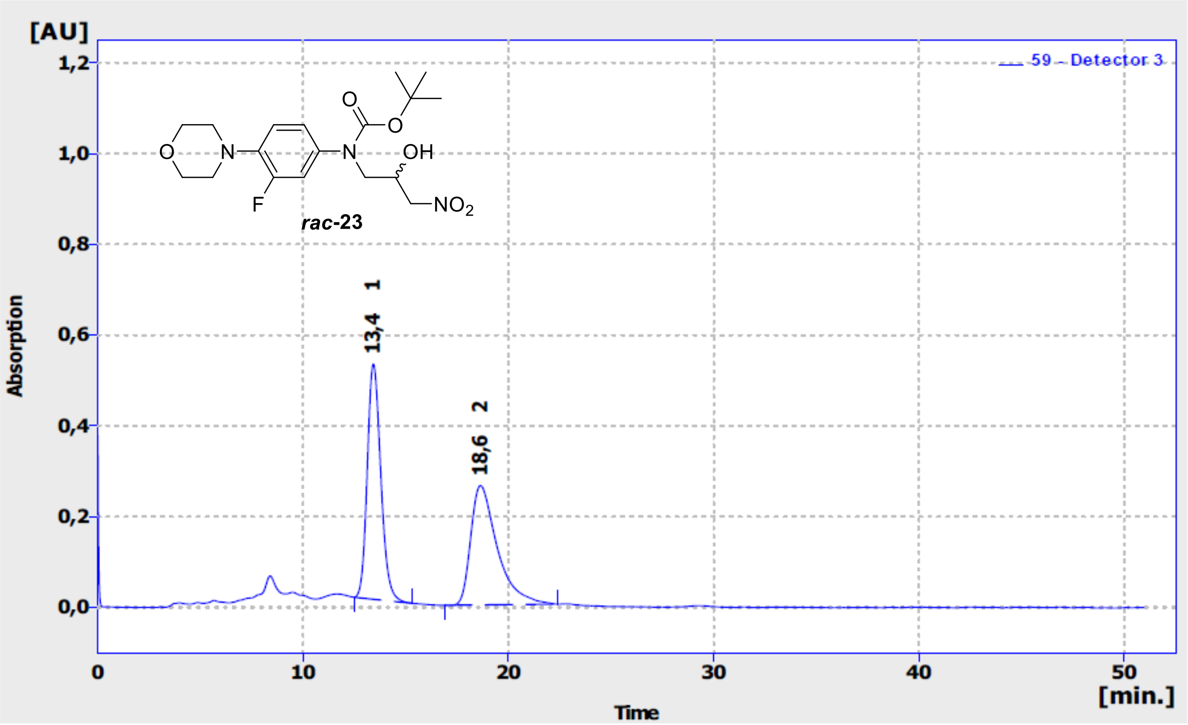

Result Table (Uncal - 59 - Detector 3)

	Reten. Time [min]	Area [mAU.s]	Height [mAU]	Area [%]	Height [%]	W05 [min]	Peak Purity [-]	Compound Name
1	13,425	23746,742	518,428	50,1	66,3	0,71	977	
2	18,642	23673,158	262,967	49,9	33,7	1,32	931	
	Total	47419,900	781,395	100,0	100,0			

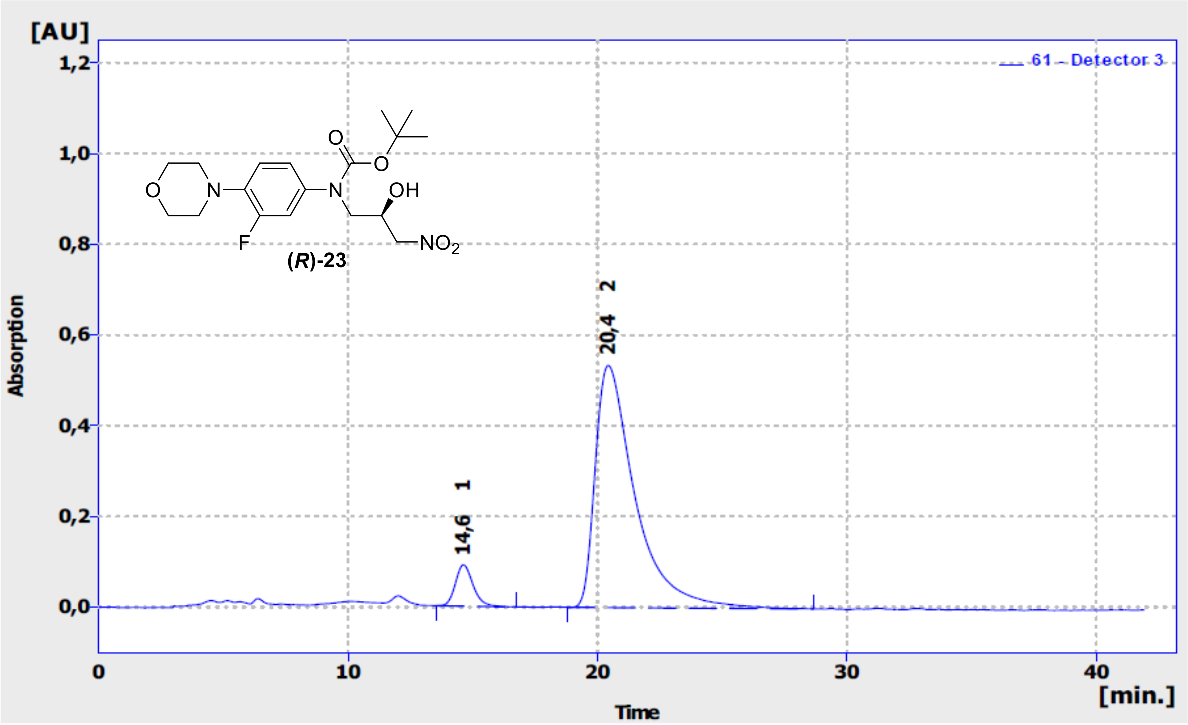

Result Table (Uncal - 61 - Detector 3)

	Reten. Time [min]	Area [mAU.s]	Height [mAU]	Area [%]	Height [%]	W05 [min]	Peak Purity [-]	Compound Name
1	14,625	4507,347	91,102	7,0	14,6	0,75	630	
2	20,433	59483,566	533,921	93,0	85,4	1,58	667	
	Total	63990,913	625,023	100,0	100,0			

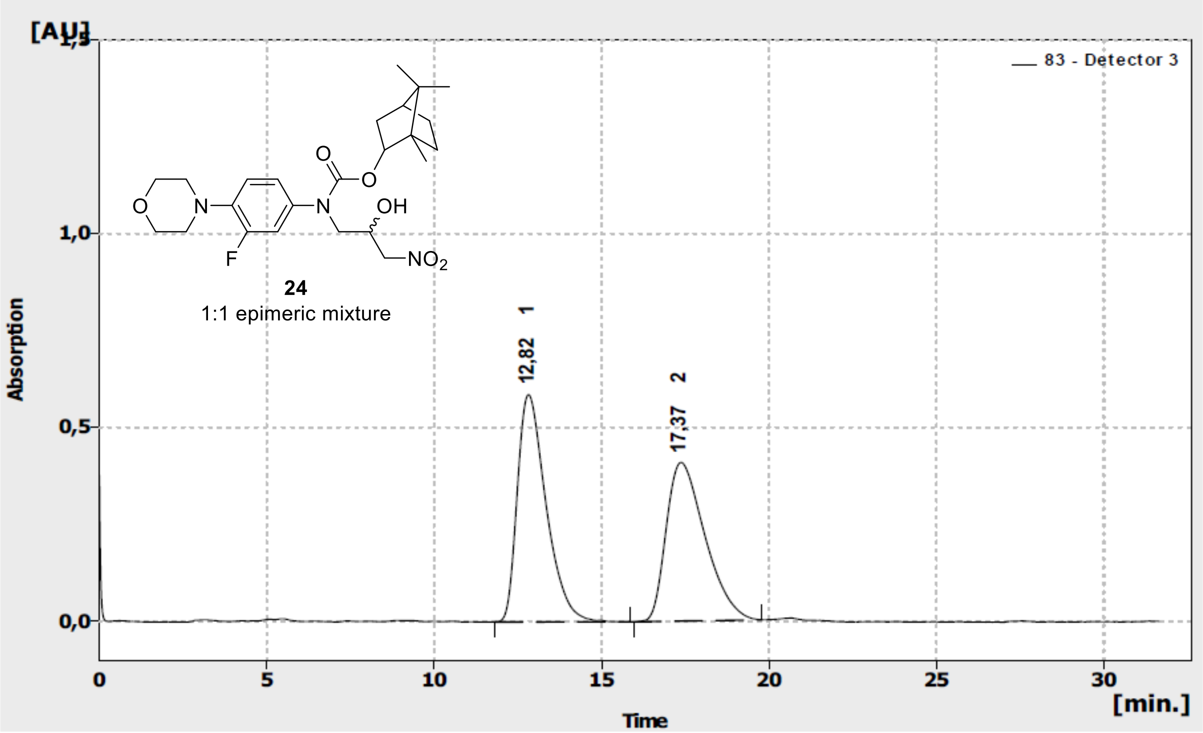

Result Table (Uncal - 83 - Detector 3)

	Reten. Time [min]	Area [mAU.s]	Height [mAU]	Area [%]	Height [%]	W05 [min]	Peak Purity [-]	Compound Name
1	12,825	35036,715	586,377	51,5	58,9	0,92	776	
2	17,367	32938,125	408,961	48,5	41,1	1,27	893	
	Total	67974,840	995,338	100,0	100,0			

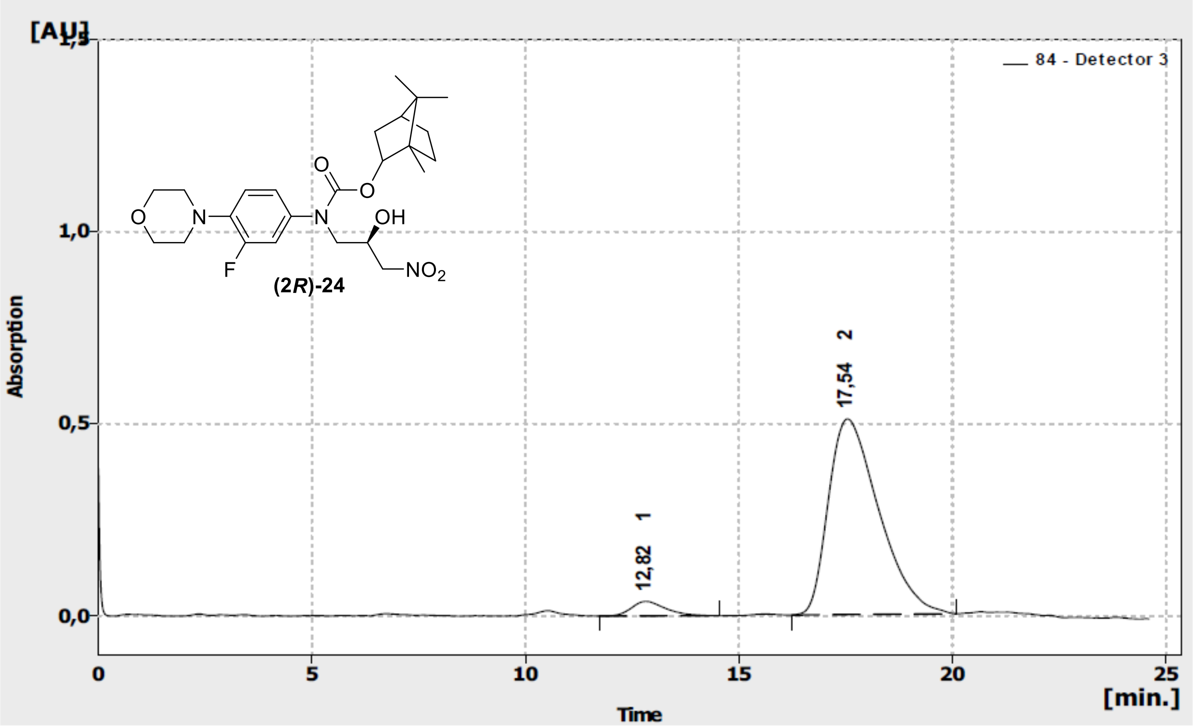

Result Table (Uncal - 84 - Detector 3)

	Reten. Time [min]	Area [mAU.s]	Height [mAU]	Area [%]	Height [%]	W05 [min]	Peak Purity [-]	Compound Name
1	12,825	2141,332	37,917	4,9	6,9	0,86	880	
2	17,542	41866,554	509,149	95,1	93,1	1,28	947	
	Total	44007,886	547,067	100,0	100,0			

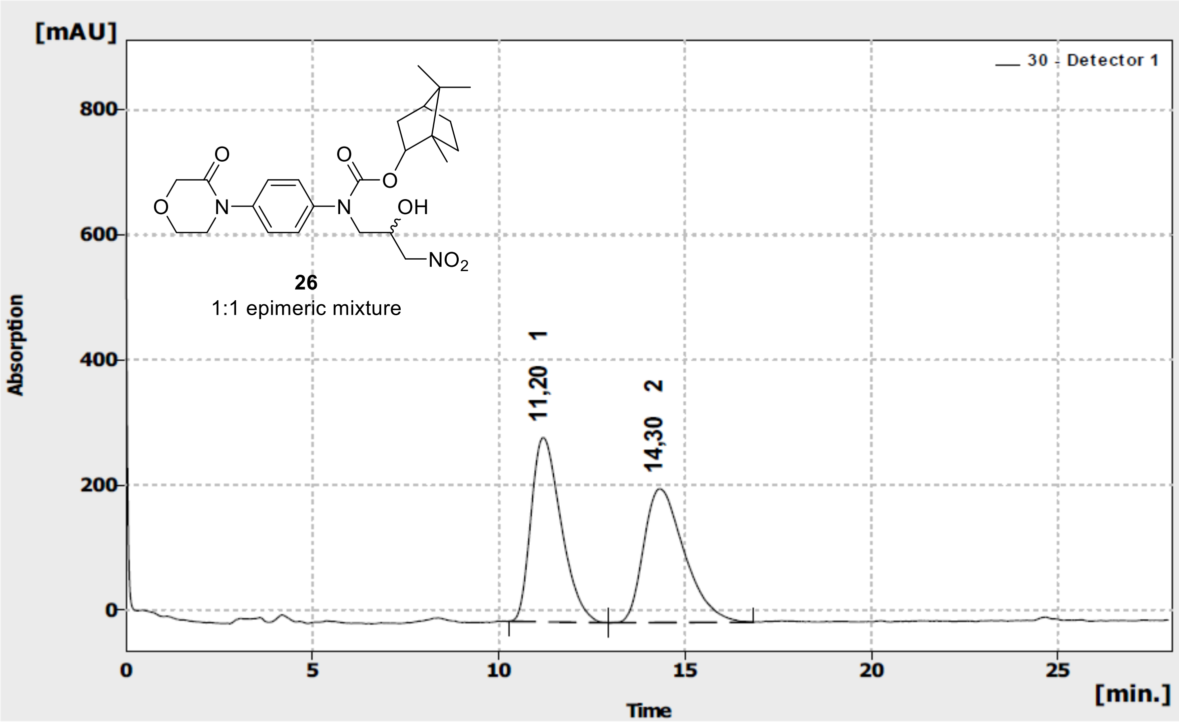

Result Table (Uncal - 30 - Detector 1)

	Reten. Time [min]	Area [mAU.s]	Height [mAU]	Area [%]	Height [%]	W05 [min]	Peak Purity [-]	Compound Name
1	11,200	16559,923	294,959	50,5	58,0	0,88	822	
2	14,300	16248,785	214,021	49,5	42,0	1,18	799	
	Total	32808,707	508,980	100,0	100,0			

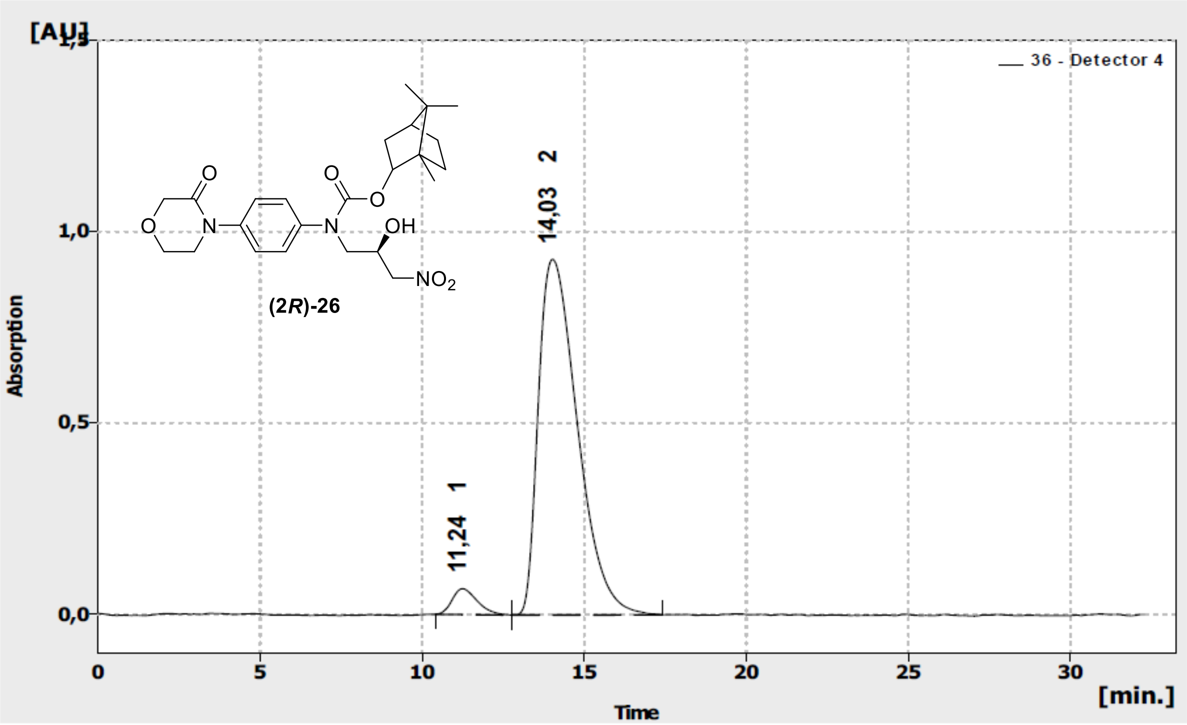

Result Table (Uncal - 36 - Detector 4)

	Reten. Time [min]	Area [mAU.s]	Height [mAU]	Area [%]	Height [%]	W05 [min]	Peak Purity [-]	Compound Name
1	11,242	3633,646	67,615	4,5	6,8	0,83	837	
2	14,033	77810,016	929,130	95,5	93,2	1,32	794	
	Total	81443,662	996,746	100,0	100,0			

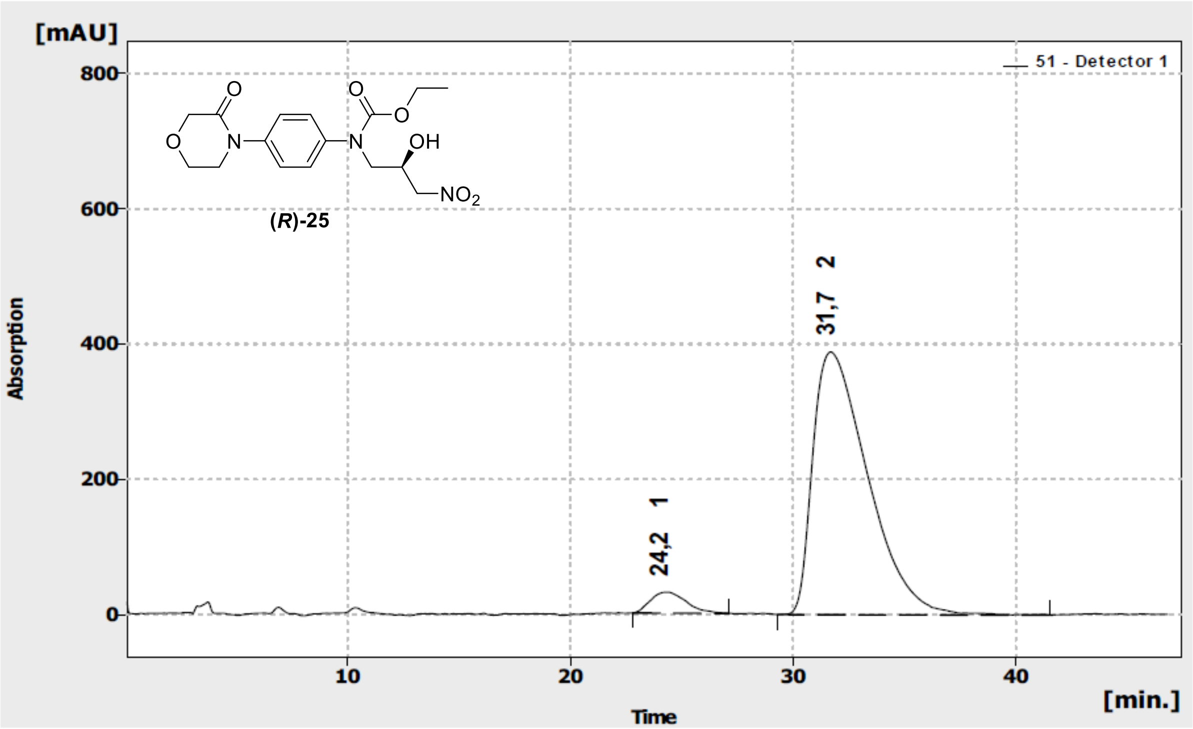

Result Table (Uncal - 51 - Detector 1)

	Reten. Time [min]	Area [mAU.s]	Height [mAU]	Area [%]	Height [%]	W05 [min]	Peak Purity [-]	Compound Name
1	24,233	3470,100	31,124	4,9	7,4	1,79	928	
2	31,700	66645,037	388,601	95,1	92,6	2,67	697	
	Total	70115,137	419,726	100,0	100,0			

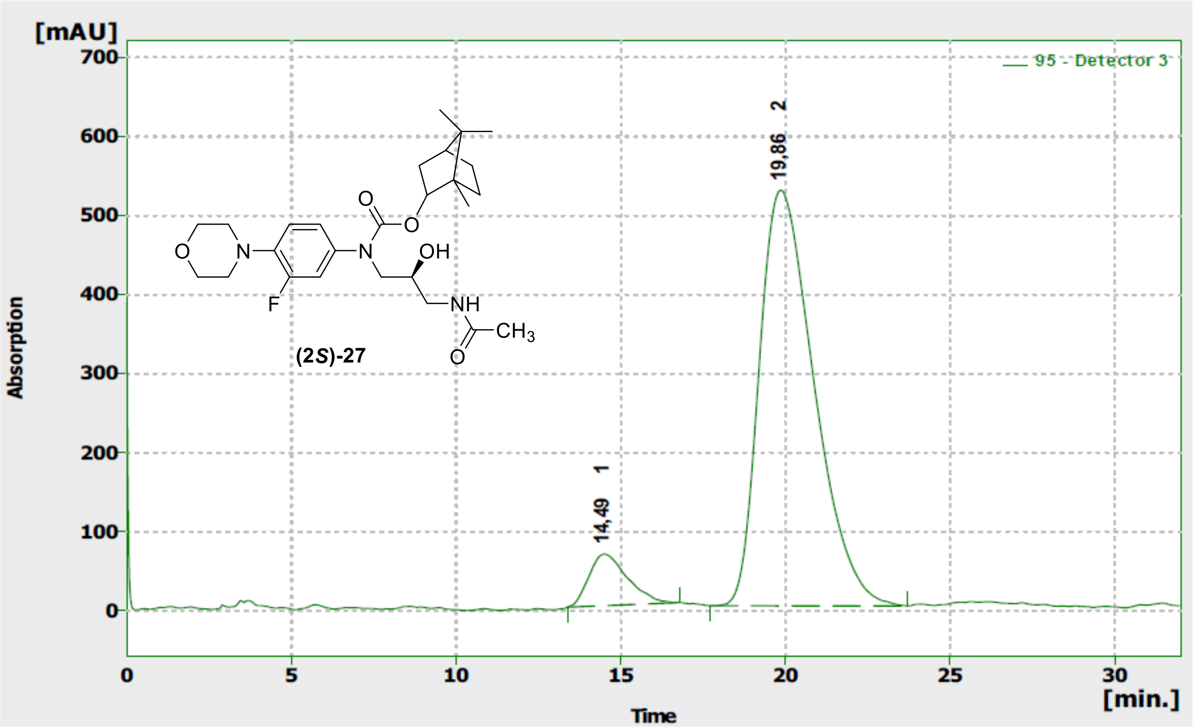

Result Table (Uncal - 95 - Detector 3)

	Reten. Time [min]	Area [mAU.s]	Height [mAU]	Area [%]	Height [%]	W05 [min]	Peak Purity [-]	Compound Name
1	14,492	5317,817	65,417	8,1	11,1	1,29	967	
2	19,858	60700,059	525,984	91,9	88,9	1,81	940	
	Total	66017,876	591,401	100,0	100,0			

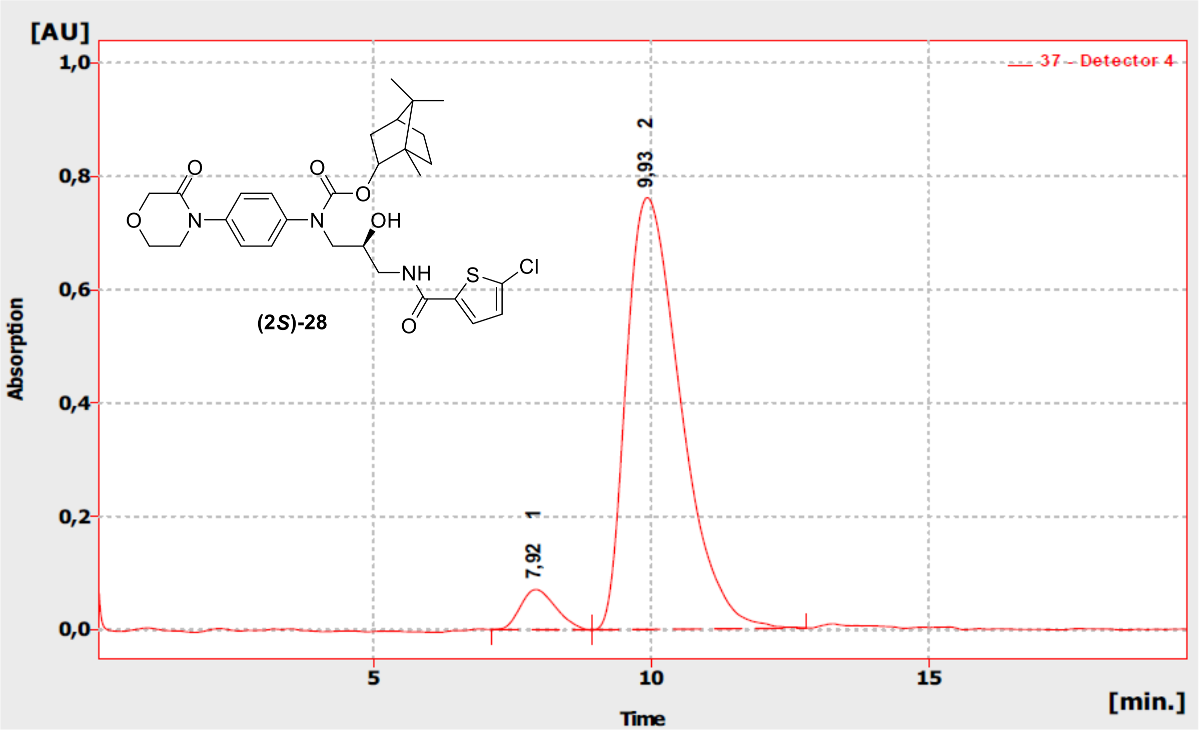

Result Table (Uncal - 37 - Detector 4)

	Reten. Time [min]	Area [mAU.s]	Height [mAU]	Area [%]	Height [%]	W05 [min]	Peak Purity [-]	Compound Name
1	7,925	3129,505	70,897	5,7	8,5	0,72	832	
2	9,933	51804,432	761,406	94,3	91,5	1,07	913	
	Total	54933,938	832,303	100,0	100,0			

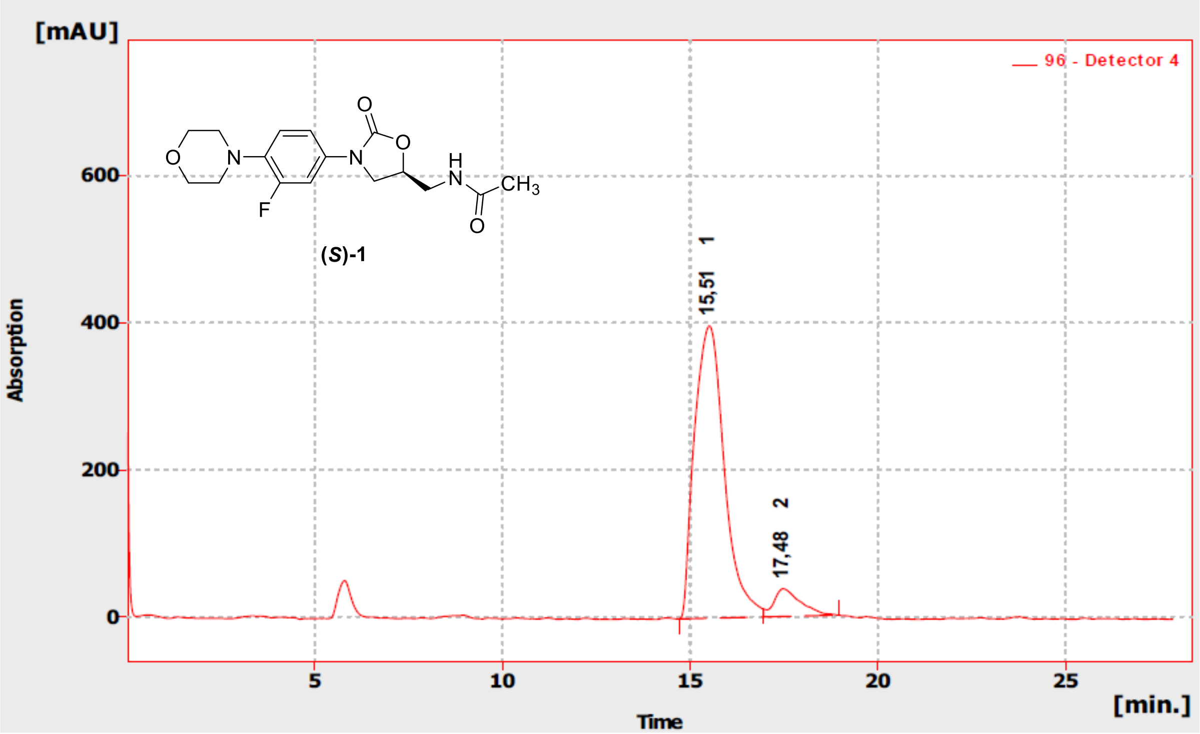

Result Table (Uncal - 96 - Detector 4)

	Reten. Time [min]	Area [mAU.s]	Height [mAU]	Area [%]	Height [%]	W05 [min]	Peak Purity [-]	Compound Name
1	15,508	21982,922	397,664	92,1	91,3	0,89	956	
2	17,483	1896,283	37,840	7,9	8,7	0,76	986	
	Total	23879,205	435,504	100,0	100,0			

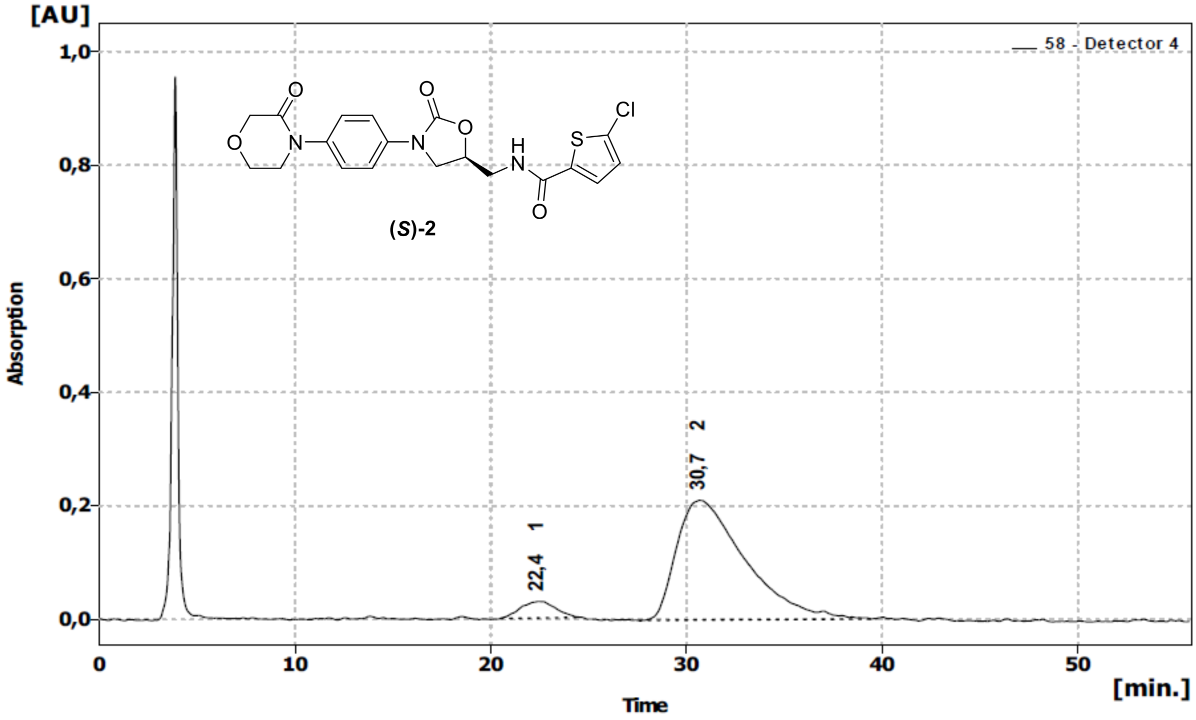

Result Table (Uncal - 58 - Detector 4)

	Reten. Time [min]	Area [mAU.s]	Height [mAU]	Area [%]	Height [%]	W05 [min]	Peak Purity [-]	Compound Name
1	22,425	3647,885	29,148	6,6	12,1	2,04	877	
2	30,692	51405,760	211,041	93,4	87,9	3,70	830	
	Total	55053,645	240,189	100,0	100,0			

4. References

[1] Shen, Y.; Sun, Y.; Sang, Z.; Sun, C.; Dai, Y.; Deng, Y. *Molecules* **2012**, *17*, 8661–8673.

[2] Brickner, S. J.; Hutchinson, D. K.; Barbachyn, M. R.; Manninen, P. R.; Ulanowicz, D. A.; Garmon, S. A.; Grega, K. C.; Hendges, S. K.; Toops, D. S.; Ford, C. W.; Zurenko, G. E. *J. Med. Chem.* **1996**, *39*, 673–679.

[3] Piccionello, A. P.; Pierro, P.; Accardo, A.; Buscemi, S.; Pace, A. *RSC Adv.* **2013**, *3*, 24946–24951.

[4] Colombo, L.; Allegrini, P.; Brusasca, M.; D´arienzo, G.; Razzetti, G. A process for the preparation of oxazolidinone derivatives. EP 2072505A2, June 24, 2009.

[5] Drabina, P.; Feixová, V.; Sedlák, M. *Tetrahedron Lett.* **2019**, *60*, 99–101.

[6] Morán-Ramallal, R.; Liz, R.; Gotor, V. *Org. Lett.* **2008**, *10*, 1935–1938.

[7] Fattah, T. A.; Saeed, A. *Tetrahedron: Asymmetry* **2017**, *28*, 485–504.
